# Substrate Peptidomimetic Inhibitors of *P. falciparum* Plasmepsin X with Potent Antimalarial Activity

**DOI:** 10.1002/cmdc.202200306

**Published:** 2022-08-18

**Authors:** Lachlan W. Richardson, Trent D. Ashton, Madeline G. Dans, Nghi Nguyen, Paola Favuzza, Tony Triglia, Anthony N. Hodder, Anna Ngo, Kate E. Jarman, Alan F. Cowman, Brad E. Sleebs

**Affiliations:** ^1^ Walter and Eliza Hall Institute of Medical Research Parkville 3052 Victoria Australia; ^2^ Department of Medical Biology University of Melbourne Parkville 3010 Victoria Australia

**Keywords:** *Plasmodium*, malaria, plasmepsin, aspartyl protease, peptidomimetic

## Abstract

Plasmepsin X (PMX) is an aspartyl protease that processes proteins essential for *Plasmodium* parasites to invade and egress from host erythrocytes during the symptomatic asexual stage of malaria. PMX substrates possess a conserved cleavage region denoted by the consensus motif, SF*h*E (*h*=hydrophobic amino acid). Peptidomimetics reflecting the P_3_‐P_1_ positions of the consensus motif were designed and showed potent and selective inhibition of PMX. It was established that PMX prefers Phe in the P_1_ position, di‐substitution at the β‐carbon of the P_2_ moiety and a hydrophobic P_3_ group which was supported by modelling of the peptidomimetics in complex with PMX. The peptidomimetics were shown to arrest asexual *P. falciparum* parasites at the schizont stage by impairing PMX substrate processing. Overall, the peptidomimetics described will assist in further understanding PMX substrate specificity and have the potential to act as a template for future antimalarial design.

## Graphical abstract

**Substrate mimicry**: Substrate peptidomimetics were designed and showed potent inhibition of plasmepsin X (PMX). The peptidomimetics block PMX ligand processing, arrest asexual parasites at the schizont stage and potently kill *P. falciparum* parasites. The peptidomimetics will further assist in the understanding of PMX selectivity and in the development of PMX targeted antimalarials.

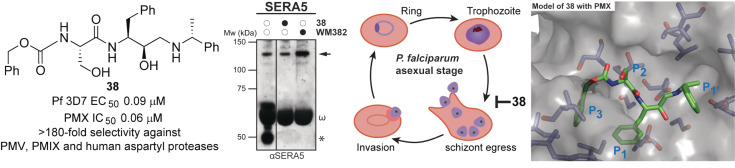

## Introduction

Malaria is a devastating human disease resulting an estimated 214 million infections and 405,000 deaths in 2020.^[1]^ Malaria in humans is caused by five *Plasmodium* species. *P. falciparum* causes most of the malaria associated morbidity and mortality in sub‐Saharan Africa. *P. vivax* is responsible for symptom relapse post drug treatment and is most frequently encountered in Southeast Asia and South America.

Current frontline curative treatments for malaria are a combination of quinoline and artemisinin‐based agents. Resistance to quinoline antimalarials is widespread, and there has been a recent increase in resistance against artemisinin (ART) therapies.[^2^, ^3^] Consequently, the World Health Organisation has stipulated that new drug classes against novel targets are needed to combat the rising emergence of drug resistant malaria strains worldwide.^[4]^

The *P. falciparum* genome encodes ten cathepsin D‐like aspartyl proteases called plasmepsins (PMs).^[5]^ The PMs are differentially expressed across the *P. falciparum* lifecycle and several are known to be essential for parasite survival and pathogenesis.^[6]^ PMs I–IV are digestive vacuole proteases that are not required for parasite survival.[^7^, ^8^, ^9^] PMs VI–VIII are expressed in mosquitos where they may have important roles in parasite development.[^10^, ^11^] PMV is located in the endoplasmic reticulum in the asexual blood stage^[12]^ and cleaves the *Plasmodium* export element (PEXEL)[^13^, ^14^] on cargo proteins destined for the host erythrocyte during host‐cell remodelling.[^15^, ^16^] PMV is essential for protein export and parasite survival[^17^, ^18^] and therefore is a promising multi‐lifecycle stage antimalarial drug target.[^19^, ^20^, ^21^, ^22^, ^23^, ^24^]

PMIX and PMX have high homology, and both are essential for asexual parasite development. PMIX is expressed in the late asexual blood stage and critically processes rhoptry associated proteins, such as rhoptry associated protein 1 (RAP1) and apical sushi protein (ASP) that mediate secretory function in merozoite invasion of the host erythrocyte (Figure S1).[^25^, ^26^, ^27^] PMX processes surface invasion ligands, such as apical membrane protein 1 (AMA1) critical for parasite invasion of the erythrocyte.[^25^, ^26^, ^28^] PMX processes and activates proteases, such as subtilisin 1 (SUB1), a maturase of key downstream effectors, including merozoite surface protein 1 (MSP1) and serine repeat antigen (SERA5) critical for parasite egress from the erythrocyte. PMX also cleaves ligands essential for parasite pathogenesis in transmission and liver stages (Figure S1),[^25^, ^26^, ^28^] and therefore is considered an attractive multi‐stage antimalarial target.^[29]^

**Figure 1:**
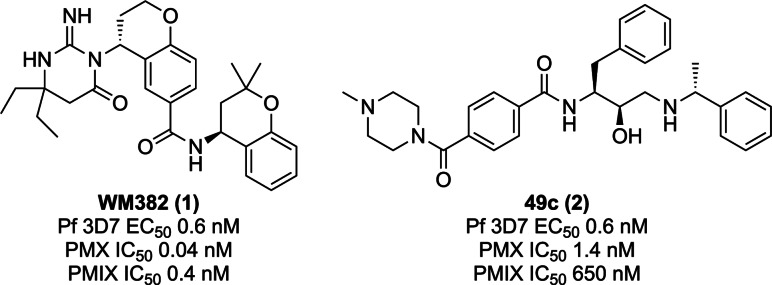
Structures and activities of dual plasmepsin IX/X inhibitors described in literature.[^26^, ^28^, ^30^, ^31^]

Towards the development of an antimalarial targeting PMX, several small molecule inhibitors of PMX have recently been reported. Notably, WM382 (**1**) potently inhibits PMX and PMIX,[^27^, ^28^] while 49c (**2**) potently inhibits PMX and has >500‐fold selectivity for PMX compared to PMIX (Figure 1).[^26^, ^31^] Both these tool compounds utilise head groups previously employed to target aspartyl proteases. WM382 (**1**) employs the imino pyrimidinone head group^[32]^ originally used to target human beta‐secretase 1 (BACE1) and renin,[^33^, ^34^] whereas 49c (**2**) utilizes the hydroxyethyl amine (HEA) scaffold, a substrate transition state mimetic previously exploited by inhibitors targeting HIV aspartyl protease^[35]^ and *Plasmodium* plasmepsins.[^30^, ^36^, ^37^] In independent studies, WM382 (**1**) and 49c (**2**), were used as tool compounds to pharmacologically validate the role of PMIX in *P. falciparum* erythrocytic invasion, and the function of PMX in erythrocytic invasion and egress, in addition to critical events in transmission and liver stages of malaria.

PMX matures invasion and egress substrates at a cleavage site often denoted by the consensus sequence, SF*h*E (where *h* is a hydrophobic amino acid). Specifically, PMX processes substrates on the C‐terminal side of the P_1_ Phe. To date, there has been a limited number of biochemical studies investigating the substrate specificity of PMX,[^26^, ^28^] and no reports of peptidomimetics directly mimicking the substrate sequence of PMX.

**Figure 2:**
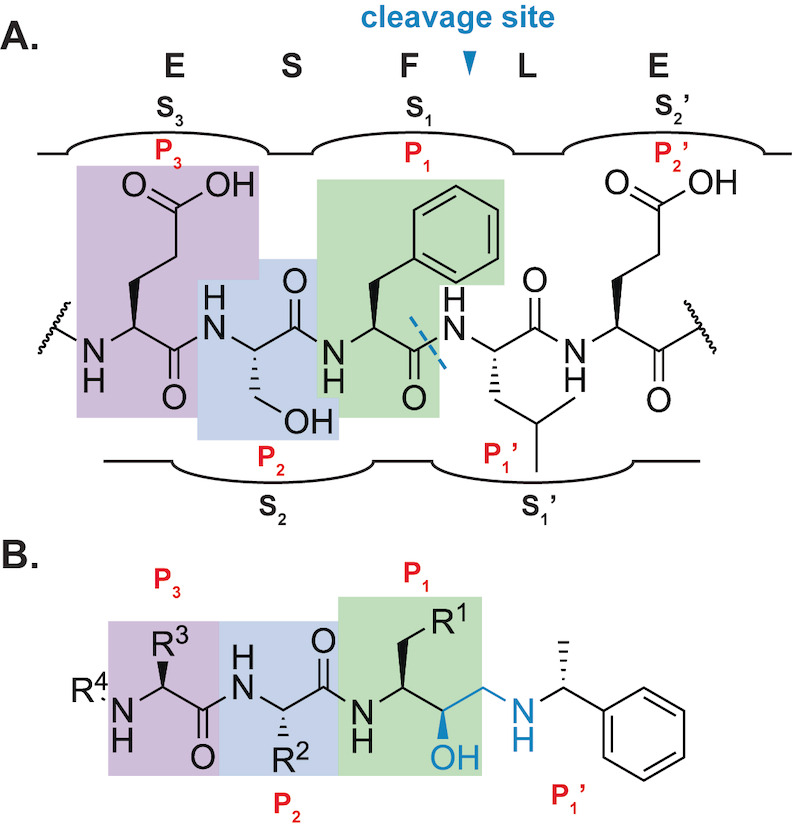
Compound design rationale. **A**. A typical PMX recognition motif (RAP1) possessing the conserved SF*h*E consensus sequence with the cleavage site (light blue dashed line) and binding pockets (S_x_) shown, with substrate amino acids (P_x_) highlighted purple, blue and green with respect to panel B. **B**. The general structure of the proposed P_3_ ‐ P_1_’ peptidomimetic compounds, showing P_3_, P_2_ and P_1_ amino acids (highlighted purple, blue and green, respectively) concerning the native PMX substrate amino acid positions.

Here in we design and synthesize peptidomimetics to mimic the natural substrates of PMX. To do this, we employed the HEA scaffold that mimics the transition state of the aspartyl catalytic dyad and the P_1_ amino acid while the adjoining amino acids mimic the adjacent P_2_ and P_3_ positions (Figure 2). To help map PMX substrate specificity and selectivity, the peptidomimetics were evaluated biochemically against plasmepsin V and IX and human aspartyl proteases. A focal set of compounds were then used to show the relationship between biochemical PMX inhibition and on‐target activity in *P. falciparum* asexual parasites. The present study aims to assist in understanding PMX substrate specificity and may support the design of more effective PMX inhibitors.

## Results and Discussion

**Peptidomimetic design rationale**. Peptidomimetic inhibitors were designed to further interrogate the binding site preferences of PMX and generate tools to better understand the role of PMX in parasite survival. The HEA scaffold depicted in the structure of 49c (**2**) in Figure 2 was chosen as the template for peptidomimetic design. Target compound design was rationalized using known and predicted PMX substrate cleavage data (Table S1 and Figure S2). Collectively, these data emphasized the importance of the P_1_ and P_2_ residues for binding recognition and suggested that Phe was preferred in the P_1_ position and Ser was preferred in the P_2_ position. Two target sets were designed to investigate PMX binding. The first target compound scaffold mimicked the P_3_‐P_1_ positions, whilst the second target scaffold mimicked the P_2_‐P_1_ positions (Figure 2). For each scaffold, a combination of each P_3_‐P_1_ amino acids from the SF*h*E substrate consensus sequence were selected (denoted by R groups that correspond to the side chain positions in Figure 2) to determine their binding preference for PMX. The amino acids incorporated into the P_3_ position were informed by the proteome search and consensus sequence abundance plot, with Ile, Asn and Ser chosen (Figure S2). The P_1_’ position of the target compounds incorporated the C‐terminal (*R*)‐(+)‐α‐methylbenzylamino motif from the PMX inhibitor 49c (Figures 1 and 2).[^26^, ^30^]

**Scheme 1:**
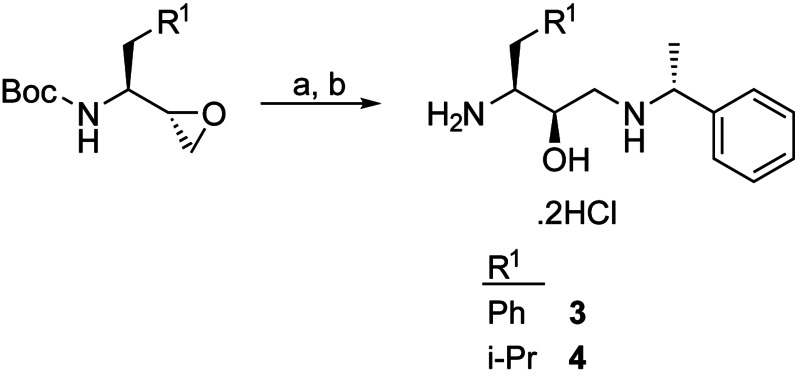
Synthesis of the hydroxyethyl amine synthon. *Reagents and conditions*: a) (*R*)‐(+)‐α‐methylbenzylamine, MeOH, reflux 48 h. b) 4 N HCl, dioxane, 30 min. R=*i*‐Pr or phenyl.

**Peptidomimetic synthesis**. The synthesis of peptidomimetic compounds began with the generation of two HEA scaffolds representing the P_1_ amino acids Phe and Leu. The synthesis started by using (*R*)‐(+)‐α‐methylbenzylamine as a nucleophile in an epoxide opening with (2*R*,3*S*)‐1,2‐epoxy‐3‐(Boc‐amino)‐4‐phenylbutane or (2*R*,3*S*)‐1,2‐epoxy‐3‐(Boc‐amino)‐4‐isopropylbutane to give the *N*‐Boc protected HEA compounds **3** and **4**, respectively. Subsequent removal of the Boc‐group was affected using 4 N HCl to afford HEA building blocks **3** and **4** (Scheme 1).

**Scheme 2:**
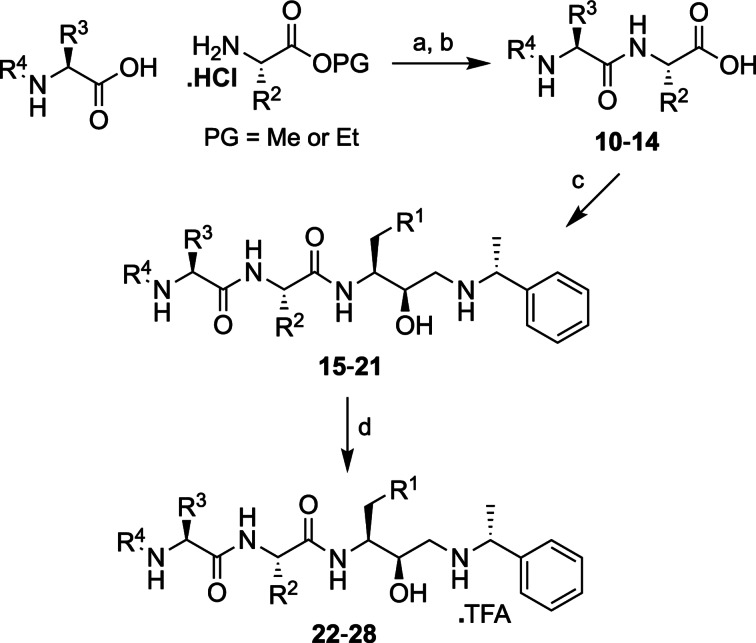
Synthesis of P_3_‐P_1_ peptidomimetic analogues. *Reagents and conditions*: a) HBTU, DIPEA, DMF, 18 h, 20 °C. b) LiOH, THF or MeOH, H_2_O. c) **3** or **4**, HBTU, DIPEA, DMF, 18 h, 20 °C. d) TFA, DCM, 4–6 h, 20 °C. For R^1^, R^2^, R^3^ and R^4^ refer to Table 1.

**Scheme 3:**
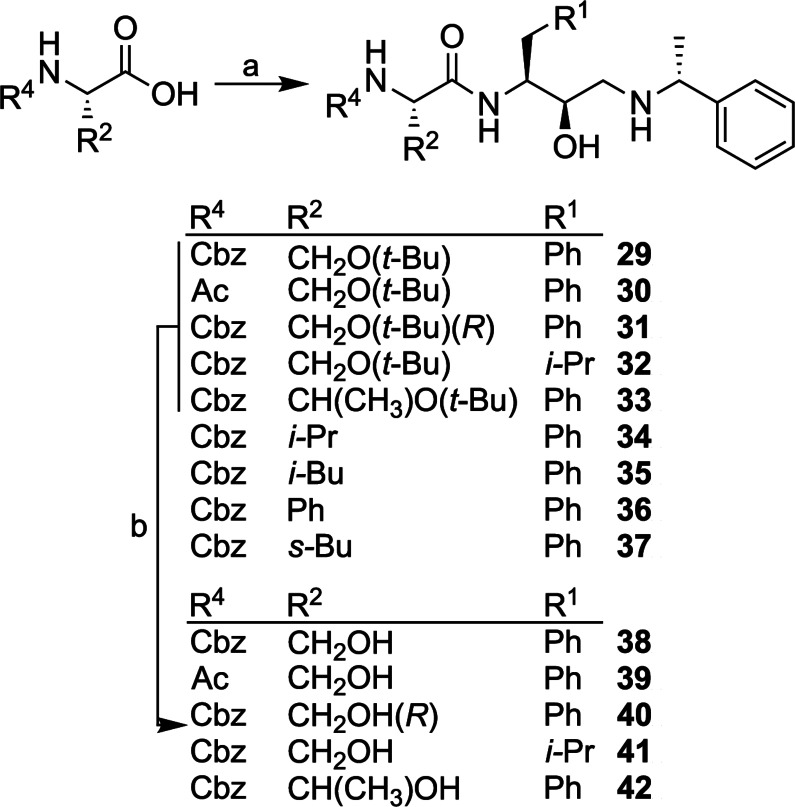
Synthesis of P_2_‐P_1_ peptidomimetic analogues. *Reagents and conditions*: a) **3** or **4**, HBTU, DIPEA, DMF, 18 h, 20 °C. b) TFA, DCM, 4–6 h, 20 °C.

The synthesis of the P_3_‐P_1_ analogues and P_2_‐P_1_ analogues used conventional solution phase peptide coupling methods (Schemes 2 and 3). The synthesis of P_3_‐P_1_ analogues began with the formation of the ester protected dipeptides **5**–**9**, of varying R^2^ and R^3^ functionality using HBTU peptide coupling conditions. The ester functionality of the dipeptides **5**–**9** was subsequently hydrolyzed using LiOH to afford the carboxylic acids **10**–**14**, which were then coupled with the HEA building blocks, **3** and **4**. Finally, the *O*‐*tert*‐butyl or *N*‐trityl protecting groups were deprotected from the HEA compounds **15**–**21** using trifluoroacetic acid (TFA) to provide the peptidomimetics **22**–**28** (Scheme 2).

The P_2_‐P_1_ analogues were synthesized by coupling commercially available amino acids of varying side chain (R^2^) functionality with HEA synthons **3** and **4** to provide intermediates **29**–**37**. Finally, the protecting group functionality was removed from analogues **29**–**33** using TFA to afford the peptidomimetics **38**–**42** (Scheme 3).

**Structure‐activity relationship**. The P_3_‐P_1_ and P_2_‐P_1_ peptidomimetic compounds synthesized were assessed in a PMX biochemical FRET assay to determine their activity. The results of the PMX biochemical assay showed that analogues varied in their ability to inhibit PMX based on functional changes to the P_1_, P_2_, P_3_ and N‐terminal positions (Table 1). P_3_‐P_1_ analogues that possessed a P_1_ Phe (**22**, **24**, **26**–**28**) (IC_50_ <1 μM) were more potent than analogues with a Leu in this position (**23** and **25**) (IC_50_ >1 μM), indicating PMX prefers a P_1_ Phe, consistent with previously reported substrate cleavage data.^[28]^ This was supported by the sub‐micromolar activity observed with P_2_‐P_1_ analogues **38** and **34**–**42**. PMX activity of analogues **22** and **24** showed hydrophobic amino acids Phe and Leu were tolerated in the P_2_ position (IC_50_ 0.19 and 0.2 μM). Activity of analogues **26**–**28** (IC_50_ 0.01–0.58 μM) demonstrated that Ser was also accepted in the P_2_ position, and further supported by the activity of analogue **38** in Table 2. Analogue **26** (IC_50_ 0.01 μM) indicated that P_3_ Ile was preferred compared to Asn (**27**) or Ser (**28**) which exhibited lower potency (IC_50_ 0.58 μM for both), suggesting a hydrophobic P_3_ amino acid is preferred.

**Table 1:** Activity of P_3_‐P_1_ analogues.

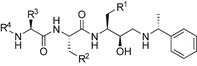							
Compd	R^4^	R^3^	R^2^	R^1^	PMX IC_50_ (SD) μM^[a]^	Pf 3D7 EC_50_ (SD) μM^[b]^	HepG2 CC_50_ (SD) μM^[c]^
**22**	Cbz	CH_2_OH	Ph	Ph	0.17 (0.02)	1.4 (0.35)	>40
**23**	Cbz	CH_2_OH	Ph	*i‐*Pr	3.6 (0.3)	6.0 (0.7)	30 (0.8)
**24**	Cbz	CH_2_OH	*i‐*Pr	Ph	0.20 (0.04)	1.6 (0.07)	>40
**25**	Cbz	CH_2_OH	*i‐*Pr	*i‐*Pr	3.5 (0.1)	>11	>40
**26**	Ac	*s‐*Bu	OH	Ph	0.01 (<0.01)	0.19 (0.01)	>40
**27**	Ac	CH_2_(CO)NH_2_	OH	Ph	0.58 (0.08)	8.3 (2.7)	>40
**28**	Ac	CH_2_OH	OH	Ph	0.58 (0.10)	>11	>40

[a] IC_50_ data represents the means and SD for 4 replicate fluorogenic substrate (Rh2 N) cleavage experiments. A ten‐point dilution series for each compound was incubated at 37 °C for 4 h with recombinant PMX. [b] EC_50_ data represents the mean and SD for 3 independent experiments measuring LDH activity of *P. falciparum* (Pf) 3D7 parasites following exposure to a ten‐point dilution series for each compound for 72 h at 37 °C. [c] CC_50_ data represents the mean and SD for 4 replicate HepG2 cell growth inhibition experiments following incubation at 37 °C with a ten‐point dilution series of each compound for 48 h. Cell Titer‐Glo was used to quantify cell viability.

**Table 2:** Activity of P_2_‐P_1_ analogues.

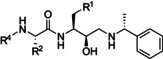						
Compd	R^4^	R^2^	R^1^	PMX IC_50_ (SD) μM^[a]^	Pf 3D7 EC_50_ (SD) μM^[b]^	HepG2 CC_50_ (SD) μM^[c]^
**34**	Cbz	*i‐*Pr	Ph	0.01 (0.01)	0.06 (0.02)	13
**35**	Cbz	*i‐*Bu	Ph	0.18 (0.02)	0.60 (0.02)	18
**36**	Cbz	CH_2_Ph	Ph	0.07 (<0.01)	0.64 (0.06)	>40
**37**	Cbz	*s*‐Bu	Ph	0.02 (<0.01)	0.15 (0.01)	>40
**38**	Cbz	CH_2_OH	Ph	0.06 (0.03)	0.09 (0.01)	>40
**39**	Ac	CH_2_OH	Ph	3.3 (0.6)	6.2 (0.2)	>40
**40**	Cbz	CH_2_OH ^[d]^	Ph	0.33 (0.04)	0.57 (<0.01)	>40
**41**	Cbz	CH_2_OH	*i*Pr	1.8 (0.02)	3.2 (0.73)	>40
**42**	Cbz	CH_2_(CH_3_)OH	Ph	0.16 (0.01)	0.26 (0.01)	>40
**2**	–	–	–	0.001 (<0.001)	0.001 (<0.001)	–

[a] IC_50_ data represents the means and SD for 4 replicate fluorogenic substrate (Rh2N) cleavage experiments. A ten‐point dilution series for each compound was incubated at 37 °C for 4 h with recombinant PMX. [b] EC_50_ data represents the mean and SD for 3 independent experiments measuring LDH activity of *P. falciparum* (Pf) 3D7 parasites following exposure to a ten‐point dilution series for each compound for 72 h at 37 °C. [c] CC_50_ data represents the mean and SD for 4 replicate HepG2 cell growth inhibition experiments following incubation at 37 °C with a ten‐point dilution series of each compound for 48 h. Cell Titer‐Glo was used to quantify cell viability. [d] D‐configured.

The P_2_ position was further explored in the P_2_‐P_1_ analogues while Phe was maintained in the P_1_ position (Table 2). This data showed that P_2_‐P_1_ analogues generally displayed potent activity against PMX. However, the activity of P_2_‐P_1_ analogues was seemingly lost when the N‐terminal cap R^4^ functionality was changed from Cbz (**38**) (IC_50_ 0.05 μM) to Ac (**39**) (IC_50_ 3.3 μM). The Cbz functionality of P_3_‐P_1_ analogues is likely to reside in the P_3_ position and suggests there is a hydrophobic interaction between the Cbz group and the S_3_ pocket of PMX that contributes to the observed biochemical activity. Consistent with the P_2_‐P_1_ analogues **23** and **25**, analogue **41** demonstrated that Leu in the P_1_ position was detrimental to PMX inhibitory activity (IC_50_ 1.8 μM).

The activity of the P_2_‐P_1_ analogues showed hydrophobic amino acids in the P_2_ position enhanced PMX inhibition. For example, analogues **34** and **37** with a P_2_ Val or Ile have IC_50_ values of 0.01 and 0.02 μM, respectively. Consistent with the activity of P_3_‐P_1_ analogues, P_2_‐P_1_ analogues **35** and **36** with Leu and Phe in the respective P_2_ positions also showed potent PMX inhibition (IC_50_ 0.18 and 0.07 μM, respectively). Analogues **38** and **42** with Ser and Thr in the P_2_ position also displayed potent activity (IC_50_ 0.05 and 0.16 μM). The inversion of side chain stereochemistry in the D‐configured P_2_ Ser (**40**) was 7‐fold less active (IC_50_ 0.33 μM) than the L‐configured Ser (**38**) counterpart, consistent with enzymes not tolerating substrates with d‐configured amino acids. Together this data implies PMX prefers amino acids with di‐substitution at the β‐carbon (Ile and Val) in the P_2_ position.

***P. falciparum*** **parasite activity**. The peptidomimetic analogues were evaluated in a *P. falciparum* asexual assay to demonstrate their concordance with the PMX biochemical data (Tables 1 and 2). The parasite activity of analogues with a P_1_ Phe (**22** and **24**) (EC_50_ 1.4 and 1.6 μM) were more potent than analogues with a Leu in this position (**23** and **25**) (EC_50_ 6 and >11 μM), which robustly correlates with the activity of these analogues in the PMX assay. Analogues **27** and **28** which possess Asn and Ser in the P_3_ position showed an approximately 10‐fold decrease in parasite activity (EC_50_ 6.7 and >11 μM) compared to PMX inhibition. Furthermore, the most potent P_2_‐P_1_ analogues **34** and **37** in the PMX assay were the most potent analogues in the parasite viability assay (EC_50_ 0.05 and 0.14 μM). Analogues **35** and **36** (P_2_ Leu and Phe) showed an approximately 6‐fold decrease in parasite growth inhibition (EC_50_ 0.6 and 0.65 μM) compared to PMX inhibition, whilst analogues **38** and **42**, with Ser and Thr in the P_2_ position, demonstrated a 2‐fold decrease in parasite activity. Overall, the *P. falciparum* viability data has a strong correlation with the PMX inhibitory activity (Figure S5), suggesting parasite death is a result of PMX inhibition.

**Human cell cytotoxicity**. The cytotoxicity of analogues was evaluated in a human hepatocyte (HepG2) growth inhibition assay (Table 1 and 2). Most of the peptidomimetic analogues did not affect cell viability at the highest concentration of 40 μM. The exceptions were analogues **23**, **34** and **35** that exhibited modest cytotoxicity with CC_50_ values of 30, 13 and 18 μM respectively. Although HepG2 activity was detected these analogues showed >200‐fold selectivity compared to the parasite activity. Collectively, this data suggests that the peptidomimetic compounds were not exhibiting off‐target toxicity in human HepG2 cells.

**Plasmepsin IX and V, and human aspartyl protease activity**. A representative selection of the peptidomimetics (**26**, **34**, **37** and **38**) were assessed for selectivity against PMIX, PMV and the human aspartyl proteases renin, BACE‐1 and cathepsin D in fluorogenic biochemical assays (Table 3). The data showed analogues **26**, **34**, **37** and **38** displayed weak activity against PMIX (IC_50_ 2.7–41 μM). This result is complexing because the substrate consensus sequence of PMIX (SFLE) is almost identical to the consensus sequence of PMX (SF*h*E), although PMIX does not cleave the same repertoire of substrates as PMX.[^27^, ^28^] The binding pockets PMX and PMIX are highly homologous, although there are subtle differences in amino acid composition that may contribute to selectivity observed with the peptidomimetic compounds. All four analogues were inactive against PMV (IC_50_ >90 μM). This result is expected as none of the peptidomimetics **26**, **34**, **37** and **38** resemble the RxL PEXEL sequence required for binding to PMV,^[21]^ and implied that PMV inhibition was not contributing to the parasite activity of analogues (Tables 1 and 2). All the analogues were inactive against renin, cathepsin D and BACE‐1 at the highest concentration tested (11 μM) except for analogue **26** which exhibited a modest IC_50_ of 5.5 μM against BACE‐1 and analogue **37** which showed potent inhibition (IC_50_ 0.20 μM) of cathepsin D (Table 3). The selectivity of the peptidomimetics **26**, **34**, **37** and **38** observed against human aspartyl proteases is attributed to differences in the substrate preferences between the human aspartyl protease and PMX. Overall, the analogues designed show potent inhibition of PMX with high selectivity against PMIX, PMV and human aspartyl proteases.

**Table 3:** Protease activity of representative analogues.^[a]^

Compd	PMX IC_50_ (SD) μM	PMIX IC_50_ (SD) μM	PMV IC_50_ (SD) μM	cathepsin D IC_50_ (SD) μM	BACE‐1 IC_50_ (SD) μM	renin IC_50_ (SD) μM
26	0.01 (<0.01)	2.7 (0.2)	>90	>11	2.9 (0.1)	>11
34	0.01 (0.01)	6.9 (1.7)	>90	>11	>11	>11
37	0.03 (0.01)	14.7 (0.6)	>90	0.20 (0.1)	>11	>11
38	0.06 (0.03)	41.0 (8.7)	>90	>11	>11	>11
2	0.001 (<0.001)	15.1 (4.4)	>90	>11	>11	>11

[a] IC_50_ data represents the mean and SD for 4 replicate FRET experiments. A ten‐point dilution series for each compound was incubated at 37 °C with fluorogenic substrates of each protease.

**Modelling of peptidomimetics in complex with PMX**. To rationalize the structural basis for the binding of the peptidomimetics to PMX, representative compounds **26**, **34** and **38** were modelled in complex with the X‐ray structure of PMX (PDB: 72BC).^[27]^ The X‐ray structure of the HEA compound, MR0C 803, bound to BACE‐1 (PDB: 2P83)^[38]^ acted as a template to overlay and orientate the peptidomimetics in the binding cleft of PMX before performing minimalization of the compounds.

**Figure 3:**
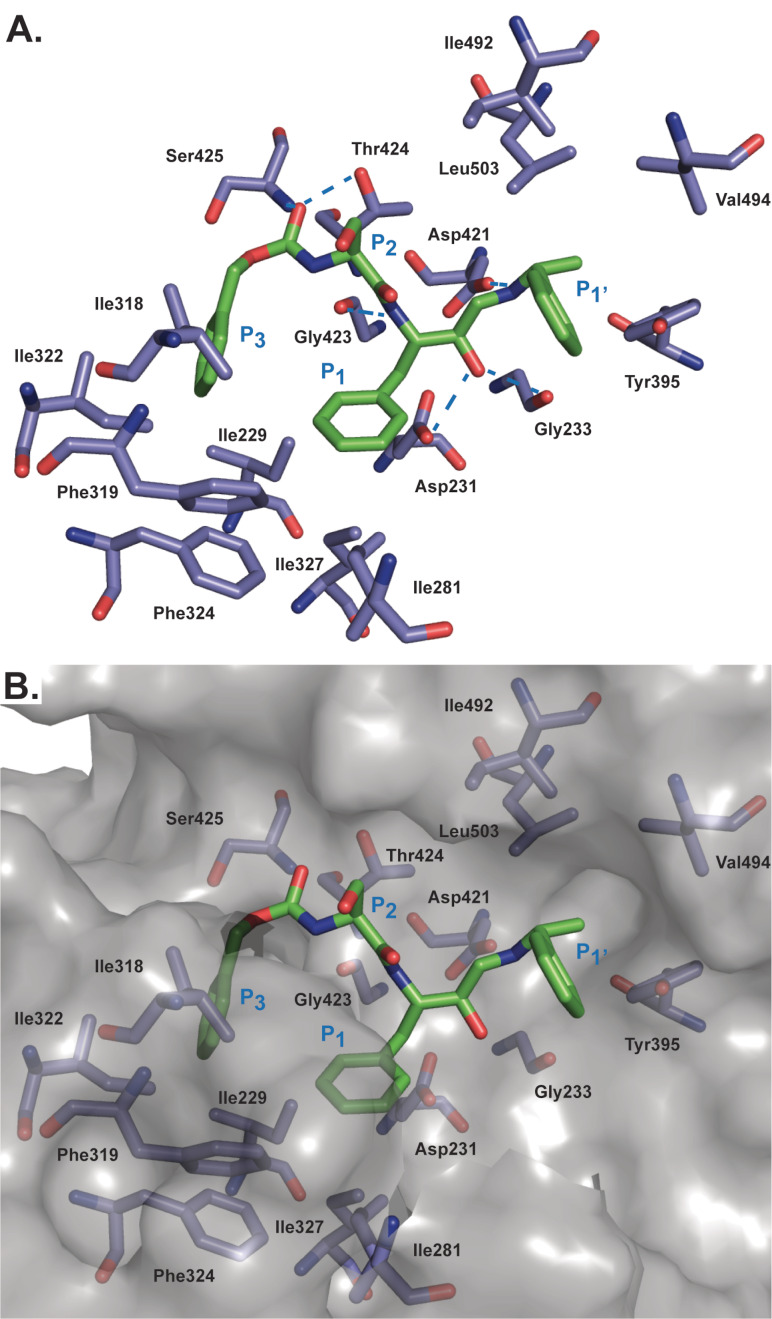
Model of the peptidomimetic **38** in complex with PMX. **A**. The model shows interactions of the P_3_ to P_1_’ motifs of compound **38** (green) with amino acids in the corresponding pocket of PMX (blue). Hydrogen bonds are shown with blue ‘dashed lines. **B**. Surface representation of the model showing substrate binding pockets of PMX accommodating the P_3_ to P_1_’ moieties of compound **38**. Positions of structural moieties of **38** are labelled relative to their respective binding pockets. PMX flap amino acids Ile274 to Ile281 were excluded for clarity, which includes the hydrogen bonds of Ser to Gly278 and Ser277 from PMX with the P_2_ Ser of **38**. The model was generated using the X‐ray structure of the HEA compound MR0C 803 bound to BACE‐1 (PDB: 2P83)^[38]^ as a template for positioning the peptidomimetic **38** before performing minimalization with the X‐ray structure of PMX (PDB: 72BC).^[27]^

The results of the modelling show that compounds **26**, **34** and **38** are positioned in the substrate binding cleft of PMX (Figures 3 and S6) and the catalytic aspartic acid dyad comprised of Asp266 and Asp457 have hydrogen bond interactions with the HEA hydroxyl and secondary amine groups of the peptidomimetics (Figure S6D). Additionally, all analogues formed a hydrogen bond between the HEA hydroxyl and Gly268. The P_1_’ α‐methylbenzyl moiety was positioned in the largely hydrophobic S_1_’ pocket consisting of Tyr395, Val494 and Leu503 (Figures 3 and S6). This structural information supports the preference for hydrophobic amino acids Leu, Val and Ile in the P_1_’ position of the SF*h*E substrate sequence of PMX (Figure S2). The S_1_ pocket of PMX comprised of hydrophobic amino acids Ile229, Ile 281, Phe319, Ile327 and Phe324 and Phe276 located on the flap on PMX, (Figure S6C) form hydrophobic interactions with the P_1_ Phe of the peptidomimetic analogues **26**, **34** and **38** (Figures 3 and S6). This data corroborates the preference for Phe in the P_1_ position of the SF*h*E substrate sequence and P_1_ hydrophobic groups of 49c and WM382 accommodated by the S_1_ pocket of PMX (Figure S7).

The S_2_ pocket of PMX is a large open cavity comprised of amino acids such as Thr424, Met490 and Ile492, and flap amino acids Gly277 and Ser 278 (Figures 3 and S6). The S_2_ pocket mostly consists of hydrophobic amino acids and accommodates hydrophobic amino acid side chains such as the Val in the P_2_ position of analogue **34**. Modelling of compounds **26** and **38** shows the P_2_ Ser forming hydrogen bond interactions with the flap amino acids Gly277 and Ser 278 (Figure S6C). The interactions predicted by the modelling reflects the preference of PMX for Ser in the P_2_ of position of the SF*h*E substrate sequence (Figure S2).

The S_3_ pocket of PMX is lined with hydrophobic amino acids, Ile229, Ile318 and Ile324 (Figures 3 and S6). Modelling shows the hydrophobic S_3_ pocket of PMX accommodates the Cbz functionality from analogues **34** and **38** and the P_3_ Ile of compound **26**. Additionally, the carbonyl group of the Cbz functionality of **34** and **38** and the carboxamide of **26** also form a hydrogen bond interaction with Thr424. This data provides a rationale for the potent PMX activity observed with the analogues **26**, **34** and **38**. Notably, the X‐ray structure of WM382 (**1**) in complex with PMX shows a chromane group positioned in the S_3_ pocket of PMX, highlighting the flexibility of the P_3_ pocket in accommodating a large variety of functionality (Figure S7), and further corroborates the consensus substrate sequence data illustrating a variety of amino acids are tolerated in the P_3_ position (Figure S2). Overall, the modelling provided structural insight into the observed peptidomimetic SAR and supported the PMX substrate consensus data.

**Asexual stage of arrest phenotyping**. Inhibition of PMX prevents parasites from egressing from the host erythrocyte and arrests asexual stage parasites at the late schizont stage. To demonstrate the representative PMX peptidomimetic **38** displayed a similar asexual stage killing profile to the PMX inhibitor 49c (**2**), we evaluated **38** in an asexual stage of arrest assay. In this assay, highly synchronized asexual ring stage parasites were treated with ten times the EC_50_ of 38 and 49c (**2**) and the DMSO vehicle control. The parasite stage of arrest was analyzed by microscopy of Giemsa‐stained blood smears, while parasitemia was quantified by flow cytometry at 12, 24, 36 and 48 hours.

**Figure 4:**
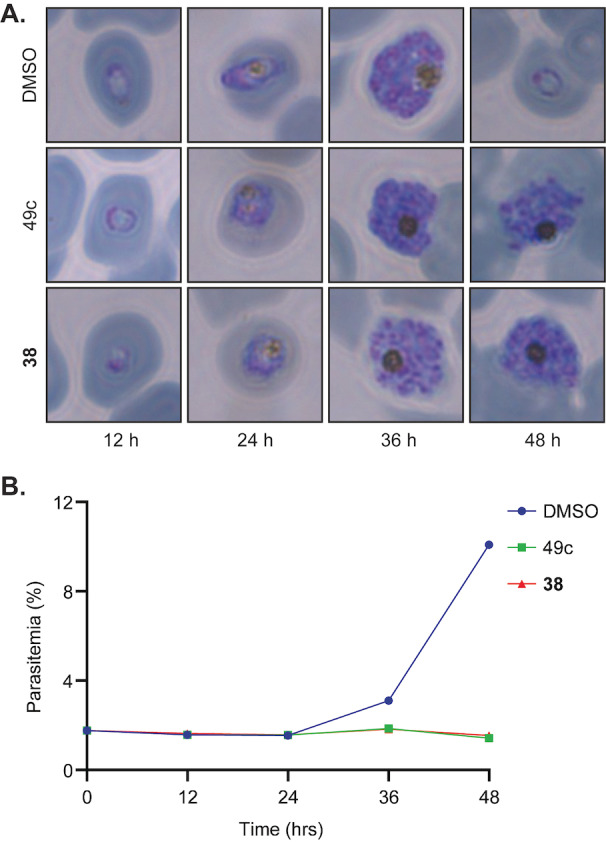
Analogue **38** blocks merozoite egress. **A**. Parasite morphology as determined by Giemsa‐stained blood smears demonstrated that 10×EC_50_ of **38** stalls merozoite egress, similarly to the control 49c (**2**). **B**. Flow cytometry of SYBR green‐stained infected RBCs determined the inhibition of merozoite egress with **38** and 49c (**2**) and correlated with the failure of merozoites to proliferate and establish RBC infection in the following cycle. Parasites were treated with 900 nM, 6 nM or 0.02 % of 38, 49c (**2**) or DMSO, respectively. Data points represent the mean of three technical replicates analyzed via flow cytometry.

The results show that **38** arrests asexual parasites at the schizont stage while DMSO treated parasites transition to the ring stage in the second asexual stage cycle at 48 h (Figures 4A and S8). Supporting this data, parasites treated with **38** were unable to establish infection beyond 36 h of the asexual stage (Figure 4B) The phenotype observed by compound **38** was consistent with the data shown with the PMX inhibitor 49c (**2**) (Figure 4 and S8), and WM‐382 (**1**) previously described, in preventing merozoite egress from the schizont and the host erythrocyte.[^26^, ^28^]

**PMX and PMIX ligand cleavage in** ***P. falciparum*** **parasites**. It was next determined whether **38** could inhibit PMX and PMIX processing of known invasion and egress ligands in *P. falciparum* parasites. SERA5 and ASP have been demonstrated as substrates of PMX and PMIX, respectively, and were chosen as ligands to measure the activity of these two proteases.[^26^, ^28^] SERA5 is not a direct substrate of PMX, but is processed by SUB1 which itself is activated by PMX. The degree of downstream processing of SERA5 by SUB1 is indicative of PMX activity.

In this experiment, synchronized late trophozoite/early schizont stage parasites, expressing HA‐tagged ASP, were incubated with **38** (10 μM), the positive control WM382 (**1**) (2.5 nM) (a dual potent inhibitor of PMIX and PMX) and the DMSO vehicle control. Parasites from each treatment condition were then lysed and the processing of the known PMX and PMIX substrates were detected by western blot as a corollary of protease activity.

**Figure 5:**
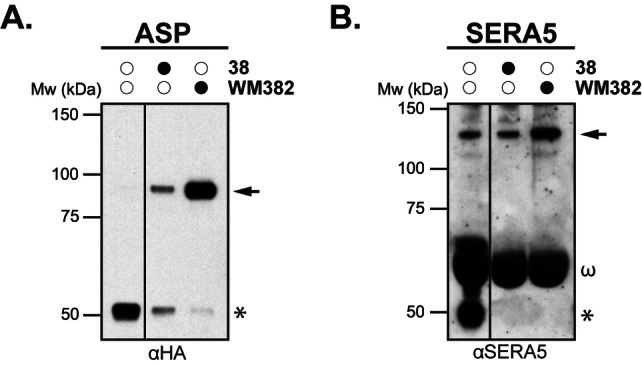
Evaluation of PMX and PMIX activity on processing of ASP and SERA5 respectively in *P. falciparum* parasites by peptidomimetic **38**. **A**. Evaluation of ASP processing by PMIX following treatment with the DMSO control, **38** (10 μM) and WM382 (**1**) (2.5 nM). **B**. Evaluation of SERA5 processing by SUB1, indicative of upstream activity by PMX, following treatment with the DMSO control, **38** (10 μM) and WM382 (**1**)^[28]^ (2.5 nM). The Western blot shown is after a long exposure. Arrows denote the unprocessed form of the respective substrates and asterisks signify the fully processed form. The band at 66 kDa indicated by ω is albumin protein non‐specifically detected by the SERA5 antibody.

PMIX processing of the ASP protein was detected through blotting with an anti‐HA antibody. The DMSO control showed full processing of ASP to the p47 form, indicated by the asterisk (Figure 5A). WM382 (**1**) treated parasites showed inhibition of ASP processing and an accumulation of the p87 form indicated by the arrow, whilst **38** showed partial inhibition of PMIX processing at 10 μM. Equivalent amounts of processed and unprocessed ASP were observed, indicating that **38** modestly inhibits PMIX.

PMX processing activity was assessed by blotting with an anti‐SERA5 antibody. The DMSO control sample showed processing of full‐length SERA5 to the p50 form, indicated by the asterisk (Figure 5B). WM382 (**1**) treated parasites showed complete inhibition of SERA5 processing and an accumulation of the 120 kDa full length protein, indicated by the arrow. Compound 38 similarly showed complete inhibition of SERA5 processing indicated by the absence of the processed p50 band. This lack of SERA5 processing indicates that SUB1 has not been processed and activated because PMX is inhibited. This data demonstrates that **38** can engage and inhibit both PMX and PMIX in parasites. The data also suggests **38** inhibits PMX more potently than PMIX, which is consistent with the biochemical inhibition PMX and PMIX by **38** (Table 3).

## Conclusion

In the present study, peptidomimetic compounds incorporating the HEA transition state mimetic were designed to mimic the P_3_‐P_1_ amino acids of PMX substrate binding sequence. The results from the SAR and the structural modelling on the peptidomimetics suggests PMX prefers a Phe in the P_1_ position, a di‐substituted β‐carbon amino acid in the P_2_ position, although Ser is also tolerated in the P_2_ position, and a hydrophobic amino acid in the P_3_ position. These findings mirror the PMX consensus sequence SF*h*E formed from PMX substrate cleavage experiments^[28]^ and proteome searches (Figure S2 and Table S1). The hydrophobic α‐methyl benzylamine motif, taken from the PMX inhibitor 49c (2), that occupies the P_1_’ position is also fitting with the PMX consensus sequence.

The peptidomimetics **26**, **34**, **37** and **38** that most closely mimicked the PMX substrate sequence potently inhibit PMX. These peptidomimetics are highly selective for PMX compared to PMIX, PMV and human aspartyl proteases. Weak inhibition of PMIX was unexpected because of the high homology and the similarities of the consensus sequence between these aspartyl proteases, although 49c (**2**) also displays the same selectivity profile, suggesting the P_1_’ α‐methyl benzylamine motif contributes to selectivity. Potent dual inhibition of PMX and PMIX is plausible and is reported with the antimalarial tool compound WM382 (1).[^27^, ^28^] This is an attractive and adventitious attribute in curbing the onset of resistance, further supporting PMX as an attractive antimalarial target.

Peptidomimetics **26**, **34**, **37** and **38** potently kill *P. falciparum* parasites and this activity is robustly correlated with PMX biochemical inhibition. To provide evidence that the parasite activity of **38** was a result of PMX inhibition, **38** was shown to block the processing of SERA5 indirectly via blocking the maturation of SUB1. Inhibition of ASP processing by **38** was detected at 10 μM, although this was incomplete, implying that **38** was killing the parasite via inhibition of PMX. Furthermore, compound 38 was shown to block parasite development at the schizont stage of asexual parasites which is consistent with the phenotype of reported PMX inhibitors **1** and **2**[^26^, ^28^] and the role of PMX in processing ligands essential for merozoite egress from the schizont and the host erythrocyte.

The peptidomimetic tool compounds described here have limitations compared to the known drug‐like inhibitors WM382 (**1**) and 49c (**2**). The peptide‐like nature of the peptidomimetic compounds limits their membrane permeability and *in vivo* absorption, and in addition metabolism of peptide‐like compounds is often challenging to correct which restricts their use in *in vivo* models and their development as an antimalarial therapeutic. Overall, mimicry of the PMX P_3_‐P_1_ substrate sequence has provided a conduit to inhibit PMX and kill *P. falciparum* parasites. has assisted in further understanding the PMX substrate specificity and furnished a template to assist in the future design of PMX targeted antimalarials.

## Experimental Section

**General Chemistry Methods**. All solvents were purchased commercially and used without further purification. Purification of compounds was performed by trituration with a specified solvent, or by CombiFlash Rf purification system with details as specified. LCMS were recorded on an Agilent LC‐ES/MS system composed of an Agilent G6120B Mass Detector, 1260 Infinity G1312B Binary pump, 1260 Infinity G1367E HiPALS autosampler and 1260 Infinity G4212B Diode Array Detector MS using an Orbitrap LTQ mass spectrometer. Conditions for LCMS were as follows, column: Poroshell 120 EC‐C18, 2.1×50 mm 2.7 Micron at 20 °C, injection volume of 2 μL, with a gradient of 5–100 % B over 5 min (solvent A: water 0.1 % formic acid; solvent B: ACN 0.1 % formic acid), with a flow rate of 0.8 mL/min and detection at 214 or 254 nm over 4.5 min. Nuclear magnetic resonance (NMR) spectra were recorded on either a Bruker Avance NEO (300 MHz), a Agilent MR400 (400 MHz), a Varian INOVA 600 (600 MHz) or a Bruker Avance III (600 MHz). NMR solvents are specified in compound synthesis methods. Chemical shifts are reported as δ values in parts per million (ppm), referenced to the specified solvent peak (7.26 ppm for CDCl_3_, 3.31 ppm for MeOD and 2.50 ppm for DMSO‐*d*_6_) and coupling constants reported in Hz. Unless otherwise noted, all compounds were found to be of >95 % purity.

(2*R*,3*S*)‐1,2‐Epoxy‐3‐(Boc‐amino)‐4‐isopropylbutane was synthesized as previously described.^[39]^ All other building blocks and reagents were purchased commercially. All amino acid derivatives used were L‐ configured unless otherwise specified. In the following experimental section the compound nomenclature Phe‐HEA‐Bn(CH_3_) refers to (2*R*,3*S*)‐3‐amino‐4‐phenyl‐1‐((*R*)‐1‐phenylethylamino)butan‐2‐ol and Leu‐HEA‐Bn(CH_3_) refers to (2*R*,3*S*)‐3‐amino‐5‐methyl‐1‐((*R*)‐1‐phenylethylamino)hexan‐2‐ol.

**General Procedure A: Cbz‐Ser(O*t*Bu)‐Leu‐OMe (6)**. A mixture of Cbz‐Ser(O*t*Bu)‐OH (152 mg, 0.51 mmol), HCl⋅NH_2_‐Leu‐OMe (112 mg, 0.62 mmol), HBTU (242 mg, 0.64 mmol) and DIPEA (400 μL, 2.31 mmol) in DMF (1.4 mL) was allowed to stir for 18 h at 20 °C. Citric acid solution (10 %) (10 mL) was added and the solution extracted with EtOAc (3×10 mL). The combined organic layer was then washed with NaHCO_3_ (30 mL) and brine (30 mL), dried with MgSO_4_ and concentrated to dryness *in vacuo*. The resulting residue was purified using column chromatography eluting with 0–6 % MeOH/DCM to give the title compound **6** (225 mg, 94 %) as a white solid. ^1^H NMR (400 MHz, CDCl_3_): δ 7.41–7.17 (m, 5H), 5.72 (s, *J*=6.5 Hz, 1H), 5.12 (s, 2H), 4.62 (td, *J*=8.7, 4.4 Hz, 1H), 4.25 (s, 1H), 3.85 (s, 1H), 3.72 (s, 3H), 3.39 (t, *J*=8.2 Hz, 1H), 1.70–1.60 (m, 2H), 1.59–1.51 (m, 2H), 1.21 (s, 9H), 0.93 (d, *J*=2.3 Hz, 3H), 0.92 (d, *J*=2.5 Hz, 3H). LCMS: *t*_R_ 2.48 min, [M+H]^+^ 423 *m/z*.

**Cbz‐Ser(O*t*Bu)‐Phe‐OEt (5)**. General procedure A was followed using Cbz‐Ser(O*t*Bu)‐OH (203 mg, 0.69 mmol) and HCl⋅NH_2_‐Phe‐OEt (182 mg, 0.79 mmol) to obtain the title compound **5** as a white solid (283 mg, 87 %). ^1^H NMR (400 MHz, CDCl_3_): δ 7.38–7.09 (m, 10H), 5.68 (s, 1H), 5.11 (s, 2H), 4.88–4.82 (m, 2H), 4.23 (s, 1H), 4.14 (q, *J*=6.6 Hz, 2H), 3.82 (s, 1H), 3.37 (t, *J*=8.2 Hz, 1H), 3.10 (d, *J*=5.4 Hz, 2H), 1.22 (t, *J*=7.1 Hz, 3H), 1.15 (s, 9H). LCMS: *t*_R_ 2.63 min, [M+H]^+^ 471 *m/z*.

**Ac‐Ile‐Ser(O*t*Bu)‐OMe (7)**. General procedure A was followed using Ac‐Ile‐OH (403 mg, 2.32 mmol) and HCl⋅NH_2_‐Ser(O*t*Bu)‐OMe (498 mg, 2.35 mmol) to obtain the title compound **7** as a pale red solid (455 mg, 59 %). ^1^H NMR (400 MHz, MeOD): δ 4.60–4.56 (m, 1H), 4.30 (d, *J*=7.7 Hz, 1H), 3.81–3.76 (m, 1H), 3.72 (s, 3H), 3.64–3.59 (m, 1H), 1.99 (s, 3H), 1.89–1.80 (m, 1H), 1.60–1.51 (m, 1H), 1.25–1.20 (m, 2H), 1.17 (s, 9H), 0.96 (d, *J*=6.9 Hz, 3H), 0.92 (t, *J*=7.3 Hz, 3H). LCMS: *t*_R_ 1.45 min, [M+H]^+^ 331 *m/z*.

**Ac‐Asn‐Ser(O*t*Bu)‐OMe (8)**. General procedure A was followed using Ac‐Asn(*N*‐Trt)‐OH (43.5 mg, 0.10 mmol) and HCl⋅NH_2_‐Ser(O*t*Bu)‐OMe (25.3 mg, 0.12 mmol) to obtain the title compound **8** as a white solid (32.6 mg, 54 %). ^1^H NMR (400 MHz, MeOD): δ 7.27–7.20 (m, 15H), 4.55–4.49 (m, 2H), 3.83–3.77 (m, 2H), 3.64–3.56 (m, 2H), 2.01–2.00 (m, 1H), 1.99–1.97 (m, 3H), 1.96 (s, 2H), 1.16–1.13 (m, 9H). LCMS: *t*_R_ 2.37 min, [M+H]^+^ 574 *m/z*.

**Ac‐Ser(O*t*Bu)‐Ser(O*t*Bu)‐OMe (9)**. General procedure A was followed using Ac‐Ser(O*t*Bu)‐OH (450 mg, 2.22 mmol) and HCl⋅NH_2_‐Ser(O*t*Bu)‐OMe (476 mg, 2.25 mmol) to obtain the title compound **9** as a red oil (846 mg, 94 %). ^1^H NMR (400 MHz, CDCl_3_): δ 7.67 (d, *J*=8.6 Hz, 1H), 6.45 (d, *J*=5.9 Hz, 1H), 4.72–4.66 (m, 1H), 4.51–4.44 (m, 1H), 3.87–3.78 (m, 2H), 3.77–3.71 (m, 3H), 3.57–3.52 (m, 1H), 3.39–3.33 (m, 1H), 2.03 (s, 3H), 1.24 (d, *J*=8.6 Hz, 9H), 1.14 (s, 9H). LCMS: *t*_R_ 1.62 min, [M+H]^+^ 361 *m/z*.

**General Procedure B: Cbz‐Ser(O*t*Bu)‐Phe‐OH (10)**. Cbz‐Ser(O*t*Bu)‐Phe‐OEt (**5**) (141.9 mg, 0.30 mmol) and LiOH (55 mg, 1.31 mmol) were dissolved in a mixture of water (2 mL) and THF (0.5 mL) and allowed to stir for 2 h at 20 °C. Citric acid solution (10 %) was added to the reaction mixture and the solution extracted with EtOAc (3×20 mL). The organic layer was then washed with brine (1×20 mL), dried (MgSO_4_) and concentrated to dryness *in vacuo*. This residue was triturated in Et_2_O and collected to give the title compound **10** (76.4 mg, 57 %) as a yellow oil. ^1^H NMR (400 MHz, MeOD): δ 7.42–7.08 (m, 10H), 5.14–5.05 (m, 2H), 4.74–4.67 (m, 1H), 4.25–4.18 (m, 1H), 3.60–3.50 (m, 2H), 3.22–3.13 (m, 1H), 3.09–3.00 (m, 1H), 2.94–2.74 (m, 2H), 1.19–0.97 (m, 9H). LCMS: *t*_R_ 2.35 min, [M+H]^+^ 443 *m/z*.

**Cbz‐Ser(O*t*Bu)‐Leu‐OH (11)**. General procedure B was followed with Cbz‐Ser(O*t*Bu)‐Leu‐OMe (**6**) (623 mg, 1.48 mmol) to give the title compound **11** (423 mg, 84 %) as a white solid. ^1^H NMR (400 MHz, MeOD): δ 7.42–7.17 (m, 5H), 5.11 (s, 2H), 4.54–4.44 (m, 1H), 4.30–4.23 (m, 1H), 3.71–3.63 (m, 1H), 3.63–3.56 (m, 2H), 1.80–1.68 (m, 1H), 1.68–1.58 (m, 2H), 1.18 (s, 9H), 1.00–0.82 (m, 6H). LCMS: *t*_R_ 2.13 min, [M+H]^+^ 409 *m/z*.

**Ac‐Ile‐Ser(O*t*Bu)‐OH (12)**. General procedure B was followed with Ac‐Ile‐Ser(O*t*Bu)‐OMe (**7**) (421 mg, 1.17 mmol) to give the title compound **12** (184 mg, 46 %) as a white solid. ^1^H NMR (400 MHz, MeOD): δ 4.57–4.50 (m, 1H), 4.34–4.25 (m, 1H), 3.84–3.76 (m, 1H), 3.67–3.59 (m, 1H), 1.99 (s, 3H), 1.89–1.82 (m, 1H), 1.61–1.50 (m, 2H), 1.17 (s, 9H), 0.96 (d, *J*=6.8 Hz, 3H), 0.91 (t, *J*=7.4 Hz, 3H). LCMS: *t*_R_ 1.17 min, [M+H]^+^ 317 *m/z*.

**Ac‐Asn(*N‐*Trt)‐Ser(O*t*Bu)‐OH (13)**. General procedure B was followed with Ac‐Asn(*N‐*Trt)‐Ser(O*t*Bu)‐OMe (**8**) (32.6 mg, 0.09 mmol) to give the title compound **13** (17.2 mg, 55 %) as a white solid. LCMS: *t*_R_ 2.12 min, [M+H]^+^ 560 *m/z*.

**Ac‐Ser(O*t*Bu)‐Ser(O*t*Bu)‐OH (14)**. General procedure B was followed with Ac‐Ser(O*t*Bu)‐Ser(O*t*Bu)‐OMe (**9**) (846 mg, 2.35 mmol) to give the title compound **14** (197 mg, 23 %) as a pale orange solid. ^1^H NMR (400 MHz, MeOD) δ 4.57–4.49 (m, 1H), 4.10 (q, *J*=7.1 Hz, 1H), 3.87–3.81 (m, 1H), 3.67–3.57 (m, 4H), 2.15 (s, 1H), 2.05–1.98 (m, 3H), 1.24–1.20 (m, 9H), 1.20–1.15 (m, 9H). LCMS: *t*_R_ 1.33 min, [M+H]^+^ 347 *m/z*.

**General Procedure C**: **Boc‐Phe‐HEA‐Bn(CH_3_) (1)**. A mixture of (2*R*,3*S*)‐1,2‐epoxy‐3‐(Boc‐amino)‐4‐phenylbutane (102.3 mg, 0.388 mmol) and (*R*)‐(+)‐α‐methylbenzylamine (60 μL, 0.471 mmol) was dissolved in dry MeOH (1.5 mL) and the reaction solution stirred for 48 h at 65 °C. The solvent was evaporated off under reduced pressure and the resulting residue was purified using column chromatography eluting with 0–2 % MeOH/EtOAc to give the title compound **1** (123.6 mg, 89 %) as a white solid. ^1^H NMR (400 MHz, CDCl_3_): δ 7.45–7.05 (m, 10H), 4.63 (d, *J*=9.2 Hz, 1H), 3.84–3.75 (m, 1H), 3.75–3.63 (m, 2H), 3.46–3.36 (m, 1H), 2.98–2.85 (m, 1H), 2.82–2.71 (m, 1H), 2.60–2.47 (m, 2H), 1.37 (d, *J*=6.7 Hz, 3H), 1.34 (s, 9H). LCMS: *t*_R_ 1.52 min, [M+H]^+^ 385 *m/z*.

**Boc‐Leu‐HEA‐Bn(CH_3_) (2)**. General procedure C was followed using (2*R*,3*S*)‐1,2‐epoxy‐3‐(Boc‐amino)‐4‐isopropylbutane (548 mg, 2.39 mmol) and (*R*)‐(+)‐α‐methylbenzylamine (300 μL, 2.36 mmol) to give the title compound **2** (351 mg, 42 %) as a white solid. LCMS: *t*_R_ 1.33 min, [M+H]^+^ 351 *m/z*.

**General Procedure D: 2HCl⋅NH_2_‐Phe‐HEA‐Bn(CH_3_) (3)**. Boc‐Phe‐HEA‐Bn(CH_3_) (**1**) (103 mg, 0.268 mmol) was dissolved in 4 N HCl in dioxane (4 mL) and stirred at 20 °C for 30 mins. The reaction solution was concentrated to dryness *in vacuo*. The crude material was washed in MeOH and concentrated to dryness to give the title compound **3** as a pale yellow solid (76.9 mg, 89.5 %). ^1^H NMR (400 MHz, MeOD): δ 7.47 (s, 5H), 7.33–7.23 (m, 5H), 4.44 (q, *J*=6.8 Hz, 1H), 4.29–4.23 (m, 1H), 3.76–3.72 (m, 1H), 3.68–3.65 (m, 2H), 3.60–3.55 (m, 1H), 3.09–3.02 (m, 1H), 2.97–2.91 (m, 1H), 2.87–2.80 (m, 1H), 2.74–2.66 (m, 1H), 1.70 (d, *J*=6.8 Hz, 3H). LCMS: *t*_R_ 0.85 min, [M+H]^+^ 285 *m/z*.

**2HCl⋅NH_2_‐Leu‐HEA‐Bn(CH_3_) (4)**. General procedure D was followed with Boc‐Leu‐HEA‐Bn(CH_3_) (**2**) (326 mg, 0.93 mmol) to give the title compound **4** (271 mg, 90 %) as a white solid. ^1^H NMR (400 MHz, MeOD): δ 7.58–7.46 (m, 5H), 4.49 (q, *J*=7.2 Hz, 1H), 4.26–4.19 (m, 1H), 3.40–3.33 (m, 2H), 3.12–3.04 (m, 1H), 2.80–2.69 (m, 1H), 1.74–1.71 (m, 3H), 1.62–1.56 (m, 1H), 1.50–1.37 (m, 2H), 1.33–1.22 (m, 2H), 0.96 (d, *J*=6.7 Hz, 3H), 0.91 (d, *J*=6.6 Hz, 3H). LCMS: *t*_R_ 0.41 min, [M+H]^+^ 251 *m/z*.

**General Procedure E: Cbz‐Ser(O*t*Bu)‐Phe**‐**Phe‐HEA‐Bn(CH_3_) (15)**. A mixture of Cbz‐Ser(O*t*Bu)‐Phe‐OH (**10**) (20.6 mg, 0.05 mmol), 2HCl⋅NH_2_‐Phe‐HEA‐Bn(CH_3_) (24 mg, 0.08 mmol), HBTU (23.3 mg, 0.1.32 mmol) and DIPEA (40 μL, 0.23 mmol) in DMF (500 μL) was allowed to stir for 18 h at 20 °C. Saturated NaHCO_3_ solution (10 mL) was added and the solution extracted with EtOAc (3×10 mL). The combined organic layer was then washed with brine (30 mL), dried with MgSO_4_ and concentrated to dryness *in vacuo*. The resulting residue was purified using column chromatography eluting with 0–10 % MeOH/DCM to give the title compound **15** (17 mg, 94 %) as a white solid. LCMS: *t*_R_ 2.62 min, [M+H]^+^ 709 *m/z*.

**Cbz‐Ser(O*t*Bu)‐Phe**‐**Leu‐HEA‐Bn(CH_3_) (16)**. General procedure E was followed using Cbz‐Ser(O*t*Bu)‐Phe‐OH (**10**) (65.9 mg, 0.16 mmol) and 2HCl⋅NH_2_‐Leu‐HEA‐Bn(CH_3_) (**4**) (35.3 mg, 0.11 mmol) to give the title compound **16** (41.5 mg, 56 %) as a clear oil. ^1^H NMR (400 MHz, CDCl_3_): δ 7.40–7.33 (m, 15H), 7.10 (d, *J*=6.7 Hz, 2H), 6.80–6.71 (m, 1H), 6.58–6.51 (m, 1H), 5.68 (s, 1H), 5.61–5.56 (m, 1H), 5.13–5.09 (m, 2H), 5.09–5.01 (m, 2H), 4.92–4.88 (m, 1H), 4.88–4.85 (m, 1H), 4.51 (q, *J*=6.4 Hz, 1H), 4.22 (s, 1H), 4.16–4.07 (m, 1H), 4.05–3.96 (m, 2H), 3.91–3.85 (m, 1H), 3.10 (t, *J*=5.4 Hz, 2H), 1.55 (d, *J*=6.5 Hz, 3H), 1.15 (s, 9H), 0.84–0.80 (m, 6H). LCMS: *t*_R_ 2.08 min, [M+H]^+^ 675 *m/z*.

**Cbz‐Ser(O*t*Bu)‐Leu‐Phe‐HEA‐Bn(CH_3_) (17)**. General procedure E was followed using Cbz‐Ser(O*t*Bu)‐Leu‐OH (**11**) (88.6 mg, 0.217 mmol) and 2HCl⋅NH_2_‐Phe‐HEA‐Bn(CH_3_) (**3**) (77.6 mg, 0.217 mmol) to give the title compound **17** (39.4 mg, 27 %) as a yellow oil. ^1^H NMR (400 MHz, CDCl_3_): δ 7.47–7.09 (m, 15H), 6.78 (s, 1H), 5.78 (d, *J*=5.5 Hz, 1H), 5.17–5.05 (m, 2H), 4.27 (d, *J*=6.2 Hz, 1H), 4.09–4.01 (m, 2H), 3.86 (s, 1H), 3.74–3.64 (m, 2H), 3.22–3.11 (m, 2H), 2.79–2.67 (m, 2H), 1.71 (d, *J*=6.7 Hz, 3H), 1.51–1.41 (m, 4H), 1.37–1.28 (m, 2H), 1.16 (s, 9H), 0.84 (d, *J*=6.5 Hz, 3H), 0.79 (d, *J*=6.4 Hz, 3H). LCMS: *t*_R_ 2.15 min, [M+H]^+^ 675 *m/z*.

**Cbz‐Ser(O*t*Bu)‐Leu‐Leu‐HEA‐Bn(CH_3_) (18)**. General procedure E was followed using Cbz‐Ser(O*t*Bu)‐Leu‐OH (**11**) (100 mg, 0.25 mmol) and 2HCl⋅NH_2_‐Leu‐HEA‐Bn(CH_3_) (**4**) (37.1 mg, 0.12 mmol) to give the title compound **18** (47.4 mg, 64 %) as a clear oil. ^1^H NMR (400 MHz, CDCl_3_): δ 7.45–7.31 (m, 10H), 5.18–5.07 (m, 2H), 4.37–3.52 (m, 8H), 3.48 (q, *J*=7.0 Hz, 2H), 1.74–1.43 (m, 12H), 1.18 (s, 9H), 0.98–0.82 (m, 12H). LCMS: *t*_R_ 1.99 min, [M+H]^+^ 641 *m/z*.

**Ac‐Ile‐Ser(O*t*Bu)‐Phe‐HEA‐Bn(CH_3_) (19)**. General procedure E was followed using Ac‐Ile‐Ser(O*t*Bu)‐OH (**12**) (40.9 mg, 0.13 mmol) and 2HCl⋅NH_2_‐Phe‐HEA‐Bn(CH_3_) (**3**) (44.5 mg, 0.12 mmol) to give the title compound **19** (44.3 mg, 59 %) as a clear oil. ^1^H NMR (400 MHz, CDCl_3_): δ 9.10 (s, 1H), 7.85–7.79 (m, 1H), 7.79–7.71 (m, 1H), 7.63 (s, 1H), 7.53–7.36 (m, 10H), 6.80 (d, *J*=8.6 Hz, 1H), 6.23 (s, 1H), 4.11–3.97 (m, 4H), 3.84–3.69 (m, 1H), 3.65–3.50 (m, 4H), 3.38–3.26 (m, 2H), 2.73–2.64 (m, 3H), 2.64–2.53 (m, 4H), 1.16 (s, 9H), 0.99–0.84 (m, 6H). LCMS: *t*_R_ 1.56 min, [M+H]^+^ 583 *m/z*.

**Ac‐Asn(*N*‐Trt)‐Ser(O*t*Bu)‐Phe‐HEA‐Bn(CH_3_) (20)**. General procedure E was followed using Ac‐Asn(*N*‐Trt)‐Ser(O*t*Bu)‐OH (**13**) (17.2 mg, 0.03 mmol) and 2HCl⋅NH_2_‐Phe‐HEA‐Bn(CH_3_) (**4**) (25.9 mg, 0.07 mmol) to give the title compound **20** (11.5 mg, 45 %) as a white solid. ^1^H NMR (400 MHz, CDCl_3_): δ 7.42–7.08 (m, 25H), 5.24 (s, 1H), 4.63 (s, 1H), 4.39–4.31 (m, 2H), 4.30–4.24 (m, 1H), 4.24–4.20 (m, 1H), 4.20–4.14 (m, 1H), 4.01–3.91 (m, 2H), 3.81–3.75 (m, 2H), 3.75–3.70 (m, 2H), 3.68–3.61 (m, 2H), 3.52–3.48 (m, 1H), 3.48–3.43 (m, 1H), 3.33–3.28 (m, 1H), 2.00 (s, 3H), 1.54 (d, *J*=7.2 Hz, 3H), 1.02 (d, *J*=4.6 Hz, 9H). LCMS: *t*_R_ 2.24 min, [M+H]^+^ 827 *m/z*.

**Ac‐Ser(O*t*Bu)‐Ser(O*t*Bu)‐Phe‐HEA‐Bn(CH_3_) (21)**. General procedure E was followed using Ac‐Ser(O*t*Bu)‐Ser(O*t*Bu)‐OH (**14**) (81.5 mg, 0.24 mmol) and 2HCl⋅NH_2_‐Phe‐HEA‐Bn(CH_3_) (**4**) (84.2 mg, 0.24 mmol) to give the title compound **21** (44.8 mg, 65 %) as an oil. ^1^H NMR (400 MHz, CDCl_3_): δ 7.40–7.12 (m, 10H), 6.78 (d, *J*=8.6 Hz, 1H), 4.43–4.37 (m, 1H), 4.35–4.27 (m, 1H), 4.25–4.19 (m, 2H), 4.06–3.99 (m, 1H), 3.80–3.72 (m, 2H), 3.72–3.66 (m, 2H), 3.64–3.59 (m, 1H), 3.50–3.46 (m, 1H), 3.28–3.23 (m, 1H), 3.10–3.03 (m, 1H), 2.84–2.78 (m, 2H), 2.56–2.49 (m, 1H), 1.99 (s, 3H), 1.63 (d, *J*=6.7 Hz, 3H), 1.22 (s, 9H), 1.01 (s, 9H). LCMS: *t*_R_ 1.76 min, [M+H]^+^ 613 *m/z*.

**Cbz‐Ser(O*t*Bu)‐Phe‐HEA‐Bn(CH_3_) (29)**. General procedure E was followed using Cbz‐Ser(O*t*Bu)‐OH (41.8 mg, 0.14 mmol) and 2HCl⋅NH_2_‐Phe‐HEA‐Bn(CH_3_) (**4**) (30.0 mg, 0.09 mmol) to give the title compound **29** (64.9 mg, 72 %) as an oil. ^1^H NMR (400 MHz, DMSO‐*d*_6_): δ 9.16 (s, 1H), 8.72 (s, 1H), 7.88 (d, *J*=8.4 Hz, 1H), 7.51–7.11 (m, 15H), 5.08–4.99 (m, 2H), 4.31–4.23 (m, 1H), 3.98–3.85 (m, 2H), 3.83–3.71 (m, 2H), 2.98 (d, *J*=12.4 Hz, 2H), 2.94–2.88 (m, 1H), 2.72–2.61 (m, 2H), 1.57 (d, *J*=6.7 Hz, 3H), 1.05 (s, 9H). LCMS: *t*_R_ 2.13 min, [M+H]^+^ 562 *m/z*.

**Ac‐Ser(O*t*Bu)‐Phe‐HEA‐Bn(CH_3_) (30)**. General procedure E was followed using Ac‐Ser(O*t*Bu)‐OH (42.5 mg, 0.14 mmol) and 2HCl⋅NH_2_‐Phe‐HEA‐Bn(CH_3_) (**4**) (40.0 mg, 0.13 mmol) to give the title compound **30** (37.5 mg, 54 %) as a white solid. ^1^H NMR (400 MHz, MeOD): δ 7.32–7.14 (m, 10H), 4.23 (t, *J*=5.9 Hz, 1H), 3.98–3.92 (m, 2H), 3.77–3.70 (m, 2H), 3.70–3.63 (m, 2H), 3.62–3.57 (m, 1H), 3.36 (d, *J*=5.9 Hz, 2H), 3.11–3.03 (m, 2H), 1.94 (s, 3H), 1.54–1.49 (m, 2H), 1.49–1.46 (m, 2H), 1.12 (s, 9H). LCMS: *t*_R_ 1.45 min, [M+H]^+^ 470 *m/z*.

**Cbz‐D‐Ser(O*t*Bu)‐Phe‐HEA‐Bn(CH_3_) (31)**. General procedure E was followed using Cbz‐D‐Ser(O*t*Bu)‐OH (51.4 mg, 0.17 mmol) and 2HCl⋅NH_2_‐Phe‐HEA‐Bn(CH_3_) (**4**) (48.6 mg, 0.15 mmol) to give the title compound **31** (58.1 mg, 68 %) as an oil. ^1^H NMR (400 MHz, CDCl_3_): δ 7.39–7.09 (m, 15H), 6.94 (s, 1H), 5.45 (s, 1H), 5.10 (s, 2H), 4.19 (d, *J*=6.6 Hz, 1H), 4.14–4.01 (m, 2H), 3.79 (s, 1H), 3.77–3.67 (m, 2H), 3.55–3.49 (m, 1H), 3.27–3.21 (m, 1H), 3.18 (q, *J*=7.4 Hz, 1H), 3.08–2.98 (m, 2H), 1.65 (d, *J*=6.7 Hz, 3H), 1.06 (s, 9H). LCMS: *t*_R_ 1.80 min, [M+H]^+^ 562 *m/z*.

**Cbz‐Ser(O*t*Bu)‐Leu‐HEA‐Bn(CH_3_**) **(32)**. General procedure E was followed using Cbz‐Ser(O*t*Bu)‐OH (55.7 mg, 0.19 mmol) and 2HCl⋅NH_2_‐Leu‐HEA‐Bn(CH_3_) (**3**) (42.5 mg, 0.15 mmol) to give the title compound **32** (46.0 mg, 59 %) as an oil. ^1^H NMR (400 MHz, CDCl_3_): δ 7.41–7.32 (m, 8H), 6.67 (s, 1H), 5.59 (s, 1H), 5.17–5.05 (m, 2H), 4.22–4.09 (m, 2H), 3.93–3.81 (m, 1H), 3.80–3.73 (m, 1H), 3.72–3.60 (m, 1H), 3.53–3.48 (m, 1H), 3.37 (t, *J*=7.5 Hz, 1H), 3.18 (q, *J*=7.5 Hz, 1H), 3.06–2.95 (m, 2H), 2.63 (s, 1H), 1.67–1.61 (m, 2H), 1.49–1.43 (m, 2H), 1.15 (s, 9H), 0.92–0.79 (m, 6H). LCMS: *t*_R_ 1.80 min, [M+H]^+^ 528 *m/z*.

**Cbz‐Thr(O*t*Bu)‐Phe‐HEA‐Bn(CH_3_) (33)**. General procedure E was followed using Cbz‐Thr(O*t*Bu)‐OH (58.8 mg, 0.12 mmol) and 2HCl⋅NH_2_‐Phe‐HEA‐Bn(CH_3_) (**4**) (38.6 mg, 0.11 mmol) to give the title compound **33** (23 mg, 37 %) as an oil. ^1^H NMR (400 MHz, CDCl_3_): δ 7.40–7.11 (m, 15H), 6.93 (d, *J*=9.1 Hz, 1H), 5.82 (d, *J*=5.4 Hz, 1H), 5.16–5.01 (m, 2H), 4.20–4.03 (m, 2H), 3.95–3.90 (m, 1H), 3.76–3.68 (m, 1H), 3.54–3.45 (m, 1H), 2.96–2.86 (m, 2H), 2.81–2.71 (m, 1H), 2.63–2.47 (m, 2H), 1.38 (d, *J*=6.6 Hz, 3H), 1.12 (s, 9H), 0.85 (d, *J*=6.4 Hz, 3H). LCMS: *t*_R_ 2.08 min, [M+H]^+^ 576 *m/z*.

**Cbz‐Val‐Phe‐HEA‐Bn(CH_3_) (34)**. General procedure E was followed using Cbz‐Val‐OH (53.1 mg, 0.21 mmol) and 2HCl⋅NH_2_‐Phe‐HEA‐Bn(CH_3_) (**4**) (52.4 mg, 0.15 mmol) to give the title compound **34** (50.8 mg, 67 %) as a white solid. ^1^H NMR (400 MHz, CDCl_3_): δ 7.41–7.06 (m, 15H), 6.74 (d, *J*=8.7 Hz, 1H), 5.09–5.02 (m, 2H), 4.18–4.06 (m, 1H), 4.01–3.91 (m, 1H), 3.81–3.73 (m, 1H), 3.74–3.65 (m, 1H), 3.54–3.41 (m, 2H), 2.97–2.82 (m, 2H), 2.72–2.56 (m, 2H), 2.02–1.92 (m, 1H), 1.50 (d, *J*=6.7 Hz, 3H), 0.74 (d, *J*=6.7 Hz, 3H), 0.65 (d, *J*=6.8 Hz, 3H). ^13^C NMR (101 MHz, CDCl_3_): δ 172.5, 156.6, 137.3, 136.0, 129.1, 129.0, 128.9, 128.60, 128.56, 128.4, 128.2, 127.1, 126.6, 82.1, 70.0, 67.4, 61.3, 59.0, 53.5, 48.8, 36.5, 30.1, 21.9, 19.1, 17.4. LCMS: *t*_R_ 1.81 min, [M+H]^+^ 518 *m/z*.

**Cbz‐Leu‐Phe‐HEA‐Bn(CH_3_) (35)**. General procedure E was followed using Cbz‐Leu‐OH (78 mg, 0.29 mmol) and 2HCl⋅NH_2_‐Phe‐HEA‐Bn(CH_3_) (**4**) (53 mg, 0.15 mmol) to give the title compound **35** (53.5 mg, 68 %) as an oil. ^1^H NMR (400 MHz, CDCl_3_): δ 7.42–7.10 (m, 15H), 6.73 (d, 1H), 5.12–5.03 (m, 2H), 5.04–4.96 (m, 1H), 4.19 (s, 1H), 4.04 (s, 1H), 3.95–3.87 (m, 1H), 3.82 (s, 1H), 3.75–3.63 (m, 1H), 3.16 (q, *J*=7.4 Hz, 1H), 3.09–2.98 (m, 2H), 2.74–2.65 (m, 2H), 1.62 (d, *J*=6.7 Hz, 3H), 1.28–1.17 (m, 2H), 0.82 (d, *J*=6.5 Hz, 3H), 0.78 (d, *J*=6.5 Hz, 3H). ^13^C NMR (101 MHz, CDCl_3_): δ 174.5, 137.2, 136.0, 129.2, 129.1, 129.0, 128.57, 128.54, 128.3, 128.1, 127.5, 126.6, 69.6, 67.4, 59.4, 55.7, 54.5, 53.1, 48.9, 43.6, 36.2, 24.5, 22.7, 21.6, 20.1, 12.5. LCMS: *t*_R_ 1.88 min, [M+H]^+^ 532 *m/z*.

**Cbz‐Phe‐Phe‐HEA‐Bn(CH_3_) (36)**. General procedure E was followed using Cbz‐Phe‐OH (62.7 mg, 0.21 mmol) and 2HCl⋅NH_2_‐Phe‐HEA‐Bn(CH_3_) (**4**) (59.6 mg, 0.16 mmol) to give the title compound **36** (59 mg, 62 %) as a white solid. ^1^H NMR (400 MHz, CDCl_3_): δ 7.40–6.98 (m, 20H), 6.73 (s, 1H), 5.13 (s, 1H), 5.03 (s, 2H), 4.22 (q, *J*=7.1 Hz, 1H), 4.10–4.03 (m, 1H), 3.86–3.77 (m, 1H), 3.52–3.45 (m, 1H), 3.16 (q, *J*=7.4 Hz, 1H), 2.91–2.80 (m, 2H), 2.78–2.69 (m, 2H), 2.69–2.61 (m, 1H), 2.51–2.43 (m, 1H), 1.44–1.42 (m, 3H). ^13^C NMR (101 MHz, CDCl_3_): δ 171.7, 141.9, 137.2, 136.2, 136.0, 129.2, 129.1, 129.0, 128.9, 128.8, 128.6, 128.5, 128.3, 128.1, 127.2, 127.1, 126.6, 69.5, 67.2, 58.9, 56.7, 55.8, 53.6, 48.7, 38.0, 36.5, 22.6. LCMS: *t*_R_ 1.95 min, [M+H]^+^ 566 *m/z*.

**Cbz‐Ile‐Phe‐HEA‐Bn(CH_3_) (37)**. General procedure E was followed using Cbz‐Ile‐OH (51 mg, 0.19 mmol) and 2HCl⋅NH_2_‐Phe‐HEA‐Bn(CH_3_) (**4**) (51 mg, 0.14 mmol) to give the title compound **37** (51.8 mg, 68 %) as a white solid. ^1^H NMR (400 MHz, CDCl_3_): δ 7.47–7.09 (m, 15H), 6.66 (d, *J*=8.7 Hz, 1H), 5.09 (s, 2H), 5.05–5.00 (m, 1H), 4.19–4.12 (m, 1H), 4.11–4.05 (m, 1H), 3.84–3.73 (m, 2H), 3.09–2.95 (m, 2H), 2.73–2.59 (m, 2H), 1.72–1.65 (m, 1H), 1.61 (d, *J*=6.7 Hz, 3H), 1.43 (d, *J*=6.7 Hz, 1H), 1.07–0.96 (m, 1H), 0.86–0.78 (m, 1H), 0.78–0.69 (m, 3H), 0.62 (d, *J*=6.8 Hz, 3H). ^13^C NMR (101 MHz, CDCl_3_): δ 173.2, 137.2, 136.0, 129.2, 129.1, 129.0, 128.6, 128.4, 128.2, 127.4, 127.3, 126.7, 82.1, 69.8, 67.5, 62.7, 60.8, 59.4, 54.9, 53.3, 48.8, 41.2, 36.3, 36.3, 24.3, 20.5, 15.3, 11.3. LCMS: *t*_R_ 1.92 min, [M+H]^+^ 532 *m/z*.

**General Procedure F: Cbz‐Ser‐Phe**‐**Phe‐HEA‐Bn(CH_3_)⋅TFA (22)**. A mixture of Cbz‐Ser(O*t*Bu)‐Phe‐Phe‐HEA‐Bn(CH_3_) (**15**) (8.6 mg, 0.001 mmol) in TFA (0.5 mL) and DCM (0.5 mL) was allowed to stir for 4 h at 20 °C. The reaction mixture was concentrated to dryness *in vacuo*, washed with MeOH and concentrated to dryness. The resulting residue was triturated with Et_2_O and collected to give the title compound **22** (7.8 mg, 81 %) as an oil. ^1^H NMR (400 MHz, MeOD): δ 7.50–7.13 (m, 20H), 5.13–5.06 (m, 2H), 4.41–4.35 (m, 1H), 4.34–4.28 (m, 1H), 4.08 (t, *J*=6.0 Hz, 1H), 3.96–3.88 (m, 1H), 3.81–3.70 (m, 2H), 3.69–3.57 (m, 2H), 3.25–3.18 (m, 1H), 3.05–2.98 (m, 1H), 2.83–2.75 (m, 1H), 2.66–2.58 (m, 2H), 1.67 (d, *J*=6.8 Hz, 3H). ^13^C NMR (101 MHz, MeOD): δ 172.5, 172.1, 171.5, 160.7, 157.3, 137.9, 136.8, 135.9, 129.2, 129.1, 128.9, 128.8, 128.5, 128.1, 128.1, 127.8, 127.5, 127.4, 126.4, 126.2, 102.6, 69.2, 66.6, 61.6, 58.5, 56.9, 55.7, 53.9, 48.6, 36.2, 36.0, 18.3. LCMS: *t*_R_ 1.84 min, [M+H]^+^ 653 *m/z*.

**Cbz‐Ser‐Phe**‐**Leu‐HEA‐Bn(CH_3_)⋅TFA (23)**. General procedure F was followed with Cbz‐Ser(O*t*Bu)‐Phe‐Leu‐HEA‐Bn(CH_3_) (**16**) (41.5 mg, 0.62 mmol) to give the title compound **23** (27.3 mg, 56 %) as an oil. ^1^H NMR (400 MHz, MeOD): δ 7.51–7.20 (m, 15H), 5.12–5.06 (m, 2H), 4.69 (t, *J*=6.8 Hz, 1H), 4.53–4.48 (m, 1H), 4.37 (q, *J*=7.0 Hz, 1H), 4.23–4.18 (m, 1H), 4.04 (t, *J*=5.8 Hz, 1H), 3.69–3.65 (m, 2H), 3.17–3.08 (m, 2H), 3.08–3.00 (m, 2H), 2.63–2.55 (m, 1H), 1.67 (d, *J*=6.9 Hz, 3H), 1.50–1.45 (m, 1H), 1.41–1.34 (m, 1H), 1.33–1.27 (m, 1H), 0.92–0.77 (m, 6H). ^13^C NMR (101 MHz, MeOD): δ 136.8, 135.9, 129.3, 129.1, 128.9, 128.7, 128.3, 128.12, 128.08, 127.5, 126.6, 126.5, 69.6, 66.6, 66.4, 61.7, 61.4, 58.4, 55.1, 53.8, 51.3, 50.6, 48.6, 38.8, 37.5, 37.0, 36.0, 24.0, 22.7, 20.1, 18.2. LCMS: *t*_R_ 1.72 min, [M+H]^+^ 619 *m/z*.

**Cbz‐Ser‐Leu‐Phe‐HEA‐Bn(CH_3_)⋅TFA (24)**. General procedure F was followed with Cbz‐Ser(O*t*Bu)‐Leu‐Phe‐HEA‐Bn(CH_3_) (**17**) (24.2 mg, 0.04 mmol) to give the title compound **24** (24.5 mg, 93 %) as an oil. ^1^H NMR (400 MHz, MeOD): δ 7.55–7.08 (m, 15H), 5.19–5.06 (m, 2H), 4.47–4.37 (m, 1H), 4.14 (t, *J*=6.1 Hz, 1H), 4.09–4.02 (m, 1H), 3.94–3.83 (m, 2H), 3.79–3.71 (m, 1H), 3.70–3.60 (m, 1H), 3.28–3.19 (m, 1H), 3.11–3.03 (m, 1H), 2.71–2.63 (m, 1H), 2.62–2.52 (m, 1H), 1.69 (d, *J*=6.8 Hz, 3H), 1.55–1.46 (m, 1H), 1.39–1.33 (m, 2H), 1.24–1.17 (m, 2H), 0.83 (d, *J*=6.8 Hz, 3H), 0.78 (d, *J*=6.6 Hz, 3H). ^13^C NMR (101 MHz, MeOD): δ 173.7, 172.5, 157.2, 137.9, 135.9, 129.2, 129.1, 128.7, 128.3, 128.1, 128.0, 127.8, 127.5, 126.1, 69.4, 66.6, 61.7, 58.6, 56.8, 54.4, 53.6, 53.0, 48.8, 39.3, 36.1, 24.3 21.8, 20.2, 18.2, 17.3, 15.8. LCMS: *t*_R_ 1.78 min, [M+H]^+^ 619 *m/z*.

**Cbz‐Ser‐Leu‐Leu‐HEA‐Bn(CH_3_)⋅TFA (25)**. General procedure F was followed with Cbz‐Ser(O*t*Bu)‐Leu‐Leu‐HEA‐Bn(CH_3_) (**18**) (47.4 mg, 0.07 mmol) to give the title compound **25** (31.7 mg, 61 %) as an oil. ^1^H NMR (400 MHz, MeOD): δ 7.54–7.26 (m, 10H), 5.11 (t, *J*=7.6 Hz, 2H), 4.41 (d, *J*=6.9 Hz, 1H), 4.28–4.20 (m, 1H), 4.13–4.08 (m, 1H), 3.86–3.79 (m, 1H), 3.79–3.72 (m, 2H), 3.72–3.66 (m, 2H), 3.48 (q, *J*=7.0 Hz, 2H), 2.69–2.60 (m, 1H), 1.68 (d, *J*=6.9 Hz, 3H), 1.56 (d, *J*=7.1 Hz, 2H), 1.17 (t, *J*=7.0 Hz, 2H), 0.96–0.79 (m, 12H). ^13^C NMR (101 MHz, MeOD): δ 174.0, 172.4, 135.9, 129.2, 129.1, 128.1, 127.8, 127.5, 127.3, 69.7, 66.6, 61.6, 58.5, 57.1, 52.8, 52.6, 51.3, 50.4, 49.9, 48.6, 39.5, 38.9, 37.5, 24.5, 24.4, 22.7, 22.1, 20.1, 20.0, 18.3. LCMS: *t*_R_ 1.68 min, [M+H]^+^ 585 *m/z*.

**Ac‐Ile‐Ser‐Phe‐HEA‐Bn(CH_3_)⋅TFA (26)**. General procedure F was followed with Ac‐Ile‐Ser(O*t*Bu)‐Phe‐HEA‐Bn(CH_3_) (**19**) (44.3 mg, 0.08 mmol) to give the title compound **26** (24.1 mg, 50 %) as a yellow oil. ^1^H NMR (400 MHz, MeOD): δ 7.52–7.39 (m, 5H), 7.26–7.09 (m, 5H), 4.46–4.36 (m, 1H), 4.23–4.15 (m, 1H), 4.01–3.90 (m, 2H), 3.90–3.76 (m, 2H), 3.65 (d, *J*=10.8 Hz, 1H), 3.57 (d, *J*=5.2 Hz, 2H), 3.15 (d, *J*=11.8 Hz, 2H), 2.77–2.66 (m, 2H), 2.01 (s, 3H), 1.85–1.74 (m, 2H), 1.68 (d, *J*=6.0 Hz, 3H), 1.41–1.33 (m, 1H), 1.30–1.14 (m, 2H), 0.95–0.84 (m, 6H). ^13^C NMR (101 MHz, MeOD): δ 173.1, 172.6, 171.6, 137.8, 136.0, 129.2, 129.1, 128.8, 128.0, 127.4, 126.0, 69.1, 61.0, 59.4, 58.5, 55.8, 53.8, 48.8, 36.2, 35.6, 25.0, 21.2, 18.1, 14.5, 14.3, 10.2, 9.8. LCMS: *t*_R_ 1.26 min, [M+H]^+^ 527 *m/z*.

**Ac‐Asn‐Ser‐Phe‐HEA‐Bn(CH_3_)⋅TFA (27)**. General procedure F was followed with Ac‐Asn(*N*‐Trt)‐Ser(O*t*Bu)‐Phe‐HEA‐Bn(CH_3_) (**20**) (7.6 mg, 0.01 mmol) to give the title compound **27** (3.4 mg, 42 %) as a white solid. ^1^H NMR (400 MHz, MeOD): δ 7.53–7.10 (m, 10H), 4.67–4.57 (m, 1H), 4.40 (d, *J*=7.1 Hz, 1H), 4.15–4.07 (m, 1H), 3.97–3.90 (m, 1H), 3.89–3.82 (m, 1H), 3.70–3.58 (m, 2H), 3.58–3.46 (m, 2H), 3.19–3.09 (m, 2H), 2.78–2.70 (m, 2H), 2.70–2.62 (m, 2H), 1.99 (s, 3H), 1.69 (d, *J*=6.8 Hz, 3H). ^13^C NMR (151 MHz, MeOD): δ 138.1, 136.1, 129.3, 129.2, 129.1, 128.9, 128.4, 128.0, 81.8, 69.3, 62.0, 60.8, 58.6, 56.9, 55.7, 54.9, 54.5, 53.9, 50.9, 50.5, 40.7, 36.3, 35.6, 21.1, 18.1. LCMS: *t*_R_ 0.92 min, [M+H]^+^ 528 *m/z*.

**Ac‐Ser‐Ser‐Phe‐HEA‐Bn(CH_3_)⋅TFA (28)**. General procedure F was followed with Ac‐Ser(O*t*Bu)‐Ser(O*t*Bu)‐Phe‐HEA‐Bn(CH_3_) (**21**) (50 mg, 0.08 mmol) to give the title compound **28** (34.9 mg, 70 %) as an orange glass. ^1^H NMR (400 MHz, MeOD): δ 7.53–7.38 (m, 5H), 7.24–7.11 (m, 5H), 4.39 (t, *J*=6.9 Hz, 1H), 4.28 (t, *J*=5.8 Hz, 1H), 4.20–4.13 (m, 1H), 3.97–3.77 (m, 2H), 3.74–3.63 (m, 2H), 3.60–3.47 (m, 2H), 3.19–3.09 (m, 2H), 2.73–2.60 (m, 2H), 2.01 (s, 3H), 1.68 (m, 3H), 1.39–1.34 (m, 1H), 1.33–1.24 (m, 1H). ^13^C NMR (101 MHz, MeOD): δ 172.7, 172.0, 171.0, 137.8, 136.0, 129.2, 129.1, 128.9, 128.0, 127.4, 126.0, 108.8, 69.2, 61.3, 60.7, 58.6, 56.6, 56.2, 53.9, 48.8, 35.8, 35.5, 21.2, 18.2. LCMS: *t*_R_ 0.89 min, [M+H]^+^ 501 *m/z*.

**Cbz‐Ser‐Phe‐HEA‐Bn(CH_3_)⋅TFA (38)**. General procedure F was followed with Cbz‐Ser(O*t*Bu)‐Phe‐HEA‐Bn(CH_3_) (**29**) (9.6 mg, 0.002 mmol) to give the title compound **38** (9.0 mg, 85 %) as a oil. ^1^H NMR (400 MHz, MeOD): δ 7.58–7.04 (m, 15H), 5.08 (q, *J*=12.4 Hz, 2H), 4.36 (d, *J*=6.9 Hz, 1H), 3.99 (t, *J*=5.9 Hz, 1H), 3.93–3.77 (m, 2H), 3.59–3.44 (m, 2H), 3.22–3.08 (m, 2H), 2.76–2.63 (m, 2H), 1.68 (d, *J*=6.8 Hz, 3H), 1.42–1.34 (m, 2H). ^13^C NMR (101 MHz, MeOD): δ 172.0, 156.9, 136.6, 136.6, 135.9, 129.3, 129.1, 128.8, 128.1, 128.0, 127.7, 127.5 127.4, 126.1, 68.9, 66.4, 61.5, 58.4, 57.3, 54.2, 48.7, 35.4, 18.1, 17.3, 15.8. LCMS: *t*_R_ 1.46 min, [M+H]^+^ 506 *m/z*.

**Ac‐Ser‐Phe‐HEA‐Bn(CH_3_)⋅TFA (39)**. General procedure F was followed with Ac‐Ser(O*t*Bu)‐Phe‐HEA‐Bn(CH_3_) (**30**) (7.6 mg, 0.02 mmol) to give the title compound **39** (7.0 mg, 82 %) as an oil. ^1^H NMR (400 MHz, MeOD): δ 7.53–7.40 (m, 5H), 7.29–7.11 (m, 5H), 4.40 (d, *J*=6.8 Hz, 1H), 4.18 (t, *J*=5.8 Hz, 1H), 3.93–3.73 (m, 2H), 3.73–3.59 (m, 1H), 3.60–3.47 (m, 2H), 3.26–3.08 (m, 2H), 2.75–2.62 (m, 2H), 2.02–1.91 (m, 3H), 1.69 (d, *J*=6.7 Hz, 3H), 1.35–1.26 (m, 1H). ^13^C NMR (101 MHz, MeOD): δ 172.2, 171.5, 137.9, 136.0, 129.2, 129.1, 129.0, 128.8, 128.7 128.1, 128.0, 127.4, 126.1, 126.0, 69.0, 61.1, 61.2, 58.4, 55.9, 54.1, 48.7, 35.4, 29.3, 21.1, 18.2. LCMS: *t*_R_ 0.89 min, [M+H]^+^ 414 *m/z*.

**Cbz‐D‐Ser‐Phe‐HEA‐Bn(CH_3_)⋅TFA (40)**. General procedure F was followed with Cbz‐D‐Ser(O*t*Bu)‐Phe‐HEA‐Bn(CH_3_) (**31**) (43.8 mg, 0.08 mmol) to give the title compound **40** (26.2 mg, 54 %) as an oil. ^1^H NMR (400 MHz, MeOD): δ 7.45–7.15 (m, 15H), 5.11 (s, 2H), 4.35–4.26 (m, 1H), 3.98–3.88 (m, 2H), 3.82–3.70 (m, 2H), 3.25–3.17 (m, 2H), 3.17–3.11 (m, 1H), 2.67–2.60 (m, 1H), 1.66 (d, *J*=6.7 Hz, 3H). ^13^C NMR (101 MHz, MeOD): δ 172.2, 157.0, 137.8, 129.2, 129.0, 128.8, 128.1, 128.0, 127.7, 127.41, 127.35, 126.1, 69.2, 66.3, 61.9, 58.5, 57.5, 54.4, 53.7, 37.5, 35.7, 17.7, 17.3, 15.8. LCMS: *t*_R_ 1.47 min, [M+H]^+^ 506 *m/z*.

**Cbz‐Ser‐Leu‐HEA‐Bn(CH_3_)⋅TFA (41)**. General procedure F was followed with Cbz‐Ser(O*t*Bu)‐Leu‐HEA‐Bn(CH_3_) (**32**) (23.5 mg, 0.05 mmol) to give the title compound **41** (12.1 mg, 46 %) as an oil. ^1^H NMR (400 MHz, MeOD): δ 7.56–7.24 (m, 10H), 5.18–5.04 (m, 2H), 4.37 (s, 1H), 4.10 (s, 1H), 3.84–3.66 (m, 3H), 3.61 (s, 1H), 3.11–3.03 (m, 1H), 2.81 (s, 1H), 2.71 (s, 1H), 1.73–1.63 (m, 3H), 1.56–1.43 (m, 2H), 1.37 (s, 2H), 0.97–0.74 (m, 6H). ^13^C NMR (75 MHz, MeOD): δ, 136.7, 135.9, 129.3, 129.1, 128.2, 128.1, 127.7, 127.5, 127.5, 69.7, 67.4, 66.4, 61.7, 58.4, 57.2, 50.5, 38.7, 24.3, 24.2, 22.7, 22.7, 20.1, 20.0, 18.0, 15.0. LCMS: *t*_R_ 1.45 min, [M+H]^+^ 473 *m/z*.

**Cbz‐Thr‐Phe‐HEA‐Bn(CH_3_)⋅TFA (42)**. General procedure F was followed with Cbz‐Thr(O*t*Bu)‐Phe‐HEA‐Bn(CH_3_) (**33**) (17.8 mg, 0.03 mmol) to give the title compound **42** (9.1 mg, 46 %) as an oil. ^1^H NMR (400 MHz, MeOD): δ 7.52–7.07 (m, 15H), 5.11 (d, *J*=12.5 Hz, 1H), 5.05 (d, *J*=12.4 Hz, 1H), 4.37 (d, *J*=6.7 Hz, 1H), 4.09 (q, *J*=7.1 Hz, 1H), 3.92–3.86 (m, 1H), 3.81–3.76 (m, 2H), 3.19–3.11 (m, 1H), 3.11–3.02 (m, 1H), 2.74–2.63 (m, 2H), 1.67 (d, *J*=6.9 Hz, 3H), 1.23 (t, *J*=7.1 Hz, 1H), 0.91 (d, *J*=5.9 Hz, 3H). ^13^C NMR (151 MHz, MeOD): δ 172.2, 171.6, 137.9, 136.7, 136.1, 135.8, 129.3, 129.1, 128.8, 128.3, 128.1, 128.0, 127.7, 127.4, 126.1, 68.9, 67.0, 66.5, 61.9, 61.4, 58.4, 54.1, 35.4, 18.7, 17.9, 13.0. LCMS: *t*_R_ 1.55 min, [M+H]^+^ 520 *m/z*.

### Biology Experimental

**FRET Protease Assays**. FRET protease assays were performed as previously described.[^19^, ^20^, ^27^, ^28^] Briefly, ten‐point 1 : 3‐fold dilution series of the compounds were prepared in 384‐well black low volume assay plates (Greiner) using an Echo555 (Labcyte). 10 mM compound stocks were transferred into assay plates such that the starting concentration was 90 μM (Pv PMV assays), 11.25 μM (renin, cathepsin D and BACE‐1 assays) or 100 nM and 10 μM (Pf PMX and Pf PMIX assays). All wells were backfilled to 200 nL DMSO such that the DMSO concentration remained constant across the assay plates (1 % final). All compound potency assays were conducted in 20 μL total volume. For each assay, 10 μL of recombinant enzymes in respective assay buffers (Table S2) were dispensed into compound containing assay plates using a Multidrop‐Combi dispenser and allowed to incubate for 15 min. Recombinant Pf PMX, Pf PMIX and Pv PMV were expressed as previously described,[^23^, ^27^, ^28^] and recombinant human aspartyl proteases were purchased commercially. 10 μL of the fluorogenic peptide substrates incubated at 37 °C for the duration and conditions noted in Table S2. Samples were excited at 340 nm and fluorescence emission measured at 492 nm using an Envision fluorescence plate reader (Perkin‐Elmer). The 0 % inhibition control contained DMSO alone (1 % final) and the 100 % inhibition control was in the absence of enzyme. Half‐maximal inhibitory concentration (IC_50_) values were calculated by GraphPad Prism (8.0.2) software using a nonlinear regression four‐parameter fit analysis using a sigmoidal dose response (variable slope).

***P. falciparum*** **LDH Viability Assay**. *P. falciparum* LDH viability assays were performed as previously described.^[40]^ Briefly, a ten‐point 1 : 3‐fold dilution series of the compounds were pre‐dispensed in 384‐well assay plates (Grenier), and RPMI/AlbuMAX growth media inoculated with synchronized ring stage *P. falciparum* parasites. 10 mM compound stocks were transferred into the assay plates such that the starting concentration was 10 μM. Plates were incubated for 72 h and then frozen at −80 °C overnight. Lactate dehydrogenase (LDH) activity was quantified with APAD by measuring absorbance of NBT at 650 nm. Data were normalized to percent growth inhibition using the positive control of 0.1 % DMSO alone and the negative control of 100 nM chloroquine. Half‐maximal effective concentration (EC_50_) values were calculated by GraphPad Prism (8.0.2) software using a nonlinear regression four‐parameter fit analysis using a sigmoidal dose response (variable slope).

**HepG2 Cytotoxicity Assay**. HepG2 cytotoxicity assays were performed as previously described.^[41]^ Briefly, human hepatocyte HepG2 cells were cultured in DMEM supplemented with 10 % FCS at 37 °C and 5 % CO_2_ in a humidified incubator. Cells were seeded at 1000 cells/well in 50 μL in 384‐well plates (Grenier) and compounds incubated in ten‐point 1 : 2‐fold dilution series for 48 h. Cytotoxicity was determined using Cell Titer‐Glo (Promega), normalized to DMSO alone controls (100 % viability) and control compound Bortezomib (0 % viability at 10 μM). CC_50_ values were calculated by GraphPad Prism (8.0.2) software using a nonlinear regression four‐parameter fit analysis with the sigmoidal dose response (variable slope).

**Molecular Modelling Studies**. CLC Drug Discovery Workbench software (version 2.5.1) was used to model compound **38** to the X‐ray structure of Pf PMX (PDB 72BC).^[27]^ The input ligands **26**, **34**, **38** and 49c were built using ligand designer and centralized to the binding region of PMX using a co‐crystal structure of the aspartyl protease inhibitor, MR0C 803, bound to BACE‐1 (PDB accession number 2P83).^[38]^ The binding conformation of the ligands were then minimalized. This method detects various flexible ligand conformations while holding the protein as a rigid structure during docking. The default number of iterations was set at 500. The ligand binding interactions of the resulting minimizations were observed using the CLC Drug Discovery visualization tool. The structural data was then exported into the PyMOL Molecular Graphics System (version 2.0) software for visualization.

**Stage Arrestment Assay**. As previously described,^[42]^ *P. falciparum* 3D7 parasites were synchronized using a 5 % D‐sorbitol solution prior to assay set‐up. Ring‐staged parasites were adjusted to a final parasitemia and haematocrit of 1 % and 2 %, respectively. Compounds were added to a final concentration of 10×EC_50_ which were replenished after 24 h. Plates were incubated at 37°C for 48 h and blood smears and 50 μL aliquots in triplicate were harvested every 12 h. Aliquots for flow cytometry analysis were fixed in 0.25 % glutaraldehyde for 30 min at room temperature before being washed in PBS and stored at 4 °C until completion of the assay. To determine parasitemia, fixed blood pellets were stained with 50 μL 2.5×SYBR Green (Invitrogen) for 30 min before being diluted with 180 μL of PBS. 100 000 cells for each sample were counted using an Attune Flow Cytometer (ThermoFisher Scientific). Blood smears were stained with Giemsa and morphology of parasites were examined using a Zeiss Axio Observer microscope.

***P. falciparum*** **ASP and SERA5 Processing Inhibition Assay**. *P. falciparum* substrate processing inhibition assays were performed as previously described.^[28]^ Synchronized ring stage cultures were treated with test compounds **38** (10 μM) and WM382 (2.5 nM)^[28]^ or DMSO as a vehicle control. The parasite cultures were grown to the schizont stage before passing over LD magnetic columns (Miltenyi Biotech) to remove uninfected erythrocytes. Parasites were eluted from columns with RPMI 1640 (in‐house) culture medium to which the appropriate inhibitor at the same concentration had been added. Eluted parasites were adjusted to 5×10^6^ schizonts/mL and 150 μL added per well of a 96‐well flat‐bottomed culture plate. The assay plates were further cultured for 16 h and a representative well from each condition smeared for Giemsa staining, to ensure either that rupture had occurred normally (control well) or that rupture had been blocked by **38** or WM382. Parasites from each condition were centrifuged at 10000×g for 10 min so that merozoite and supernatant fractions could be collected. Proteins from both fractions were extracted with reducing sample buffer and separated on 4–12 % or 3–8 % acrylamide gels (NuPAGE, Invitrogen). HA‐tagged apical sushi protein (ASP) was detected in the merozoite fraction with anti‐HA mAb and SERA5 was detected in the supernatant fraction with an anti‐SERA5 polyclonal Ab. Loading controls are unnecessary for these Western blots as tracks were loaded with proteins from equal cell numbers from both the merozoite and supernatant fractions.

## Conflict of interest

The authors declare no conflict of interest.

1

## Supporting information

As a service to our authors and readers, this journal provides supporting information supplied by the authors. Such materials are peer reviewed and may be re‐organized for online delivery, but are not copy‐edited or typeset. Technical support issues arising from supporting information (other than missing files) should be addressed to the authors.

Supporting Information

## Data Availability

The data that support the findings of this study are available from the corresponding author upon reasonable request.
