# Supplementary material for: Substrate Peptidomimetic Inhibitors of P. falciparum Plasmepsin X with Potent Antimalarial Activity
Source: ChemMedChem. 2022 Aug 18;17(18):e202200306. doi: 10.1002/cmdc.202200306 (PMC9804387; doi:10.1002/cmdc.202200306)
Supplement: Supplementary file 1 — Supporting Information [file CMDC-17-0-s001.pdf]

ChemMedChem

Supporting Information

Substrate Peptidomimetic Inhibitors of *P. falciparum* Plasmepsin X with Potent Antimalarial Activity

Lachlan W. Richardson, Trent D. Ashton, Madeline G. Dans, Nghi Nguyen, Paola Favuzza, Tony Triglia, Anthony N. Hodder, Anna Ngo, Kate E. Jarman, Alan F. Cowman, and Brad E. Sleebs*

SUPPORTING INFORMATION FOR

Index

Page	
S2	Figure S1. Overview of PMX and PMIX substrate processing.
S3	Table S1. PMX consensus sequence search.
S4	Figure S2. WebLogo plot of PMX substrate sequences.
S5	Figure S3. PMX dose-response curves of representative analogues.
S6	Figure S4. *P. falciparum* viability dose-response curves of representative analogues.
S7	Figure S5. Correlation plot of *P. falciparum* viability versus PMX activity.
S8	Figure S6. Model of peptidomimetics in complex with PMX.
S9	Figure S7. Model of peptidomimetics, WM382 and 49c in complex with PMX.
S10	Figure S8. Asexual stage of arrest microscopy images.
S11	Figure S9. *P. falciparum* processing western blots.
S12	Table S2. Protease FRET assay conditions.
S13	NMR or LC spectra of synthesized compounds.
S78	References.

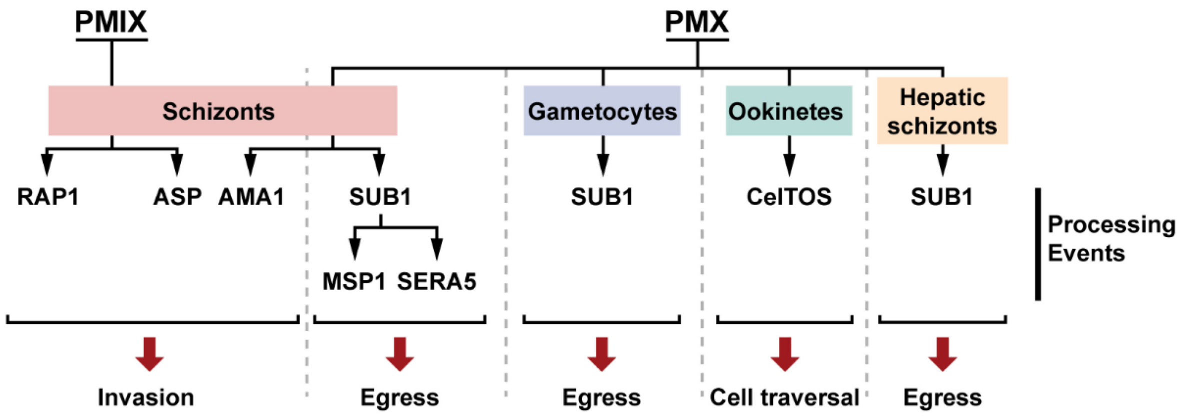

Figure S1. Overview of PMX and PMIX substrate processing. Summary of the activity of PMX and PMIX established in the literature, showing the processing of respective substrates at different stages of the parasite lifecycle.

Table S1. Consensus sequence search of the *P. falciparum* proteome identifies potential invasion and egress ligands of PMX. [a]

Search	Protein ID	Protein Name	Sequence [b]	Match range
/IE [c]	I1W9_PLAF7	ical sushi protein	SKLSFLEHKNHK	86
	I0V6_PLAF7	od stage antigen 41-3	TQSSFLEQEENE	0-283
	IBB9_PLAF7	I-anchored micronemal antigen	LLKSFIEEDEEQ	8-161
	IAS0_PLAF7	smepsin X	FRRSFIEKNLHI	1-304
	IC11_PLAF7	P protein, putative	EKESFIEDNIKK	7-680
	I3W9_PLAF7	s-related protein Rab-1A	LNISFIETSAKD	0-153
	IJ23_PLAF7	SA-like protein with PHIST and DnaJ domains	LFNSFLEIKKLD	5-488
	I4R5_PLAF7	optry neck protein 3	KEISFLERRETN	41-1844
	ILZ1_PLAF7	optry-associated protein 1	SESFLENKSSV	71
	U0K0_PLAF7	rozoite invasion-associated protein 1	QNNSFIEFMESA	6-459
	I0V0_PLAF7	tilisin-like protease 1	DVLSFLESKGNL	4-167
	I2F5_PLAF7	ulence-associated protein 1	LIYSFLESISYI	7-140
/ID [c]	IJ52_PLAF7	ffy binding-like merozoite surface protein	IKDNSFIDSKIEE	60
	IBE8_PLAF7	throcyte binding antigen-175	RILSFLDSRINN	6-139
			LKNSFLDYGHLA	8-241
	IM26_PLAF7	deosome associated protein with multiple membrane span	LASFLDTISAT	2-125
	I395_PLAF7	h molecular weight rhoptry protein 3	DAGSFLDFVDEP	62
	P3_PLAF7	ticulocyte-binding protein 3 precursor	ISQSFLDSMKLN	62-2165
	IBN1_PLAF7	optry-associated leucine zipper-like protein 1	VTHSFLDMSKAA	7-390
	A143ZXM2_PLAF7	izont egress antigen-1	EIKSFLDKLKSD	2-935
ILE	7302_PLAF7	5 interacting protein	NNISMLEIQNEI	8-531
	I0V0_PLAF7	tilisin-like protease 1	DYKSMLEVENDA	2-245
	I2D1_PLAF7	face-associated interspersed protein 1.3 [d]	YMSMLEEYVKE	5-638
VE	6NM5_PLAF7	throcyte binding antigen-140	LINSFVENKSVK	75
	IE80_PLAF7	deosome associated protein with multiple membrane span	ATSFVEVLALT	7-210
/IQ [c]	IIV3_PLAF7	cium/calmodulin-dependent protein kinase, putative	INKSSFIQQQHKM	43-1046
			RSYSFIQQNVKE	09-1412
	7267_PLAF7	cumsporozoite-and TRAP-related protein	TNKSFLQVQHTV	58
	6279_PLAF7	toadherence linked asexual protein 2 [d]	HVNSFLQLDFFH	1-724
			DTSFLQTVYLL	6-929
	IE80_PLAF7	deosome associated protein with multiple membrane span	LIMSFLQLTEDD	98
	IBB9_PLAF7	I-anchored micronemal antigen	GATSFIQSREVK	5-348
	I1X6_PLAF7	er specific protein 2, putative	TPSFIQNDIFL	77-1280
	IIC7_PLAF7	P protein, putative	EQRSFLQYCSQD	8-491
	IKU2_PLAF7	P protein, putative	LSNSFIQLKDEI	75-1578
	P2B_PLAF7	ticulocyte binding protein 2 homolog b	SFHSFIQEGKEE	57
	P2A_PLAF7	ticulocyte-binding protein 2 homolog a	SFHSFIQEGKEE	9-132
	I4R5_PLAF7	optry neck protein 3	VKCSFIQYPFNL	21-1624
			IPSPSFIQIDKVN	84-1787
	6229_PLAF7	optry neck protein 6	THTSFLQNCTIN	90
	H4M0_PLAF7	optry-associated membrane antigen	YEESFLQNDEKK	5-478
	IK43_PLAF7	vor [d]	YNVSFIQNKTKR	37
VQ	I2B4_PLAF7	throcyte binding antigen-181	NRNSFVQRSYIR	9-122
	H496_PLAF7	ticulocyte binding protein homologue 4	LNSFVQINSSN	3-116
	IIM2_PLAF7	omboid-like protein [d]	SQSSFVQRSKPI	35
	I4R5_PLAF7	optry neck protein 3	LSVSFVQSLRGI	0-213
IL/IQ [c]	6279_PLAF7	toadherence linked asexual protein 2 [d]	EVPSMLQKFYIY	8-591
FE	7283_PLAF7	cyst capsule protein	DNSFFEELDKE	65-2668
	I5P0_PLAF7	forin-like protein 2	NNTSFFEKWVHS	3-746

[a] Peptide Match search performed for each consensus motif for *P. falciparum* PMX on UniProt Protein Information Resource[1] and the invasion and egress proteins were identified. Multiple isoforms of PfEMP1, Rhoptry-associated membrane antigen and Rifin were excluded from the Table. [b] Consensus motif in bold text. [c] Leu and Ile treated as equivalent in the searches indicated. [d] Multiple isoforms of the protein were found. Highlighted in yellow are proteins investigated in peptide cleavage experiments by Favuzza et al.[2] Highlighted in blue are liver stage proteins.

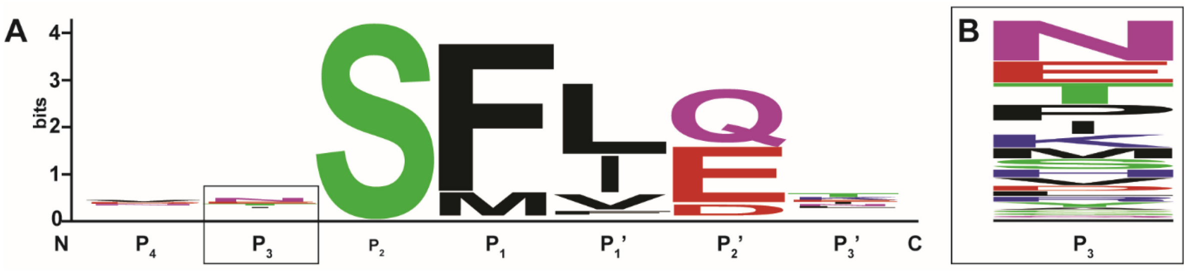

Figure S2. Abundance plot of amino acid positions P_4 to P_3' in the PfPMX consensus motif. **A.** Sequence alignment of invasion/egress proteins containing the PMX recognition motif and representation of the relative abundance of conserved amino acid residues in the consensus sequence using the WebLogo online resource.[3] The height of each letter (y-axis) is proportional to the frequency of that amino acid in that position (x-axis). **B.** An enlargement of the P_3 position showing the most common amino acids in that position.

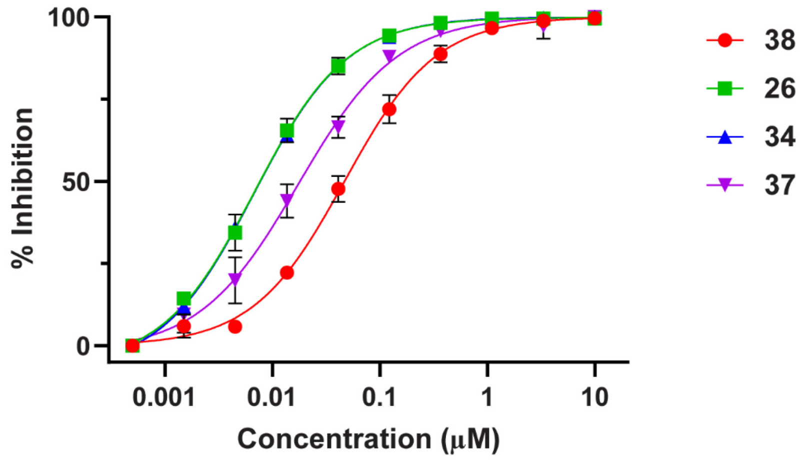

Figure S3. PMX protease dose-response curves of representative analogues. Compounds **26**, **34**, **37** and **38** were tested in a ten-point dilutions series for their ability to inhibit PMX protease activity. Each compound was incubated for 4 h at 37 °C with recombinant PfPMX. IC_{50} data represents the mean and SD for 3 independent fluorogenic substrate (Rh2N) cleavage experiments.

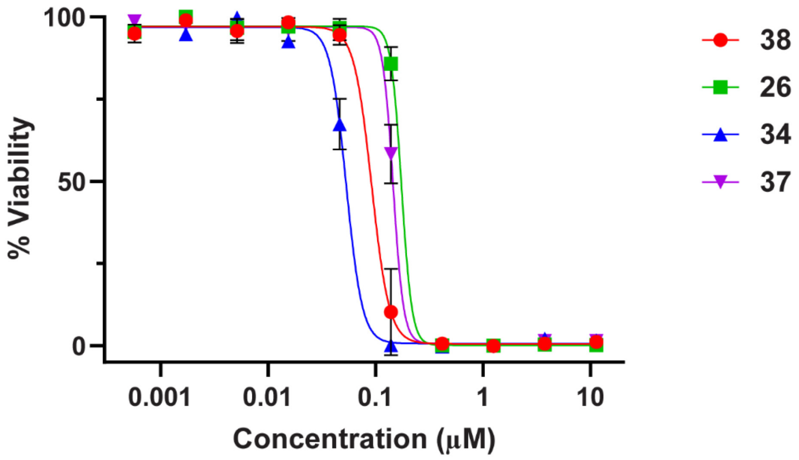

Figure S4. *P. falciparum* viability dose-response curves of representative analogues. Compounds **26**, **34**, **37** and **38** tested in a ten-point dilutions series for their ability to reduce parasite viability. *P. falciparum* parasites were exposed to the specified compound for 72 h at 37 °C. EC_{50} data represents the mean and SD for 3 independent experiments measuring LDH activity.

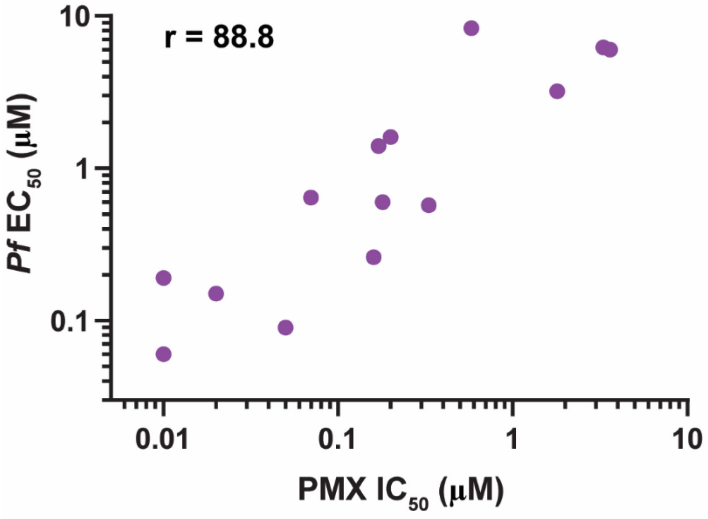

Figure S5. The correlation of *P. falciparum* viability EC_{50} data and PMX biochemical IC_{50} data for tested analogues. Each data point plotted represents the IC_{50} versus EC_{50} data for an analogue tested in the biochemical PMX assay and *P. falciparum* viability assay, respectively. IC_{50} data is the mean of 3 or more replicate experiments. EC_{50} data is the mean of 3 or more replicate experiments. Analogues that were inactive in the concentration range of either assay were excluded from the analysis. The analysis performed was the Pearson correlation analysis (GraphPad Prism 8.0.2) where 'r' is the correlation coefficient.

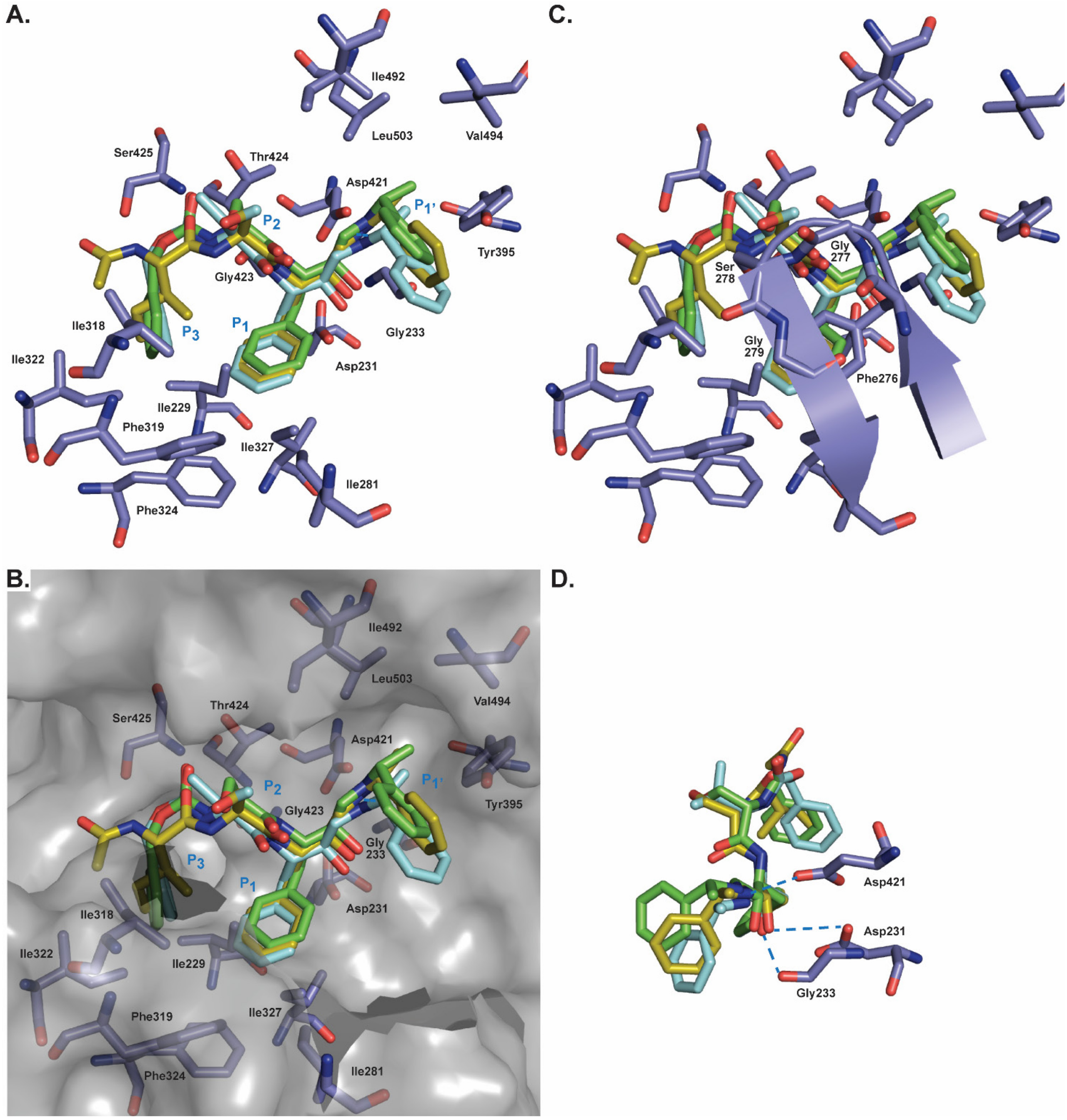

Figure S6. Model of peptidomimetics **26** (yellow), **34** (cyan) and **38** (green) in complex with PMX. **A.** The model showing interactions of the P3 to P_1' positions of the peptidomimetics with amino acids in each corresponding pocket of PMX (blue). **B.** Surface representation of the model showing substrate binding pockets of PMX accommodating the P_3 to P_1' moieties of peptidomimetics. For A. and B. positions of structural moieties of peptidomimetics are labelled relative to their respective binding pockets; PMX flap amino acids Ile274 to Ile281 were excluded for clarity. **C.** PMX flap amino acids Ile274 to Ile281 are included to show the flap position relative to the peptidomimetics. **D.** Shown are the hydrogen bonds of the HEA motif of peptidomimetics with Gly233 and the catalytic Asp421 and Asp321. The model was generated using the X-ray structure of the HEA compound MR0 C 803 bound to BACE-1 (PDB: 2P83)[4] as a template for positioning the peptidomimetics and then performing minimalization to the X-ray structure of PMX (PDB: 72BC).[2]

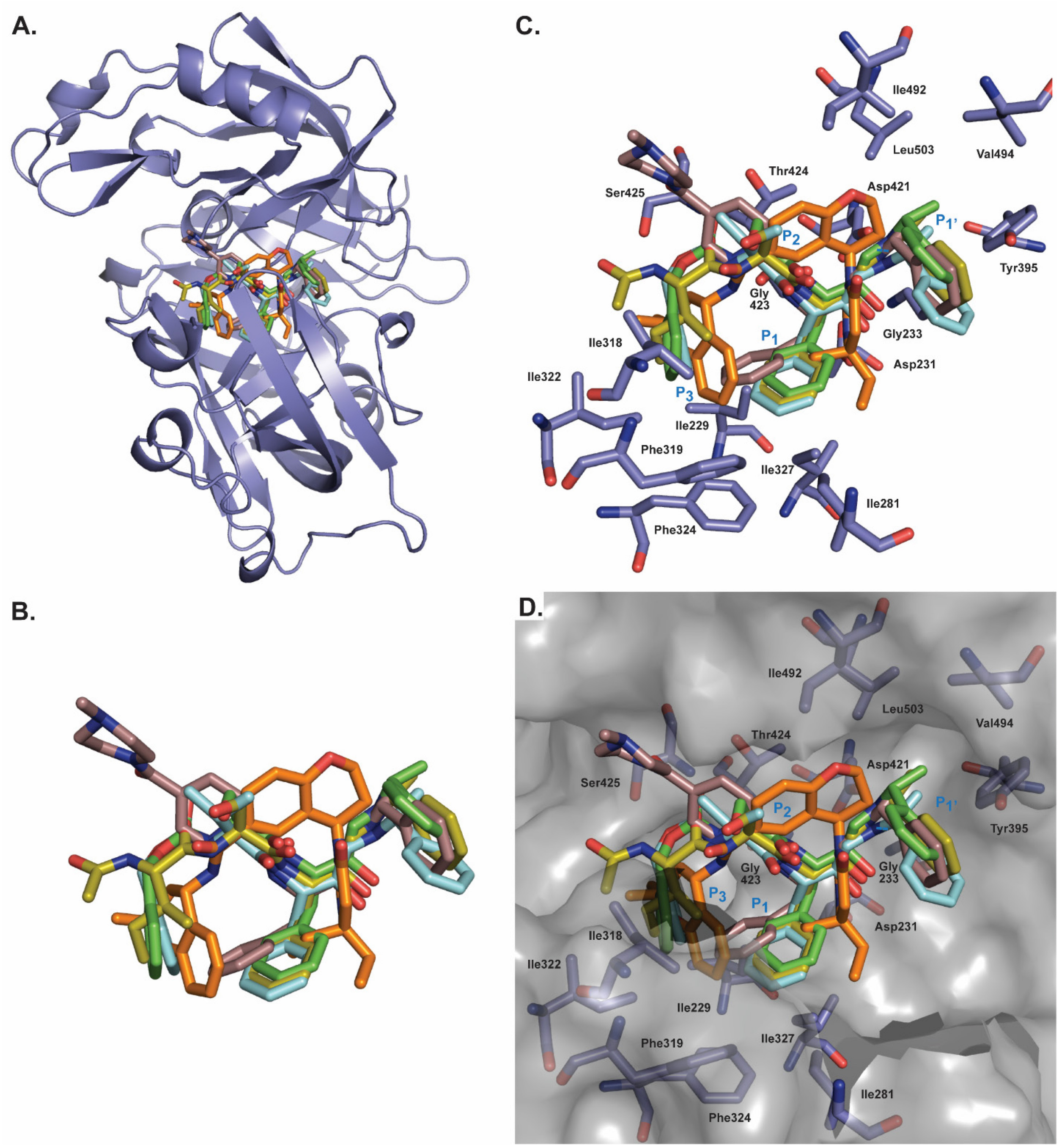

Figure S7. Model of peptidomimetics **26** (yellow), **34** (cyan) and **38** (green) overlaid with WM382 (orange) and 49c (brown) in complex with PMX. **A.** Overview of compounds bound to the substrate binding pocket of PMX. **B.** Overlay of peptidomimetics with PMX inhibitors WM382 and 49c showing their binding orientation to PMX. **A.** The model showing interactions of the P3 to P_1' positions of the peptidomimetics and WM382 and 49c with amino acids in each corresponding pocket of PMX (blue). **B.** Surface representation of the model showing substrate binding pockets of PMX accommodating the P_3 to P_1' moieties of peptidomimetics and WM382 and 49c. Positions of structural moieties of peptidomimetics are labelled relative to their respective binding pockets. PMX flap amino acids Ile274 to Ile281 were excluded for clarity. The model was generated using the X-ray structure of the HEA compound MR0 C 803 bound to BACE-1 (PDB: 2P83)[4] as a template for positioning the peptidomimetics and 49c and then performing minimalization to the X-ray structure of PMX (PDB: 72BC).[2]

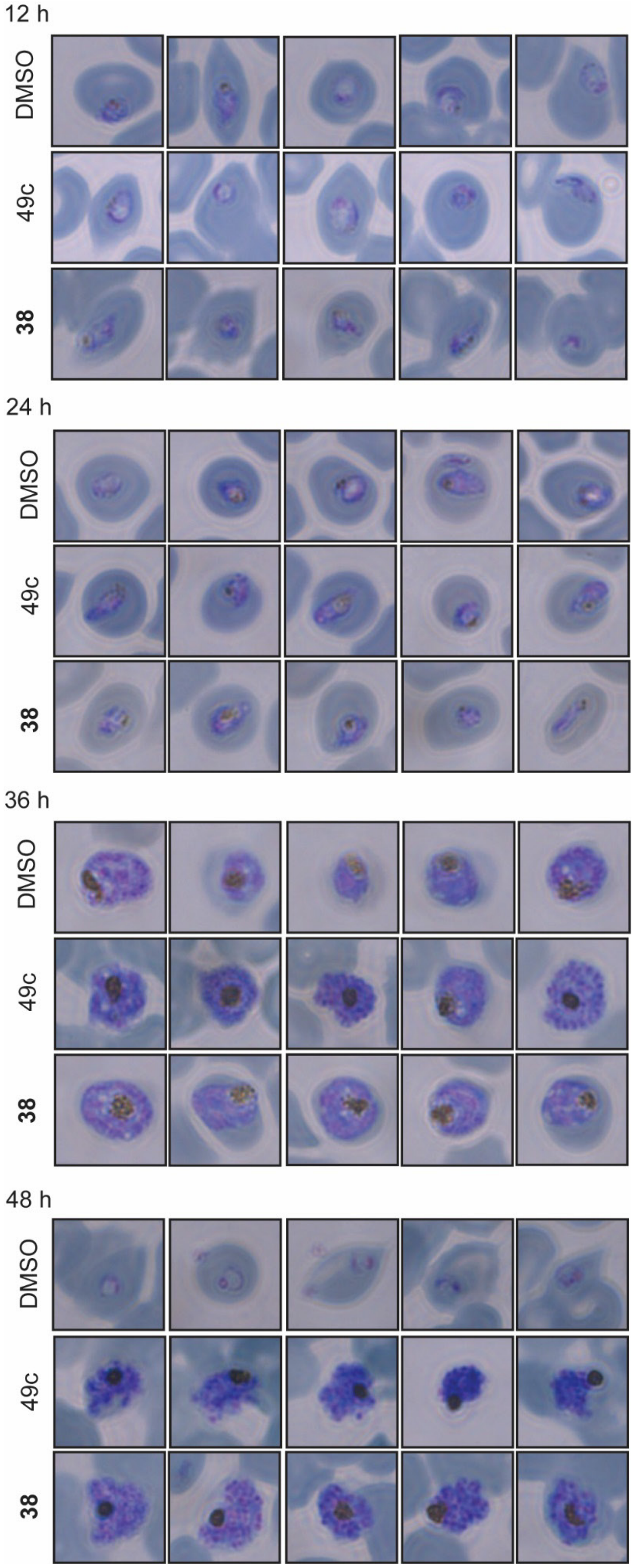

Figure S8. Replicate images of analogue **38** and 49c (**2**) blocking merozoite egress.

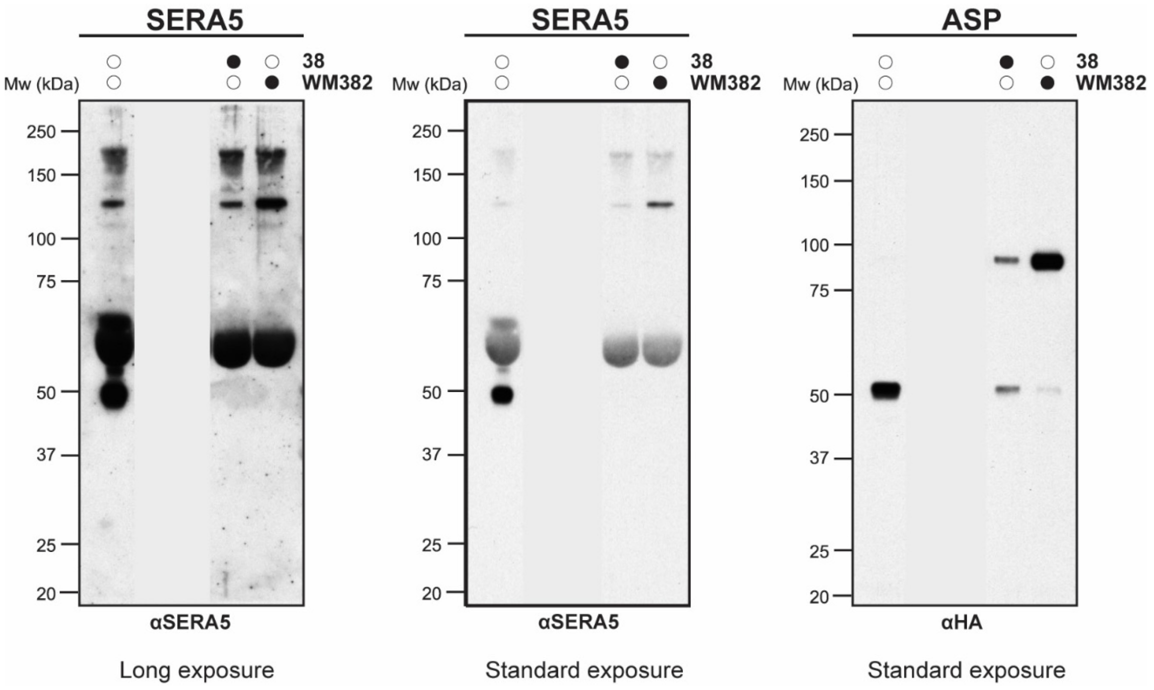

Figure S9. Uncut *P. falciparum* processing assay western blots. Uncut western blots from the PMX processing inhibition assay (long exposure left and standard exposure middle) and uncut PMIX processing inhibition assay (standard exposure) shown in Figure 5.

Table S2. Protease FRET assay conditions.

	PMX assay	PMIX	PMV assay	Renin assay	Cathepsin D assay	BACE-1 assay
Enzyme quantity	0.1 nM protease	1.5 nM protease	2.5 nM protease	3 nM protease (Proteos #R-001)	0.25 nM protease (Athens Research #16-12-030104)	6 nM protease (Sigma #S4195)
Buffer	25mM sodium acetate, 0.005% Tween-20, pH 5.5	25 mM sodium acetate, 100 mM sodium chloride and 0.005% Tween-20, pH 5.5	25 mM MES, 25 mM Tris-HCl, 0.01% Tween-20, pH 6.4	50 mM Tris, 100 mM NaCl, 0.1% Brij-35, pH 8.0	100 mM sodium acetate 0.02% Brij-35, pH 5.0	20 mM sodium scetate, 0.05% Brij-35, pH 4.5
Substrate peptide	DABCYL-HSFIQEGKEE-EDANS (Rh2N)	(DABCYL)-KEISFLERRE(EDANS) (PfRON3)	DABCYL-RNKRTLAQKQE-EDANS (KAHRP)	DABCYL-(γ-Abu)-IHPFHLVIHTE-EDANS	E[E(EDANS)]KPILFFRLGK(DABCYL)E	RE(EDANS)EVNLDAEFK(DABCYL)R
Peptide final conc.	3.5 μM	16.6 μM	12 μM	20 μM	0.5 μM	5 μM
Incubation time	4 h	4 h	90 min	45 min	30 min	240 min
Positive control compound and activity in assay	49c [5]<br>IC_{50} 1 nM	49c [5]<br>IC_{50} 15,100 nM	WEHI-842 [6]<br>IC_{50} 17 nM	Aliskiren [7]<br>IC_{50} 2 nM	Pepstatin A<br>IC_{50} 0.7 nM	Verubecestat [8]<br>IC_{50} 5 nM

NMR and LCMS data

22 C NMR

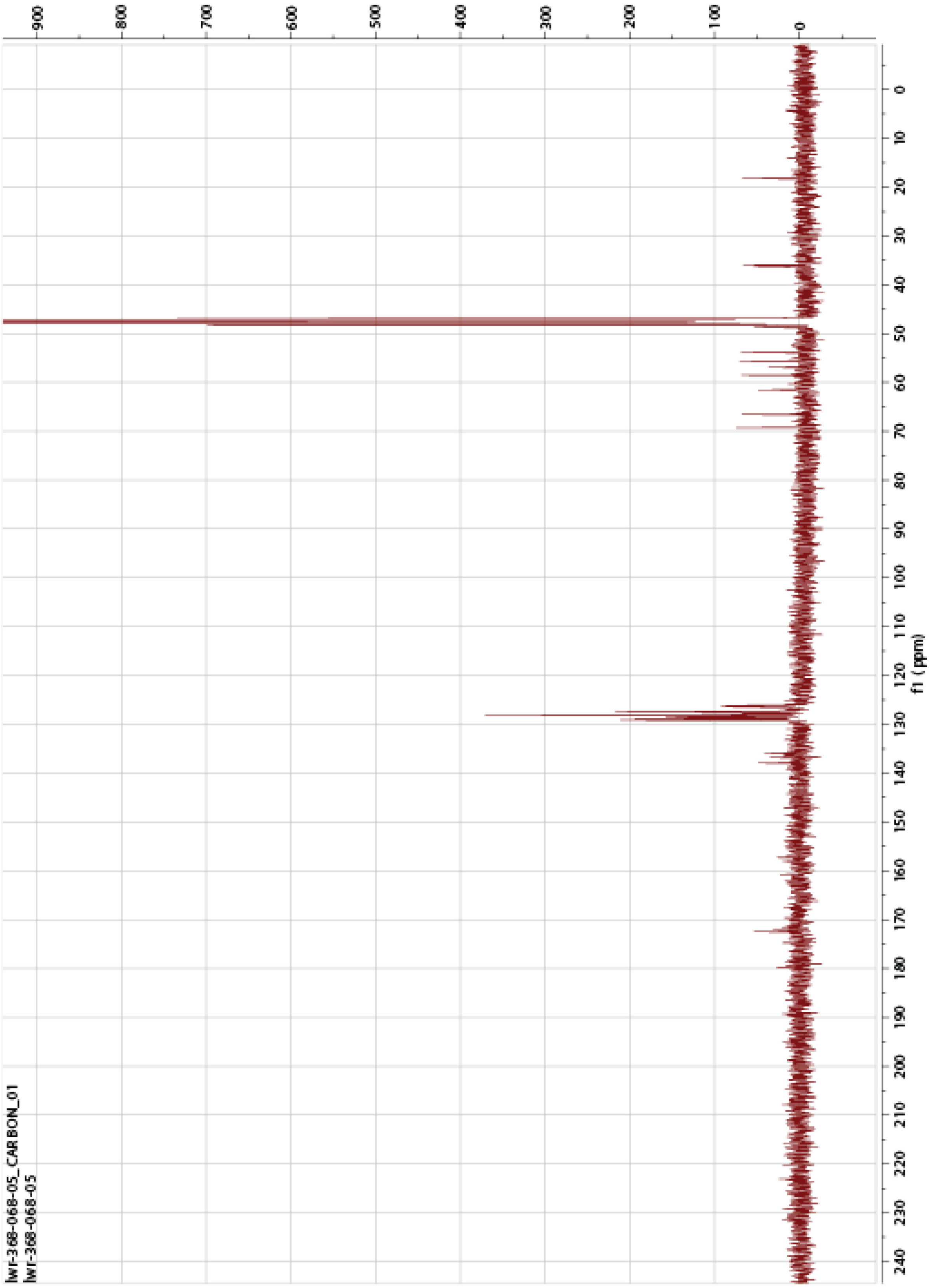

22 H NMR

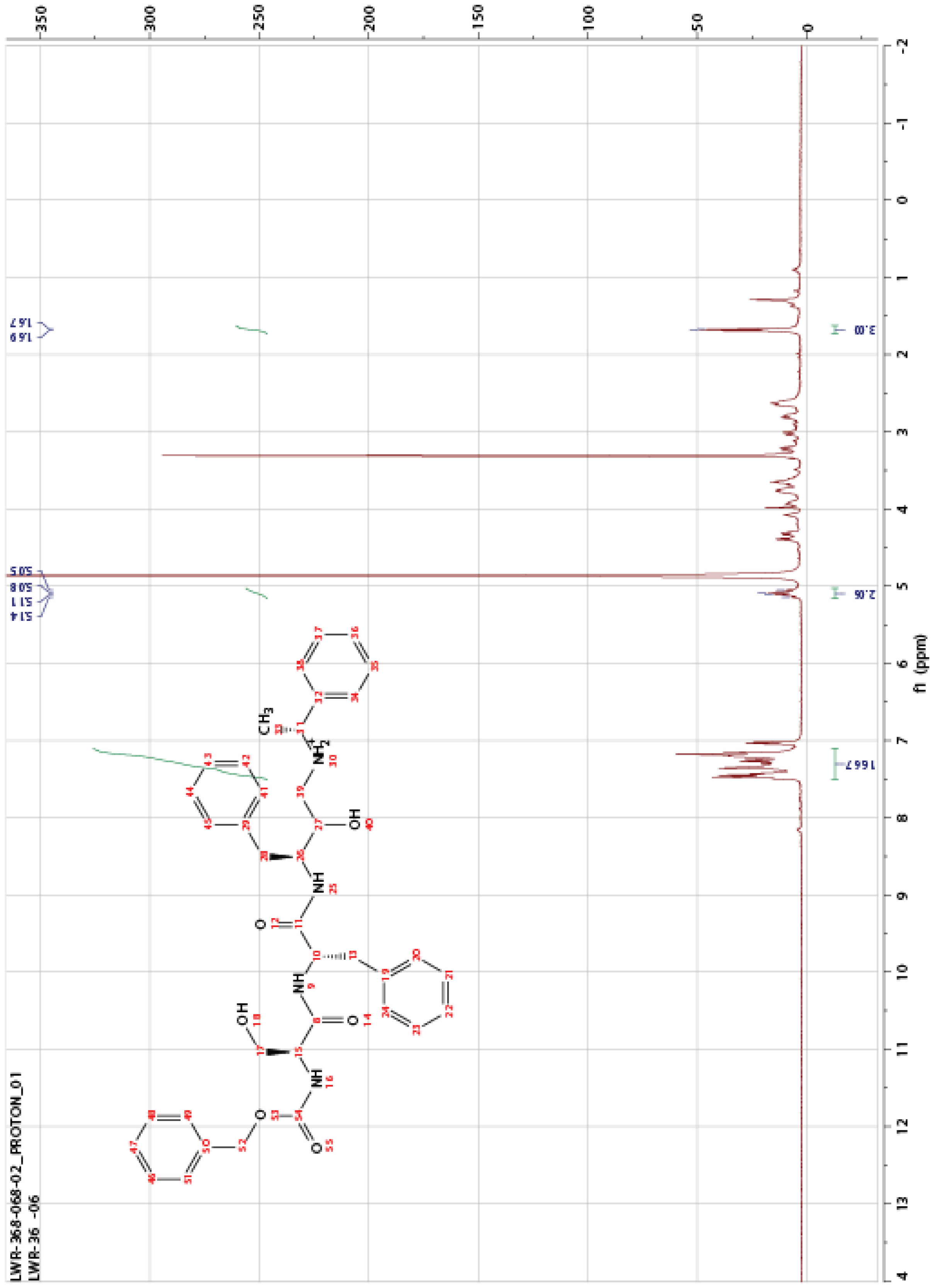

22 LCMS

Current Chromatogram(s)

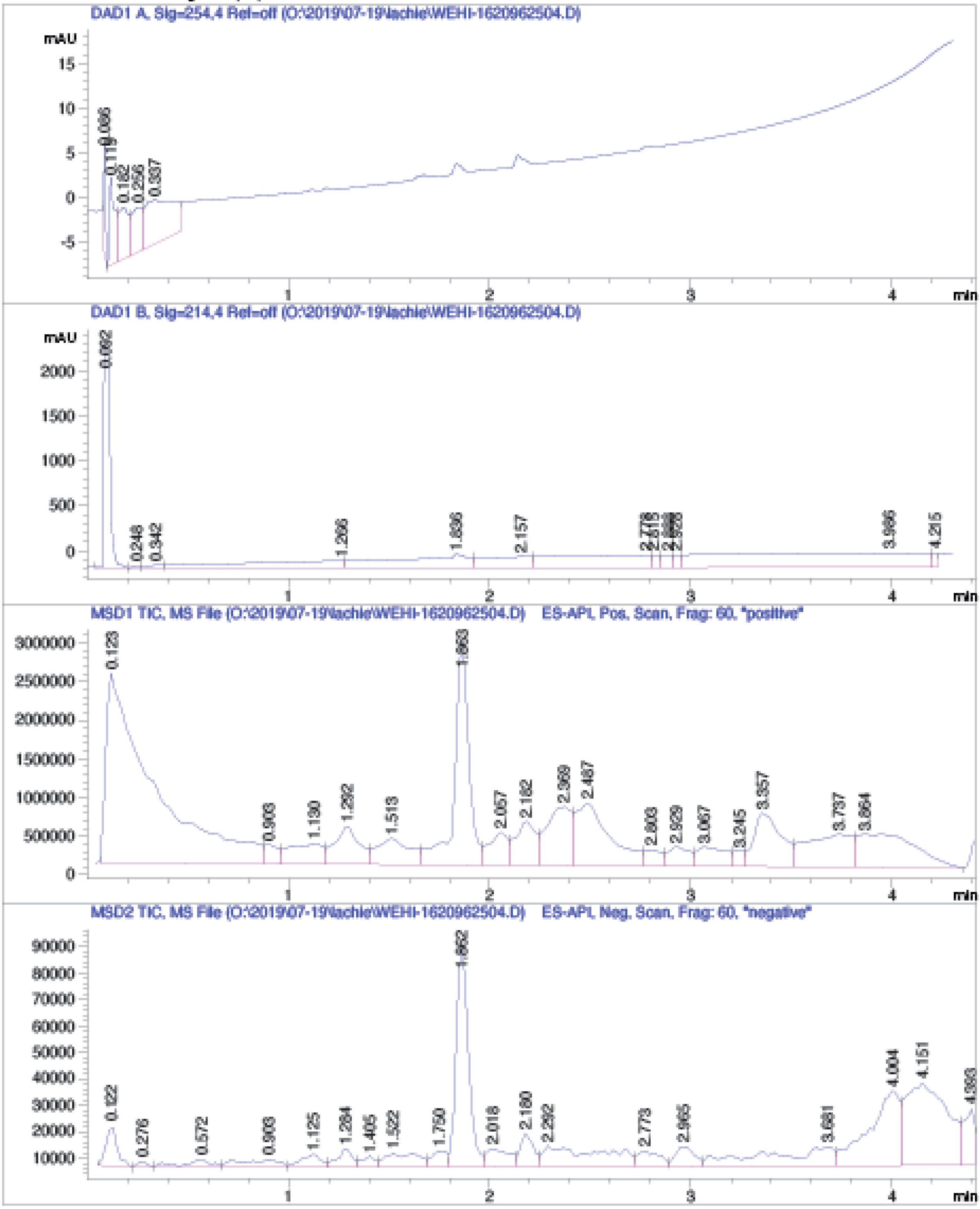

MS Spectrum

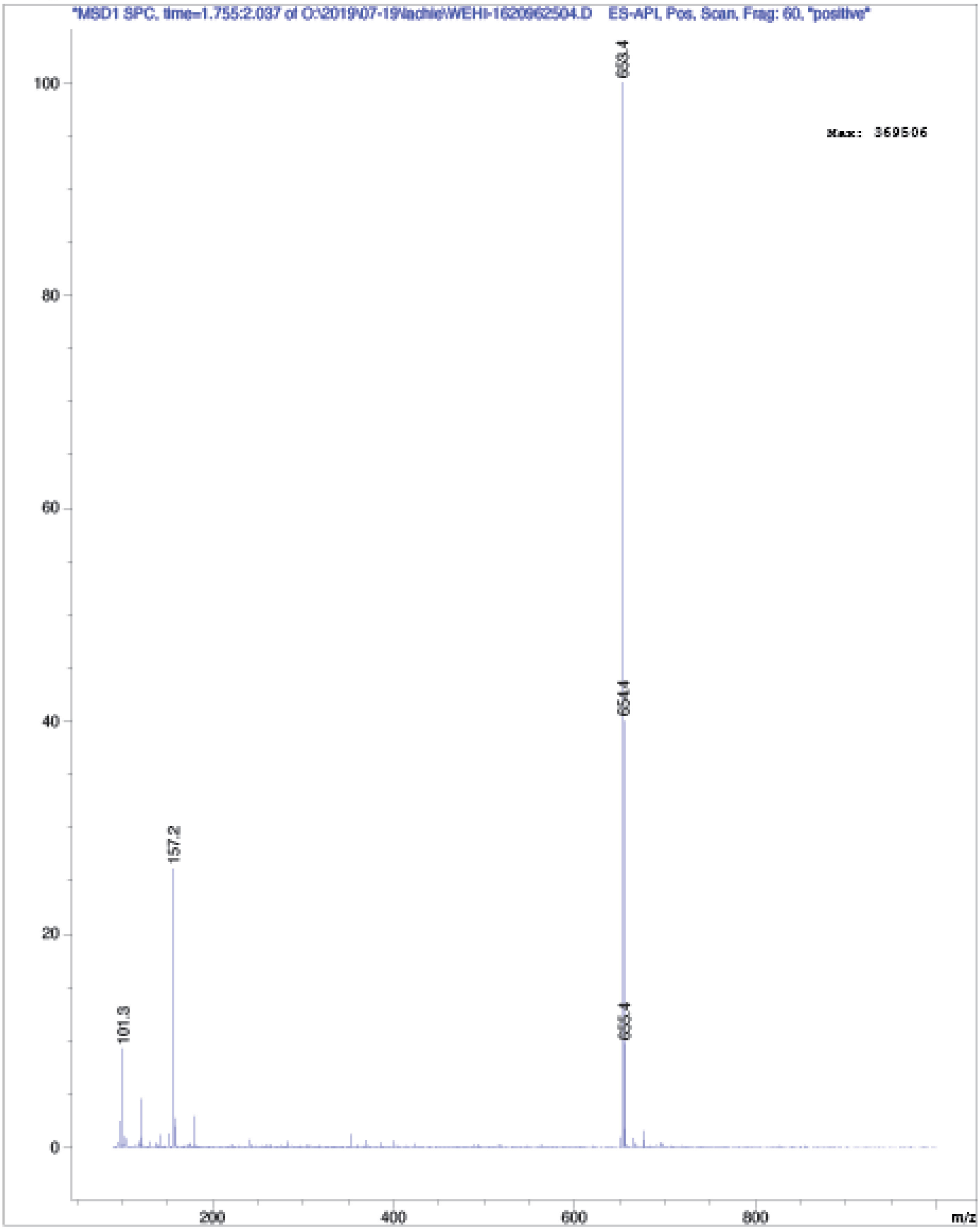
*MSD1 SPC, time=1.755:2.037 of O:\2019\07-19\lachie\WEHI-1620962504.D ES-API, Pos, Scan, Frag: 60, "positive"
Max: 369506
653.4
654.4
655.4
157.2
101.3
0
20
40
60
80
100
200
400
600
800
m/z

23 H NMR

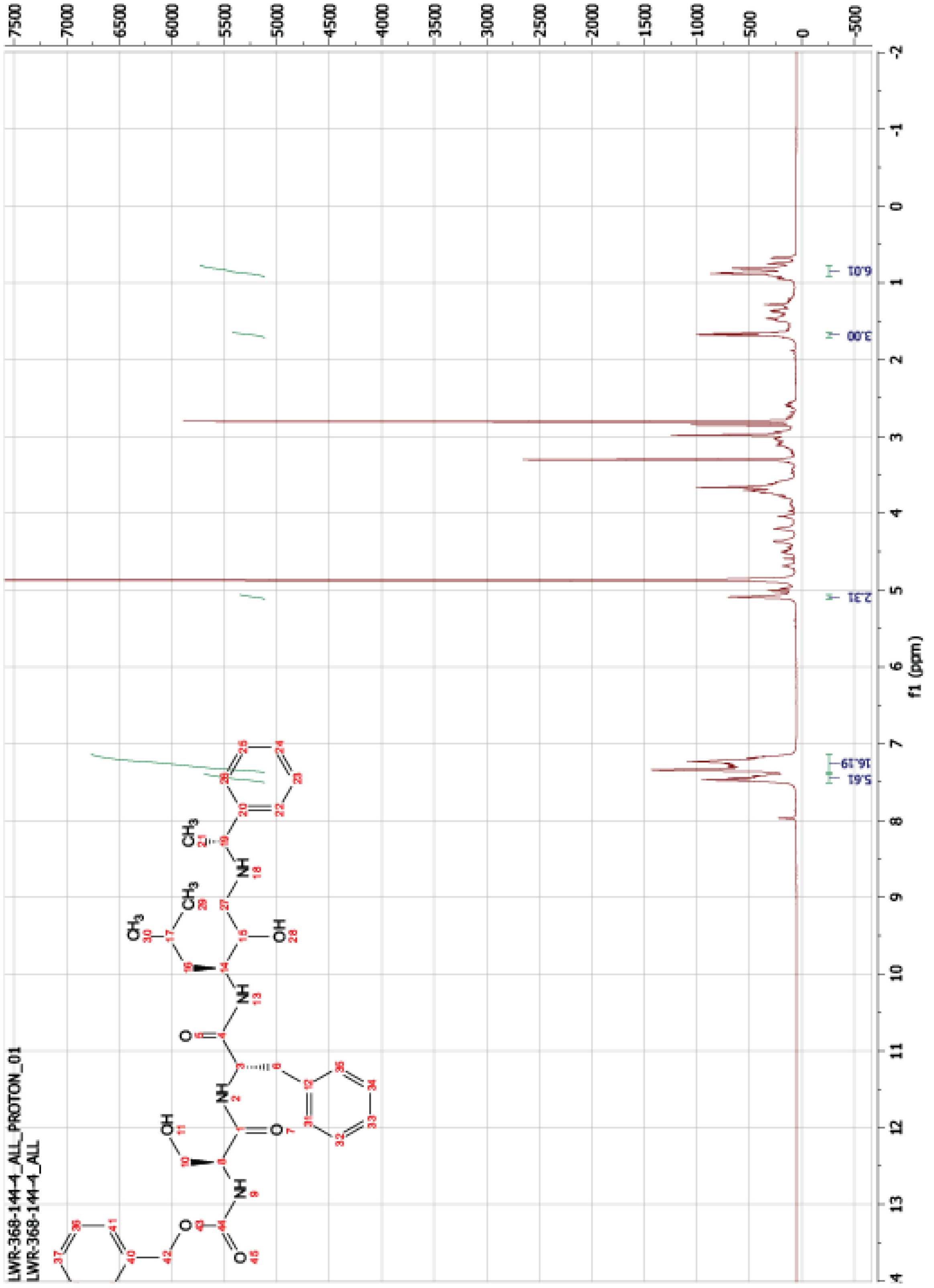

23 C NMR

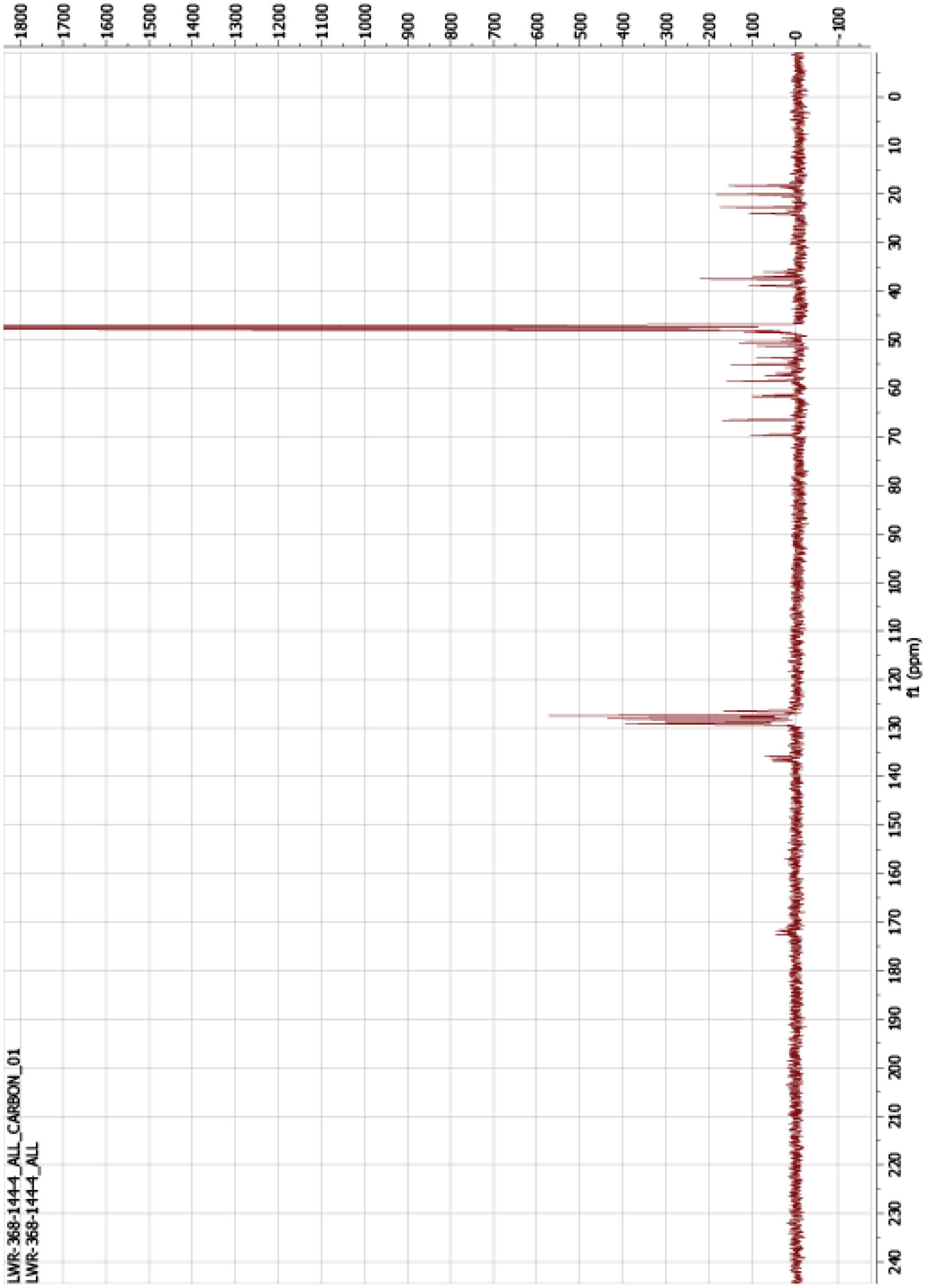

23 LCMS

Current Chromatogram(s)

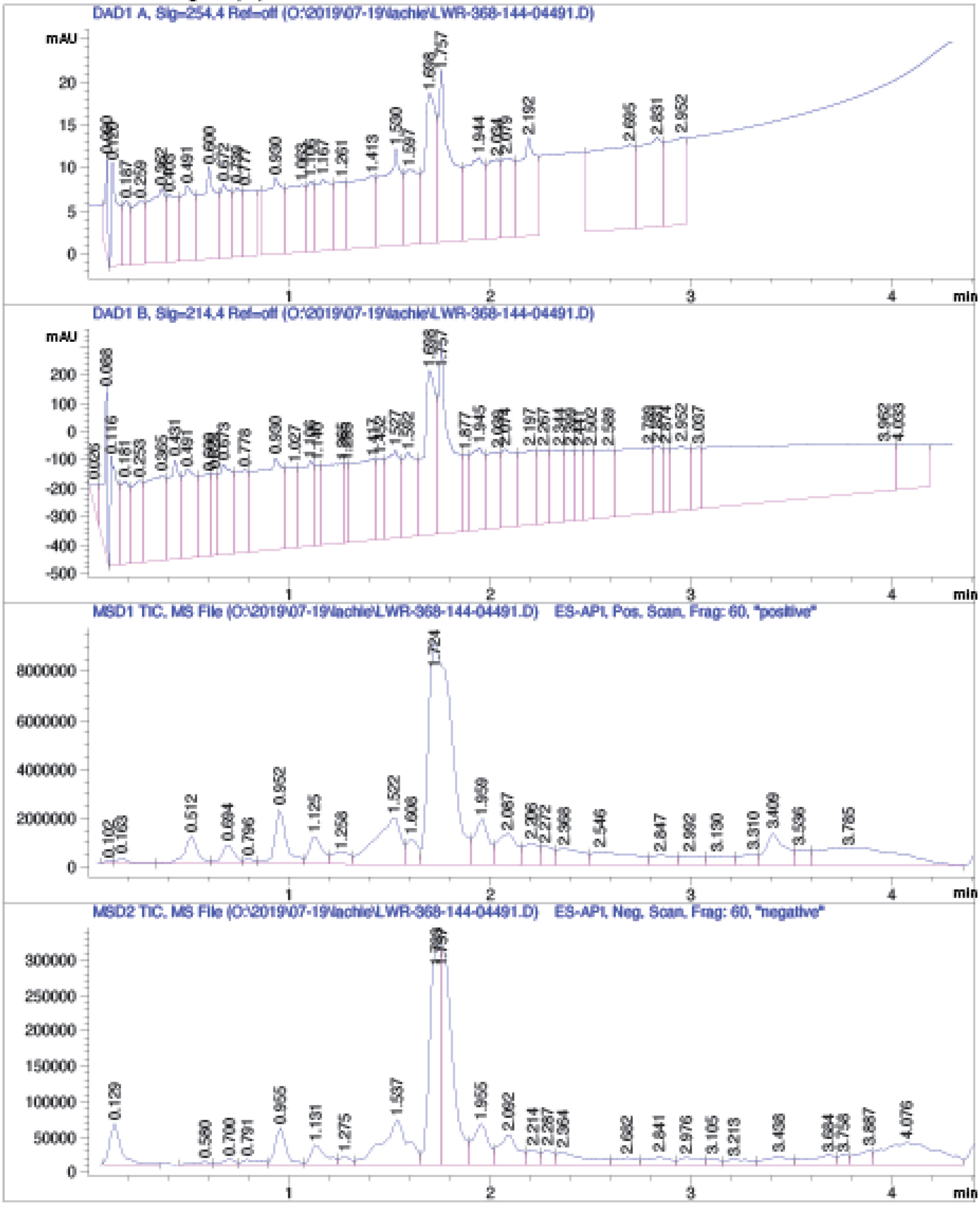

MS Spectrum

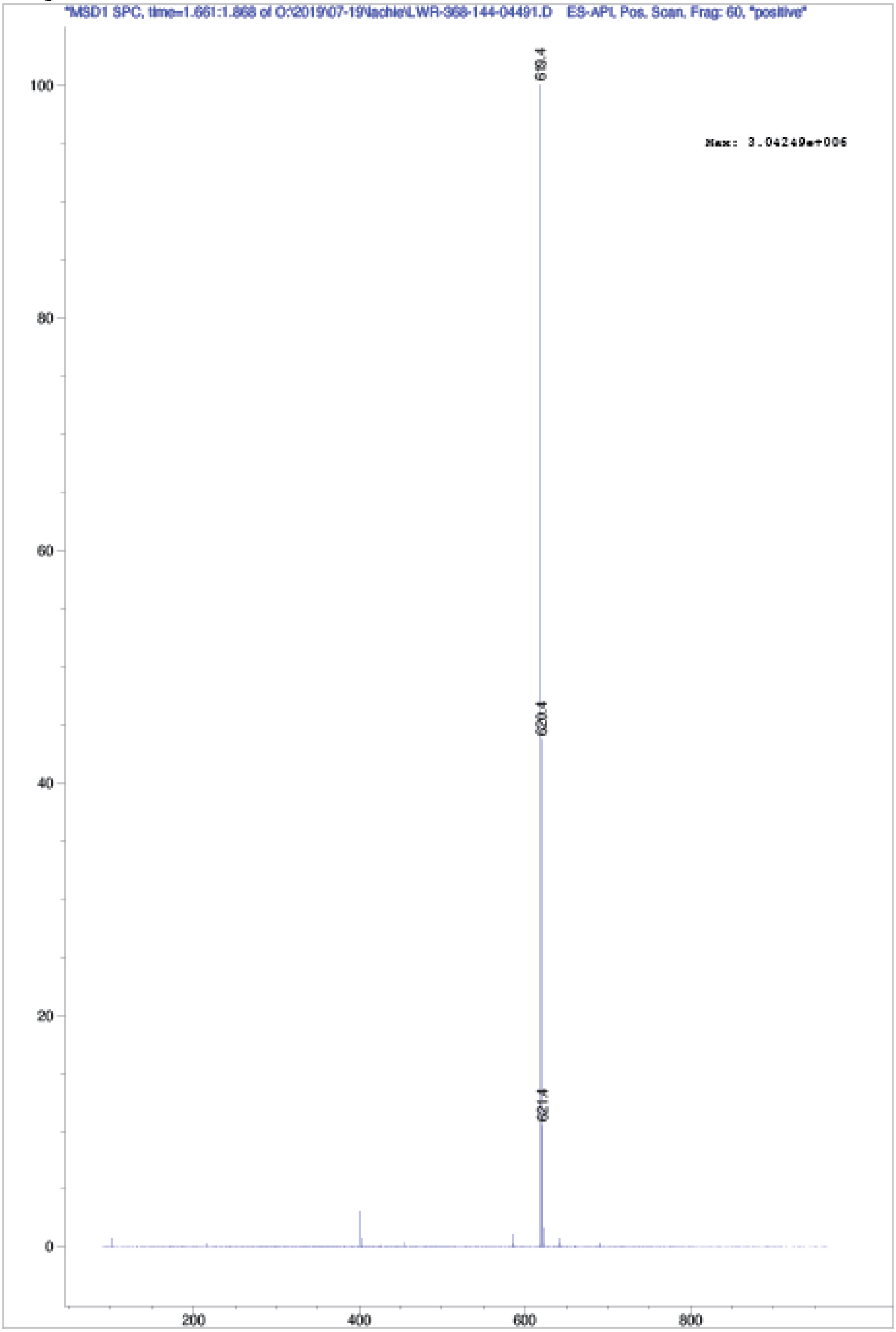
*MSD1 SPC, time=1.661:1.868 of O:\2019\07-19\lachie\LWR-368-144-04491.D ES-API, Pos, Scan, Frag: 60, "positive"
Max: 3.04249e+006
619.4
620.4
621.4
100
80
60
40
20
0
200
400
600
800

24 C NMR

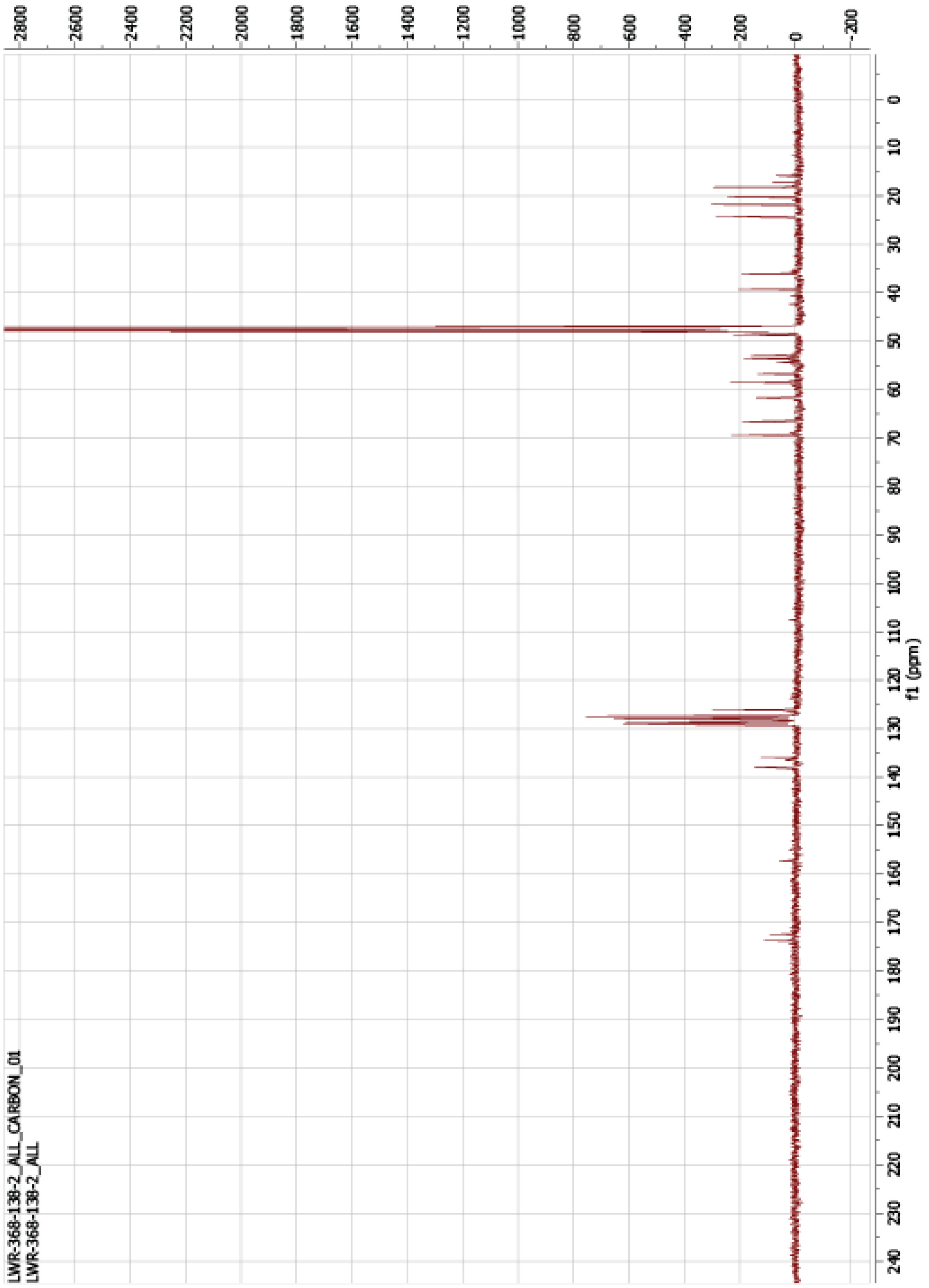

24 H NMR

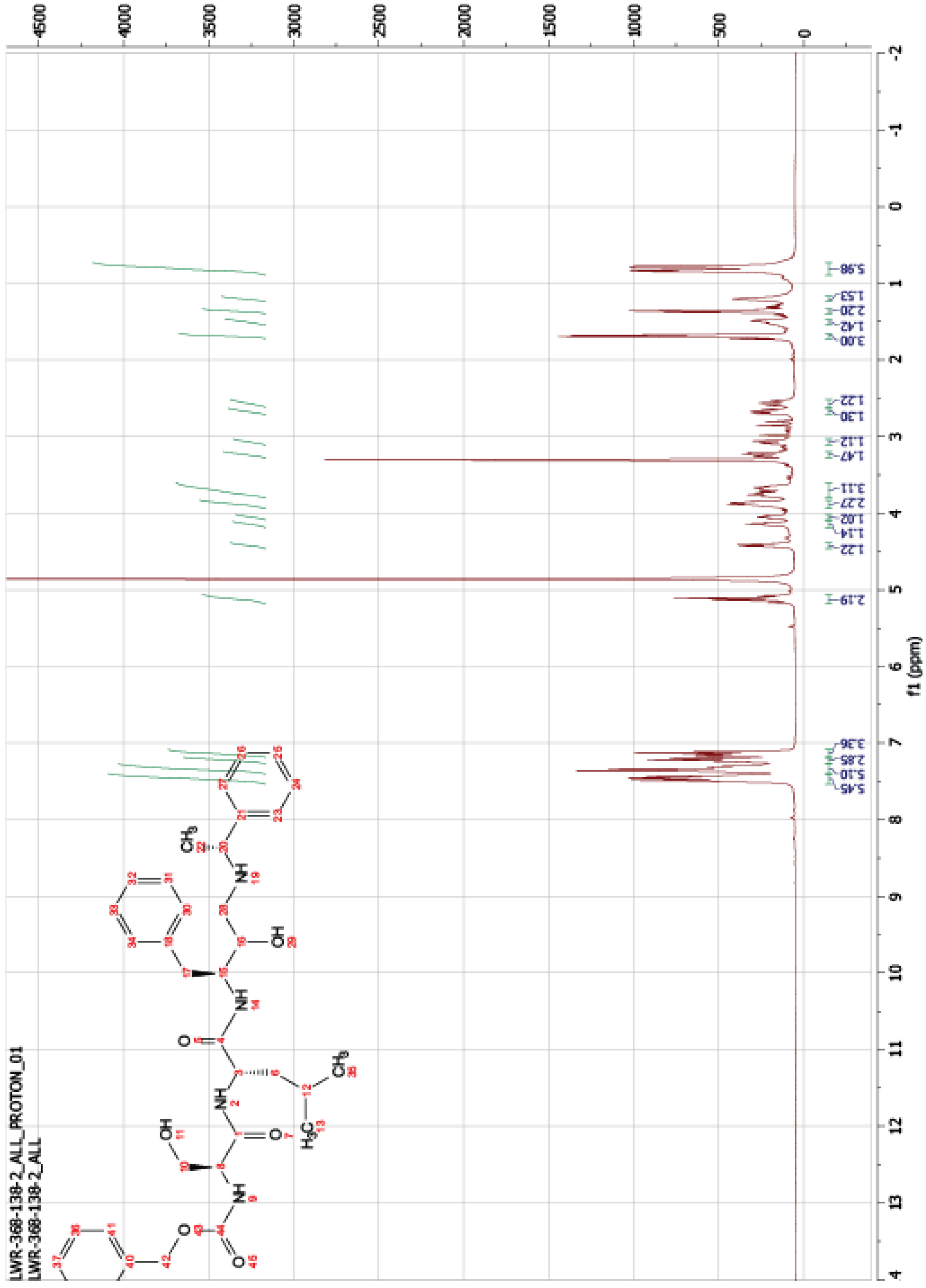

24 LCMS

Current Chromatogram(s)

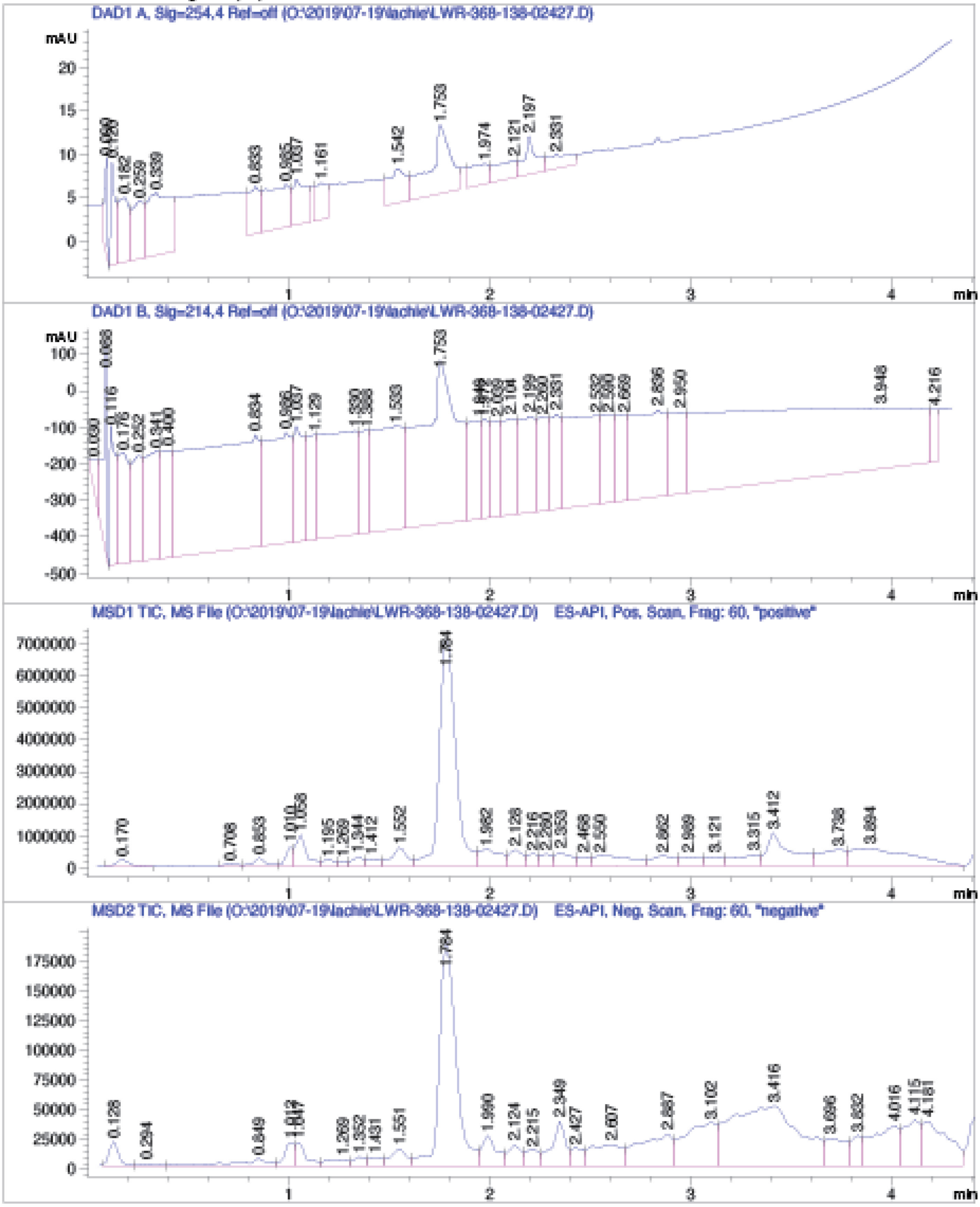

MS Spectrum

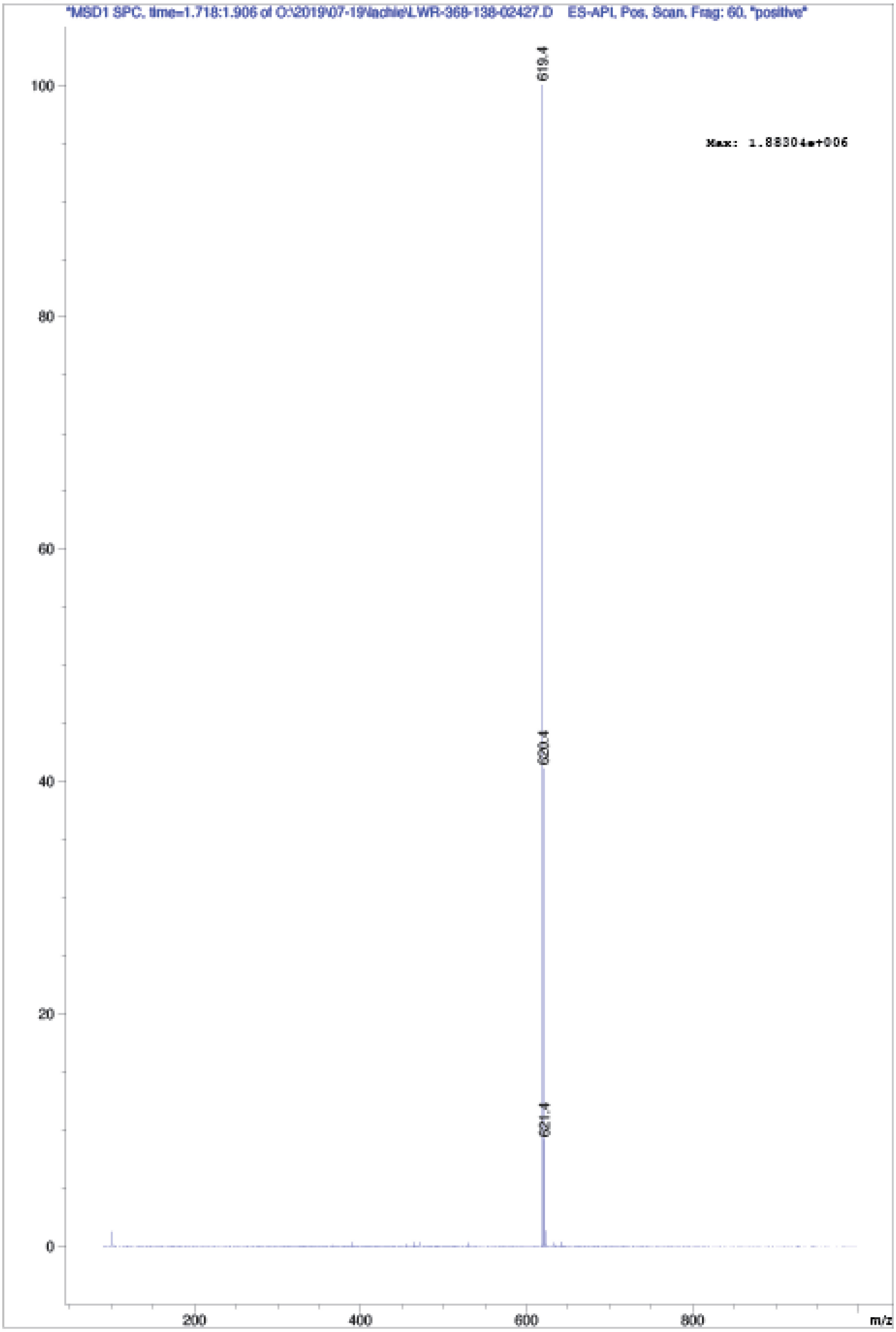

*MSD1 SPC, time=1.718:1.906 of O:\2019\07-19\achie\LWR-369-138-02427.D ES-API, Pos, Scan, Frag: 60, "positive"
Max: 1.88304e+006
619.4
620.4
621.4
100
80
60
40
20
0
200
400
600
800
m/z

25 C NMR

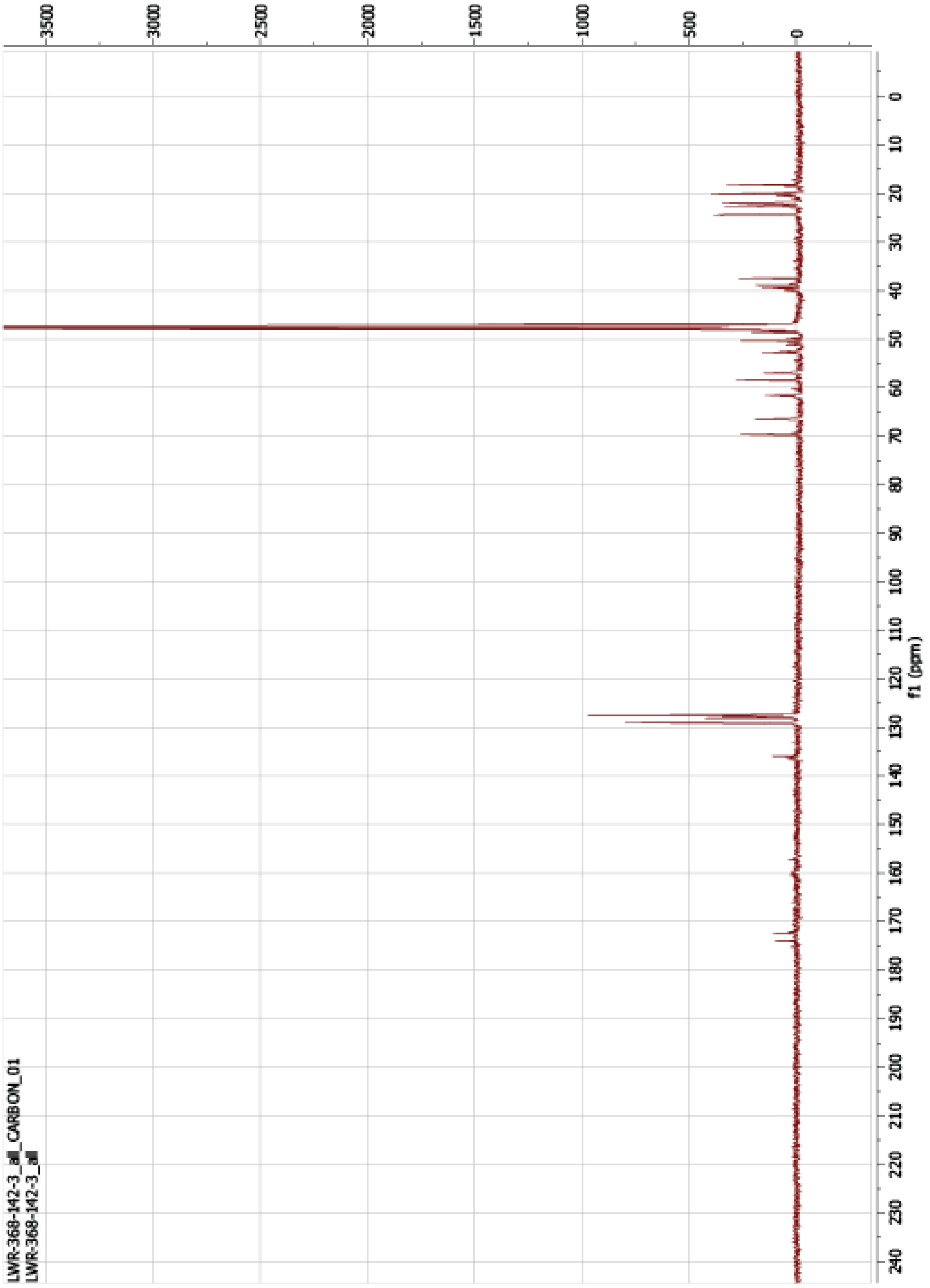

25 H NMR

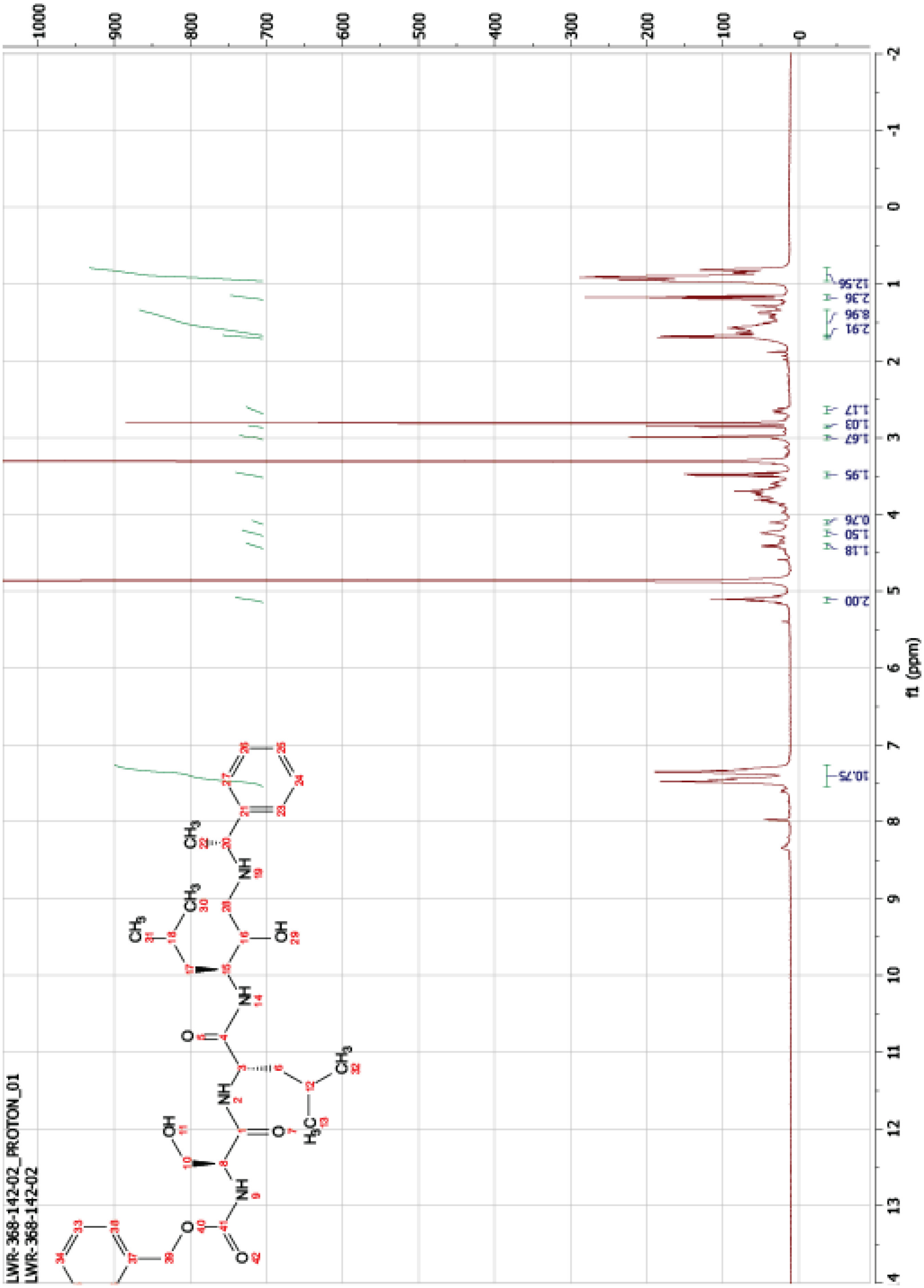

25 LCMS

Current Chromatogram(s)

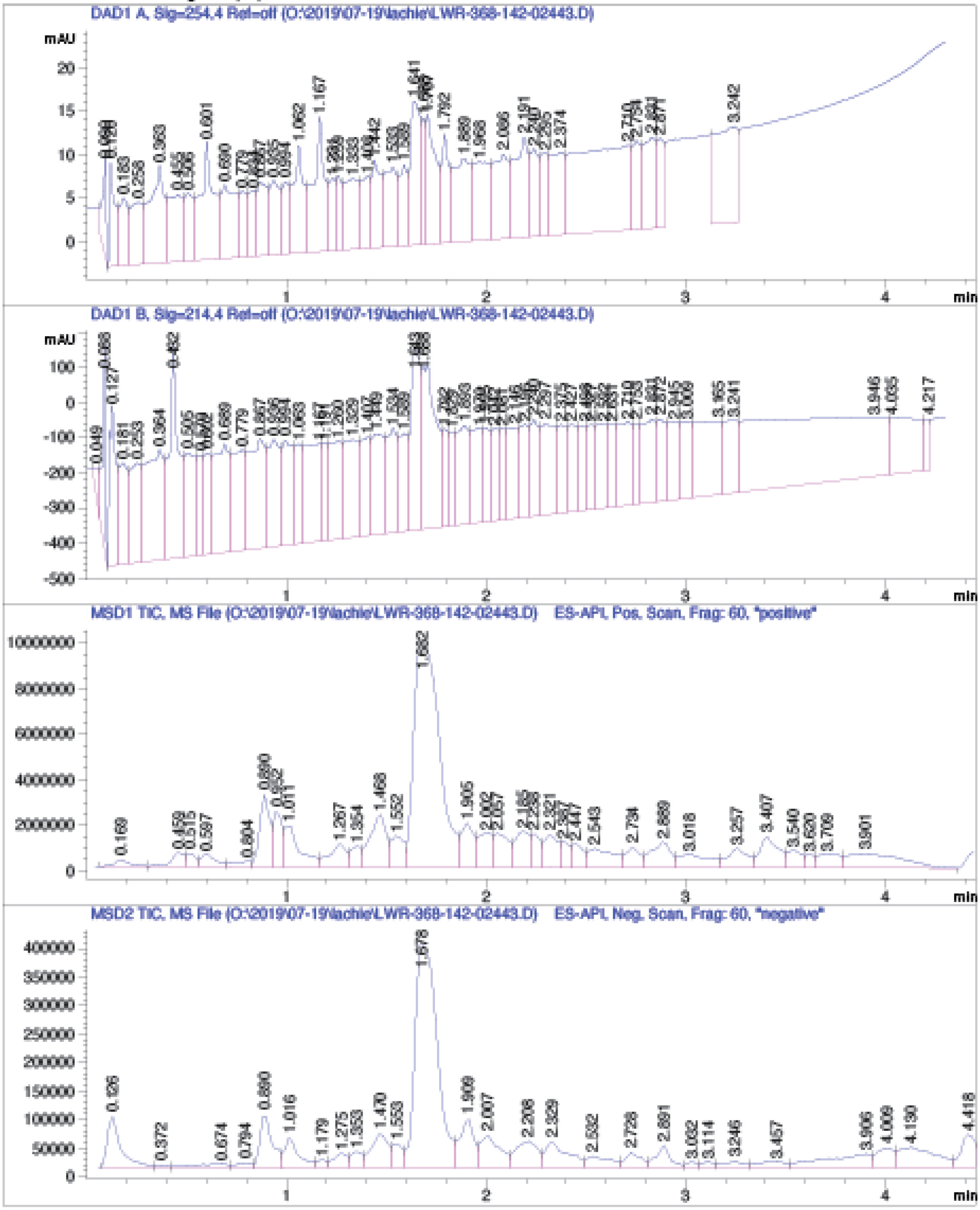

MS Spectrum

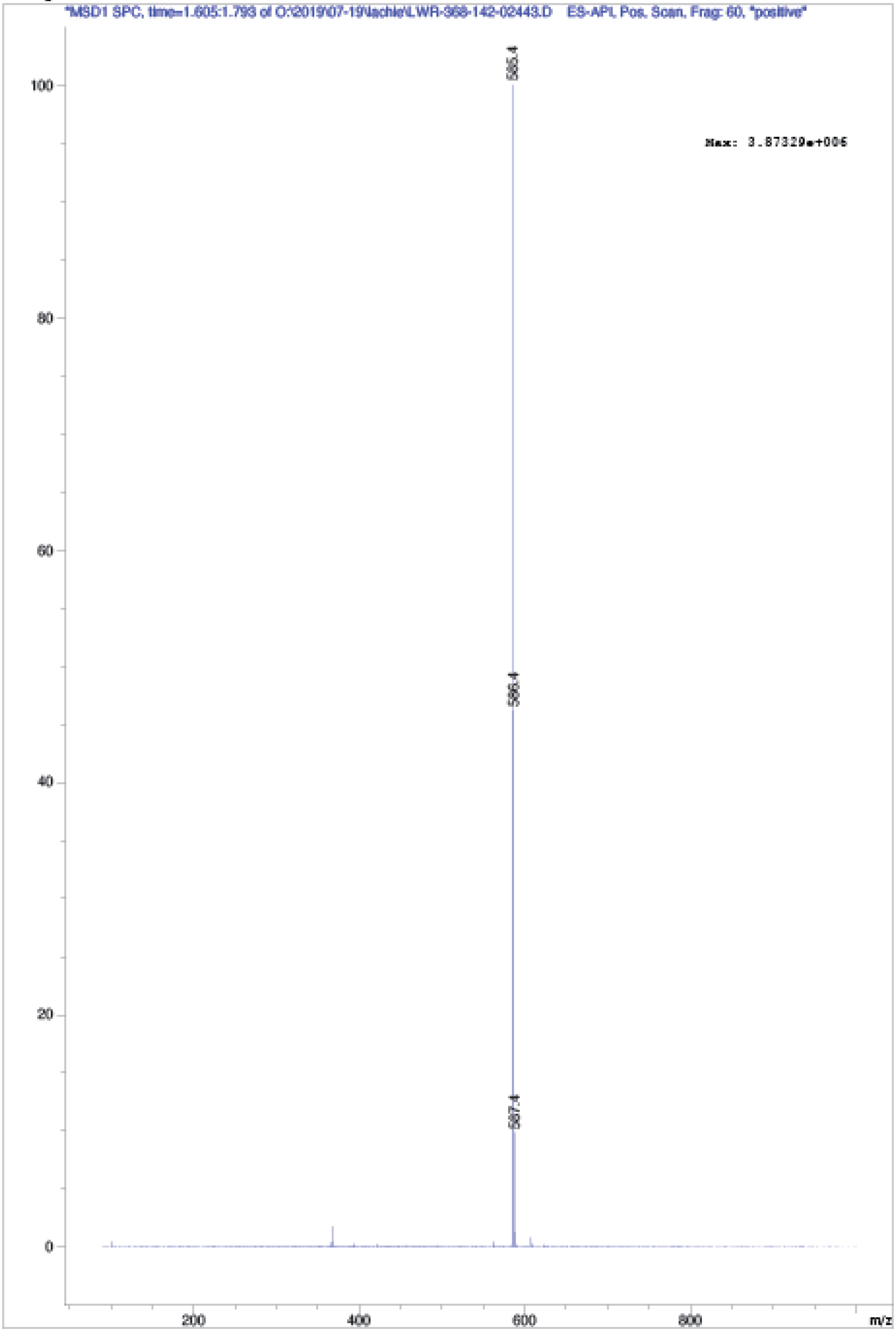

*MSD1 SPC, time=1.605:1.793 of O:\2019\07-19\lachie\LWR-368-142-02443.D ES-API, Pos, Scan, Frag: 60, "positive"
Max: 3.87329e+006
585.4
586.4
587.4
100
80
60
40
20
0
200
400
600
800
m/z

26 C NMR

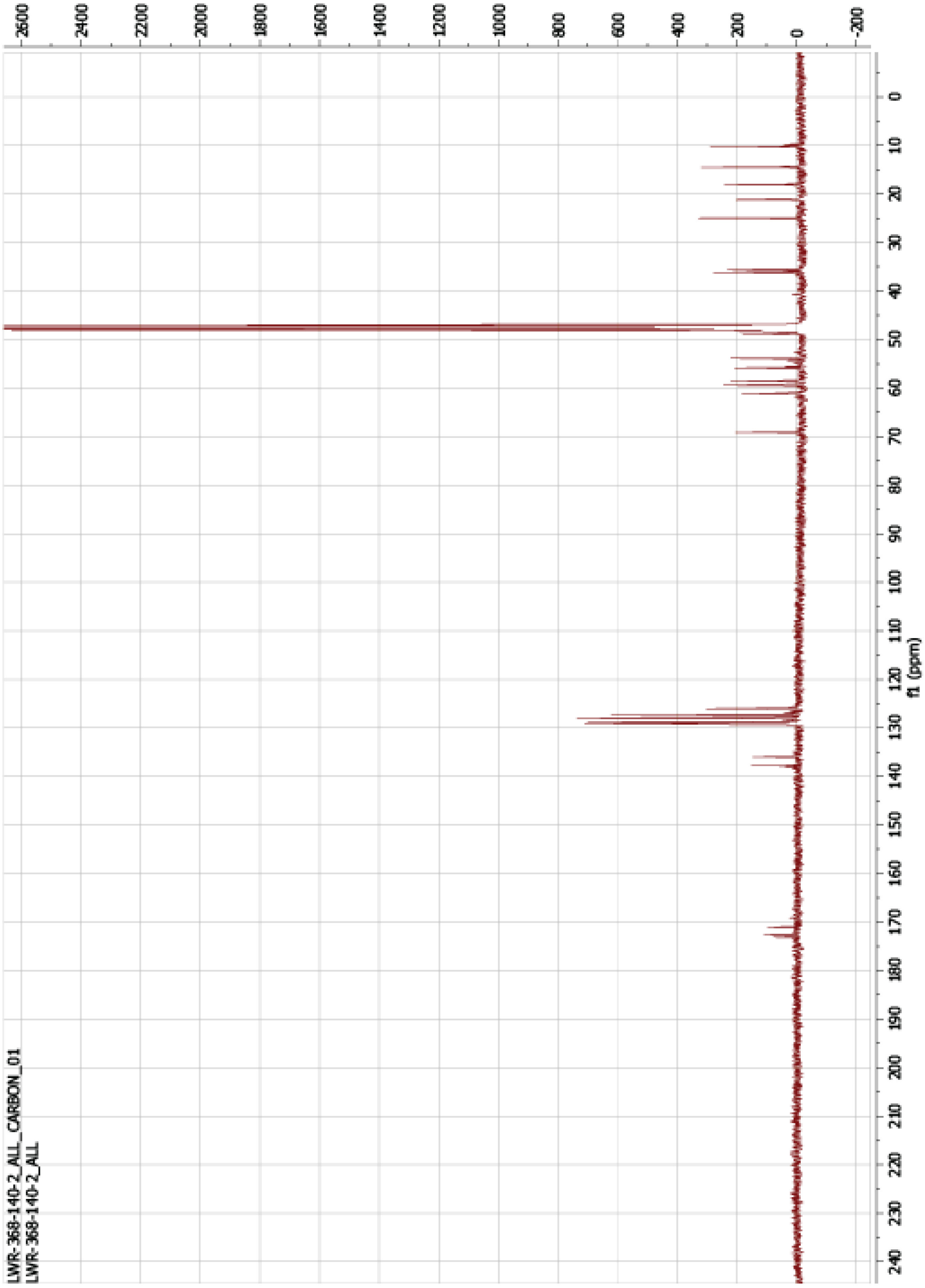

26 H NMR

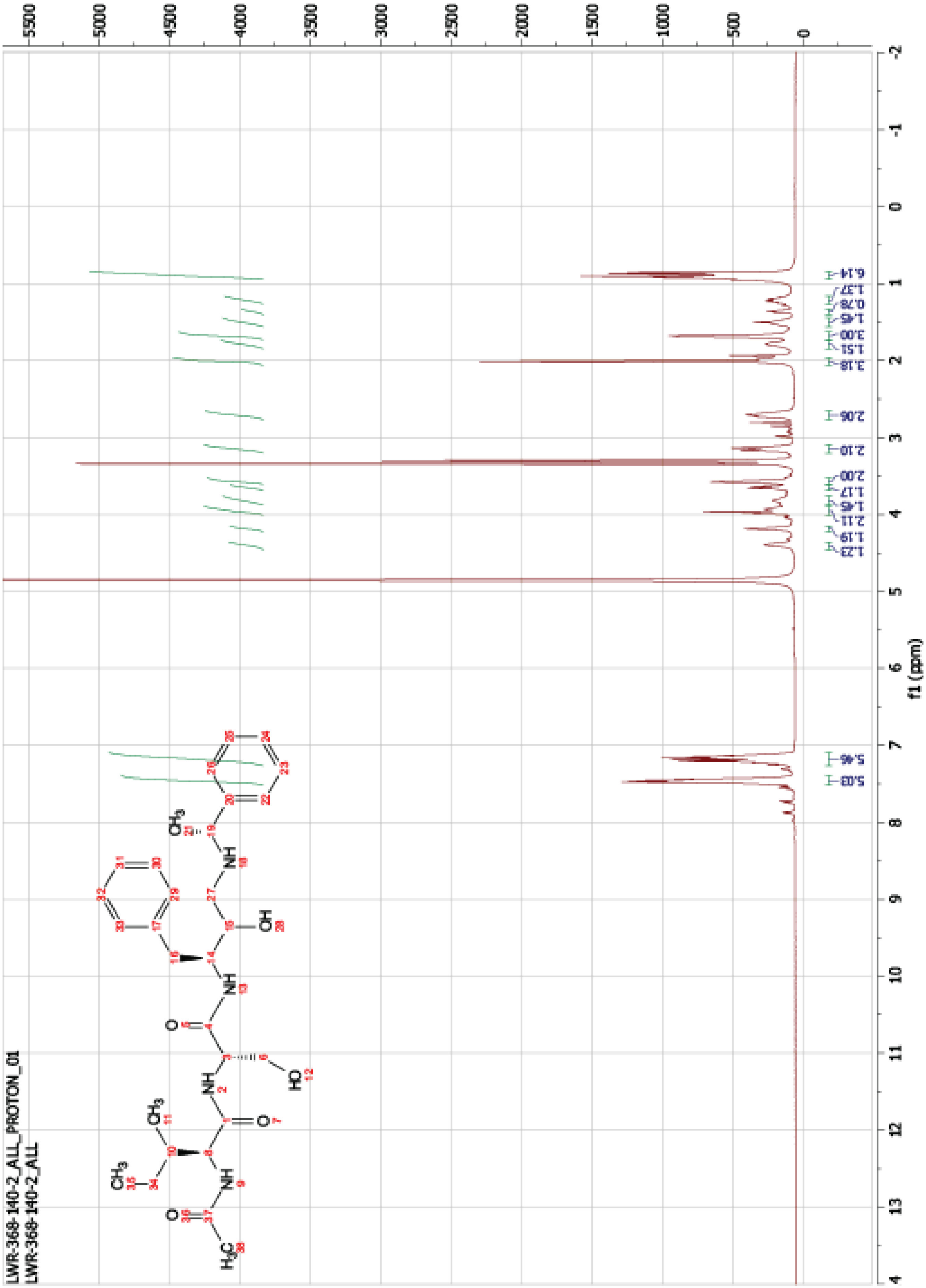

26 LCMS

Current Chromatogram(s)

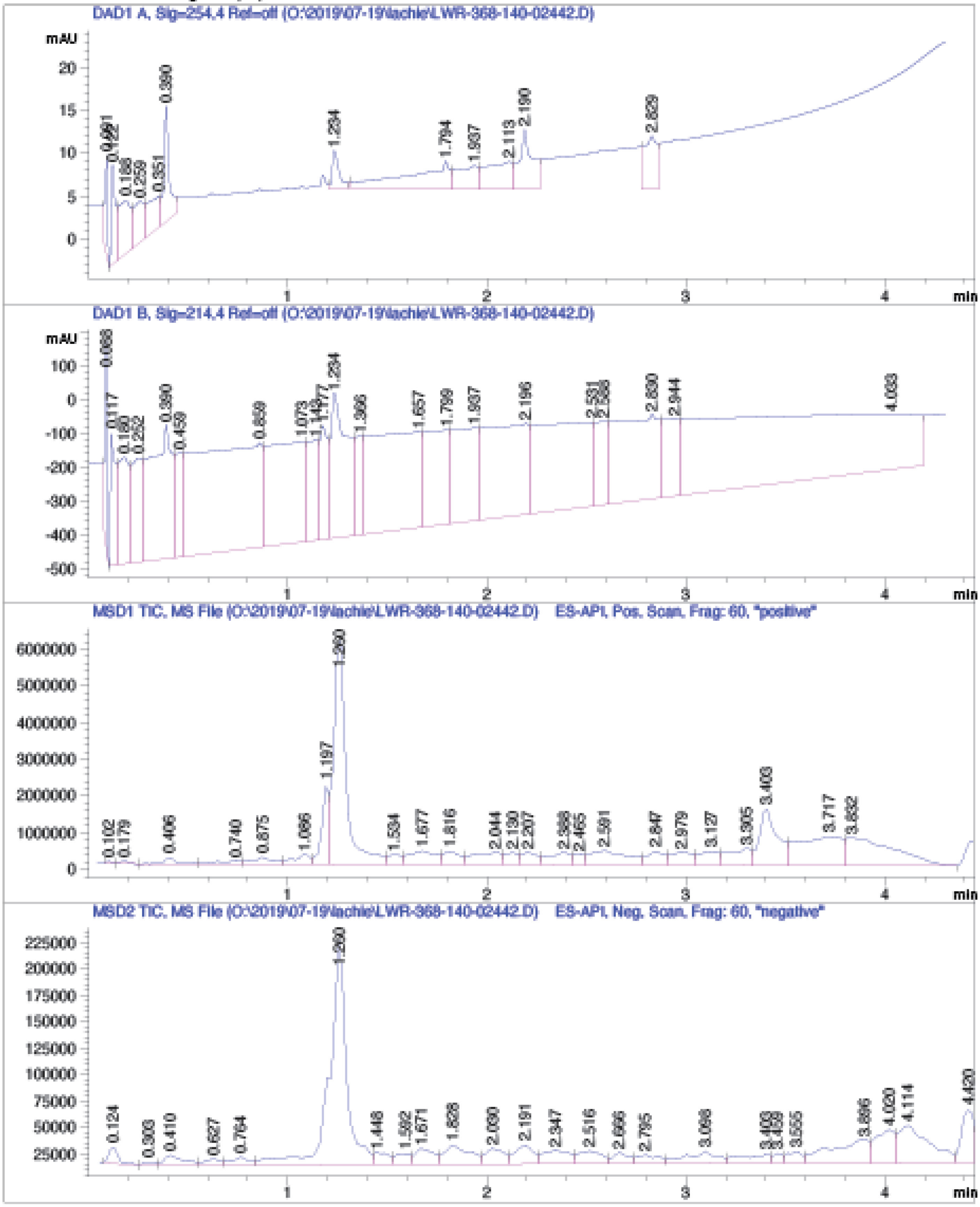

MS Spectrum

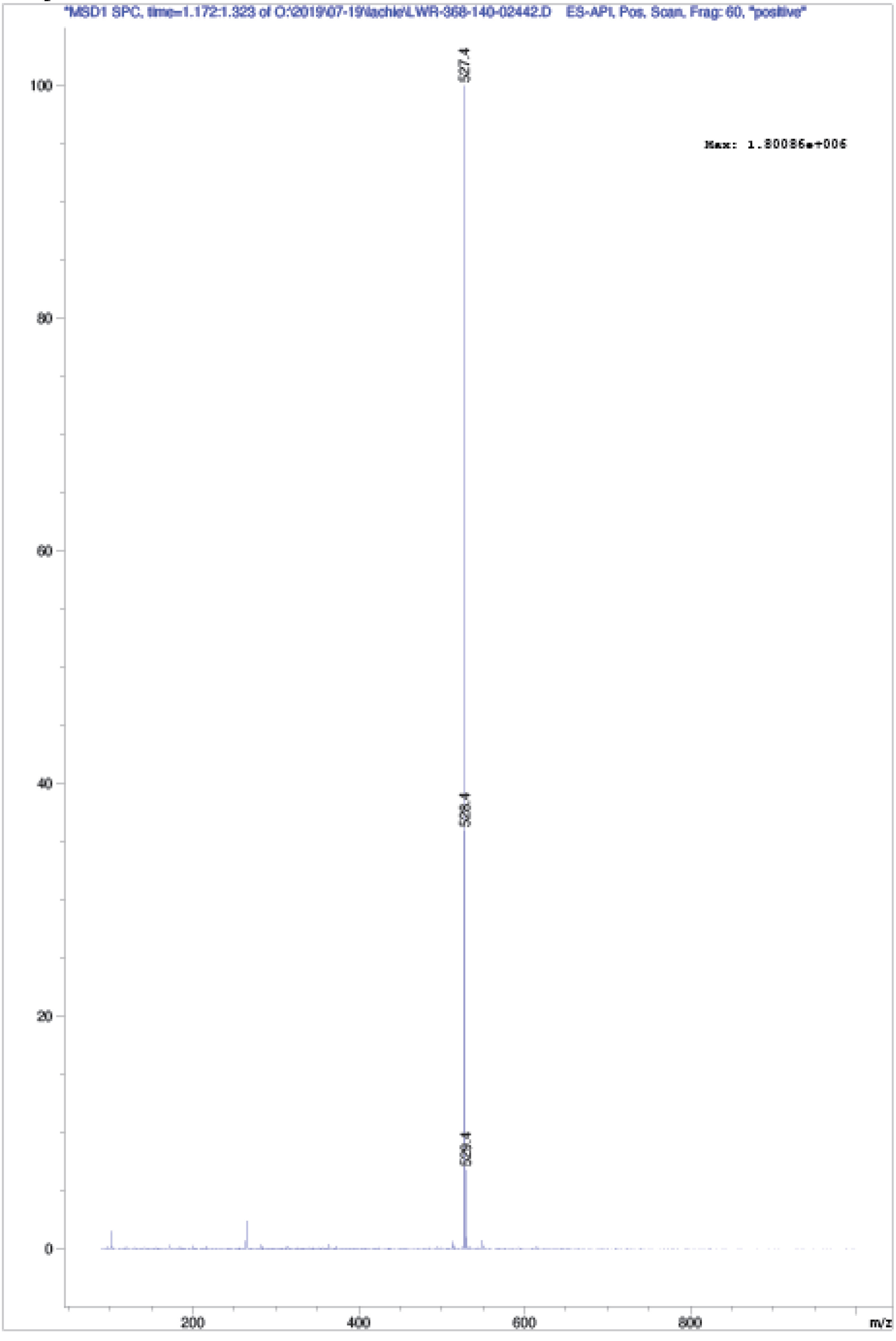

*MSD1 SPC, time=1.172:1.323 of O:\2019\07-19\lachie\LWR-368-140-02442.D ES-API, Pos, Scan, Frag: 60, "positive"
527.4
Max: 1.80086e+006
528.4
529.4
100
80
60
40
20
0
200
400
600
800
m/z

27 C NMR

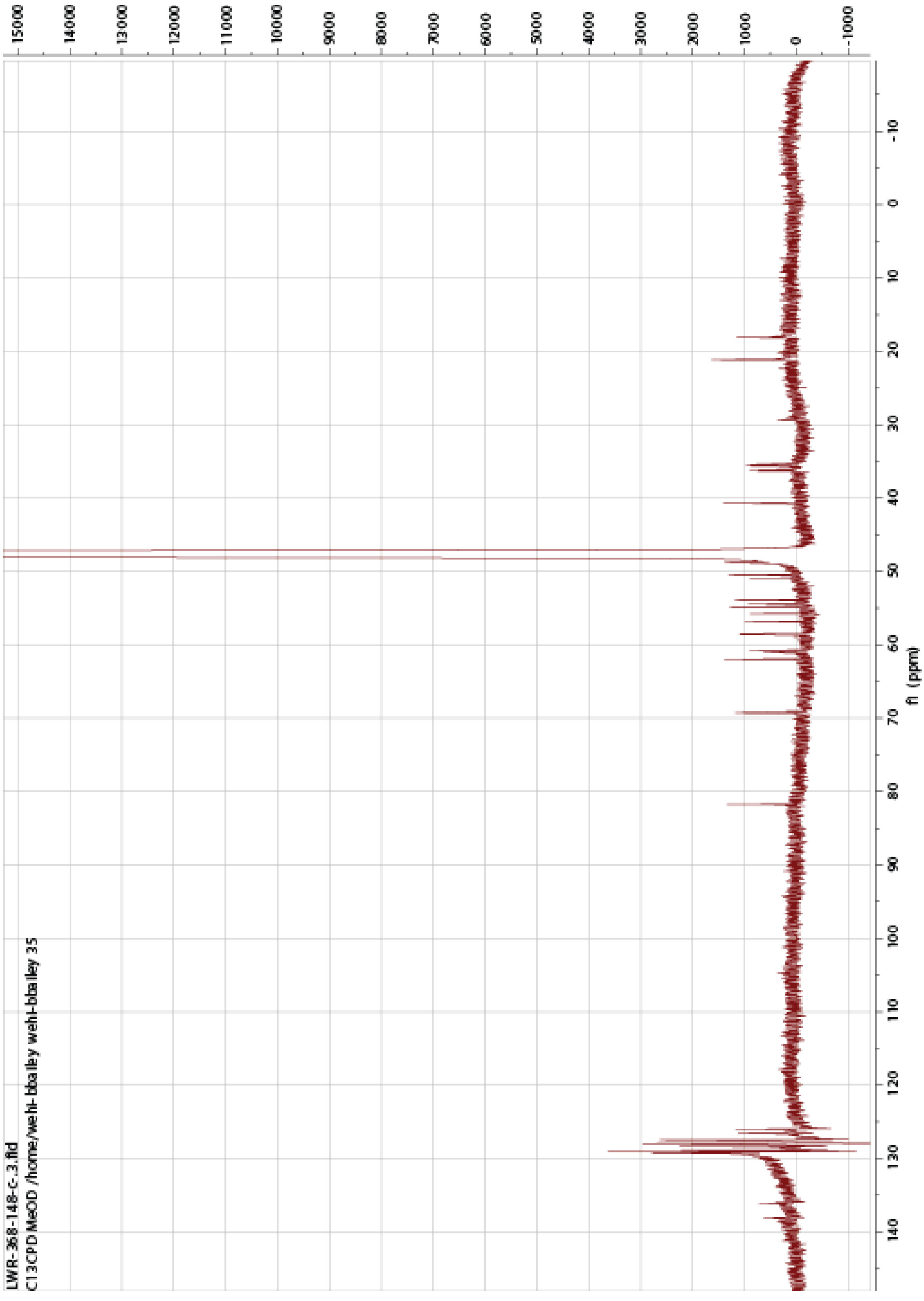
LWR-368-148-c-3.fid
C13CPD MeOD /home/wehi-bbailey wehi-bbailey 35
f1 (ppm)

27 H NMR

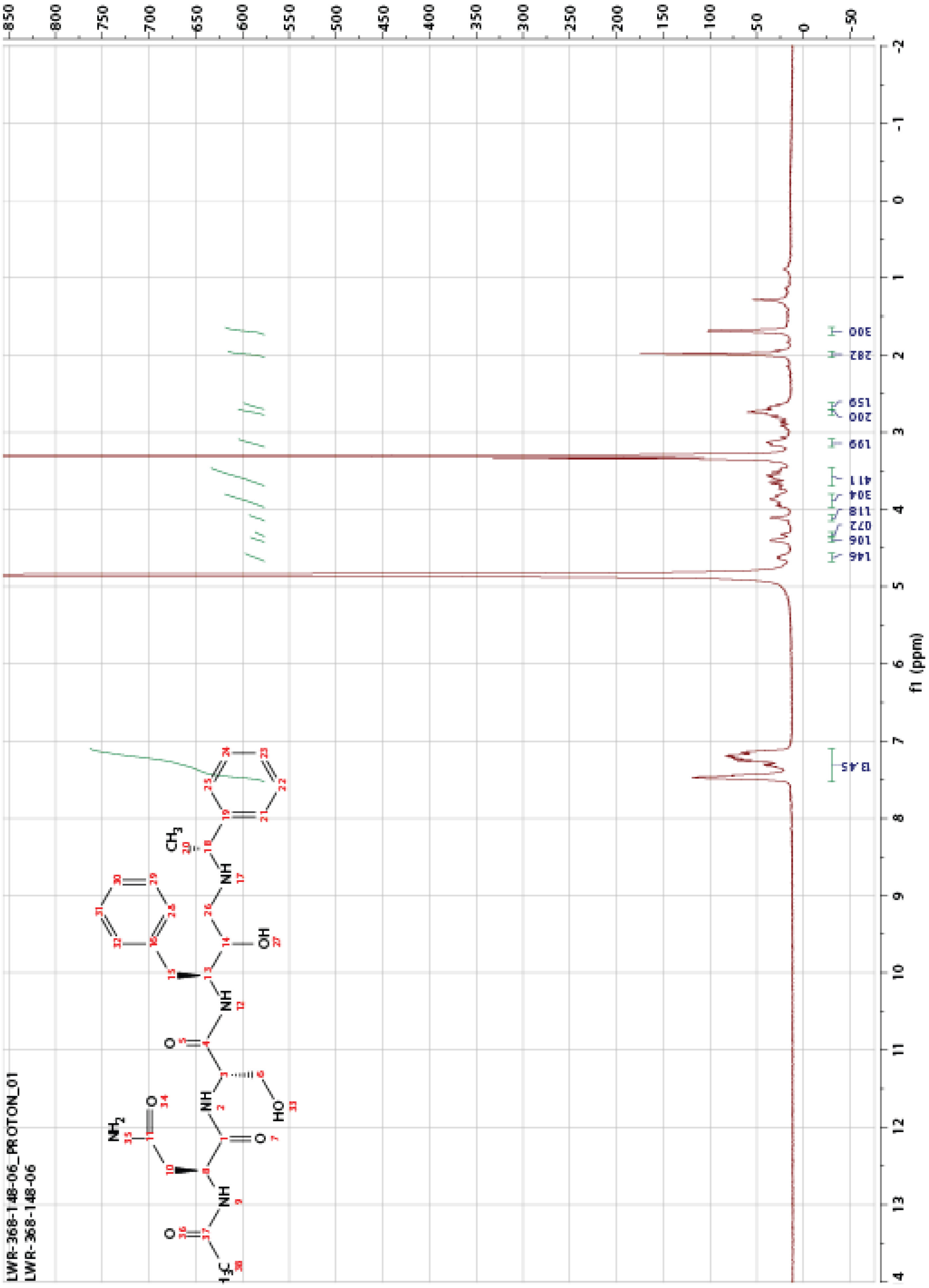

27 LCMS

Current Chromatogram(s)

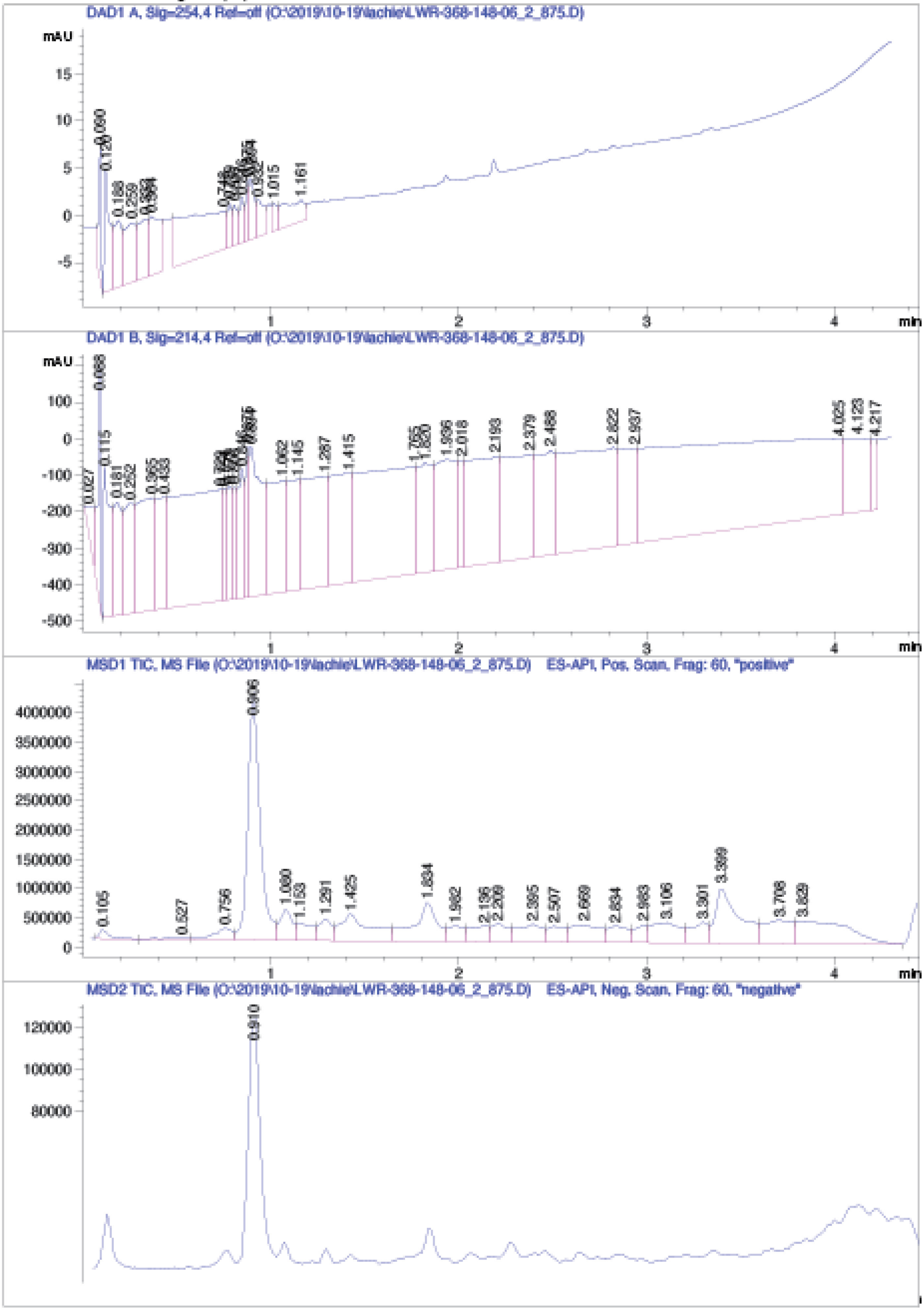

MS Spectrum

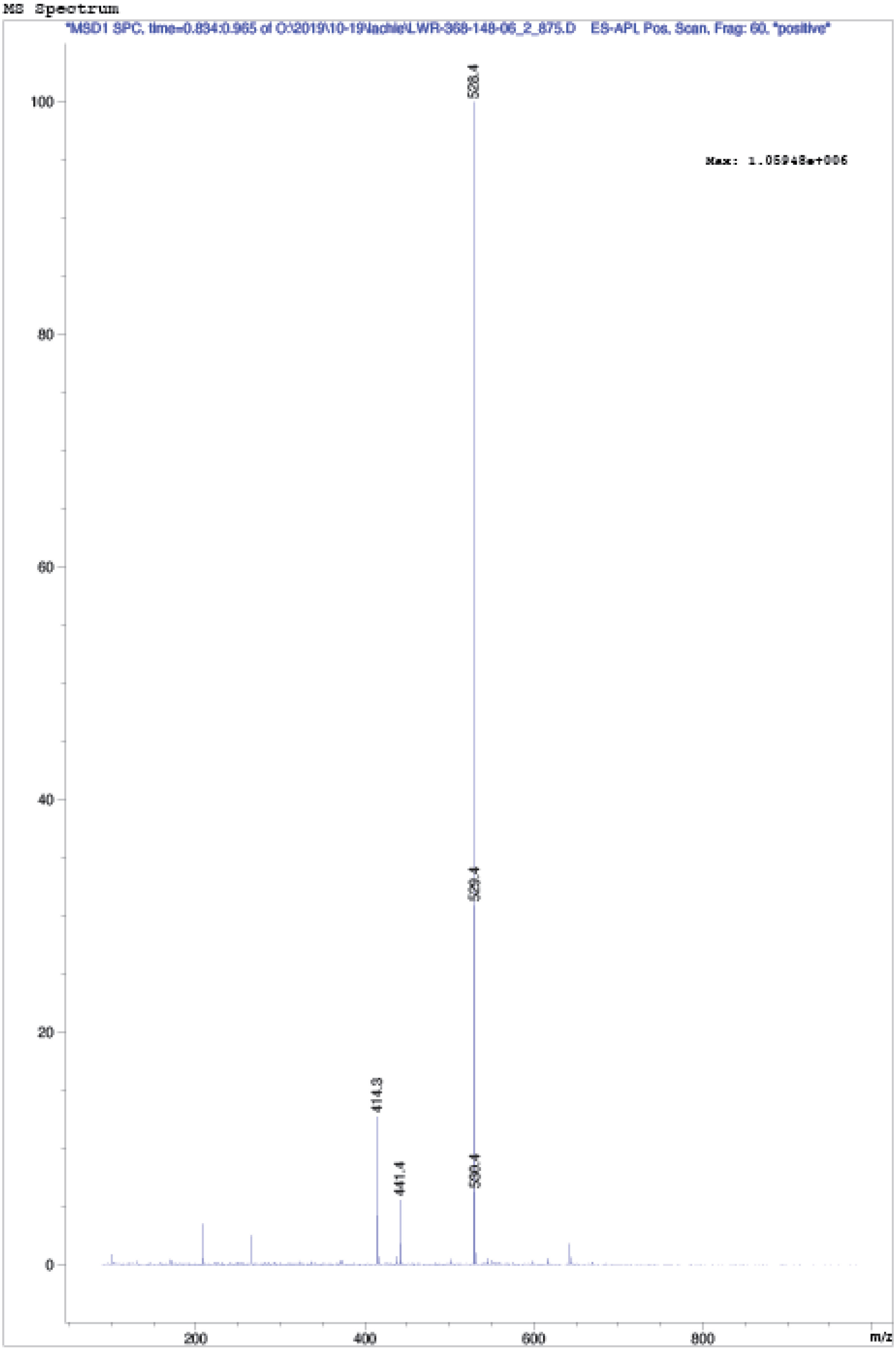

*MSD1 SPC, time=0.834:0.965 of O:\2019\10-19\lachie\LWR-368-148-06_2_875.D ES-API, Pos, Scan, Frag: 60, "positive"
Max: 1.05948e+006
528.4
529.4
414.3
441.4
530.4
100
80
60
40
20
0
200
400
600
800
m/z

28 C NMR

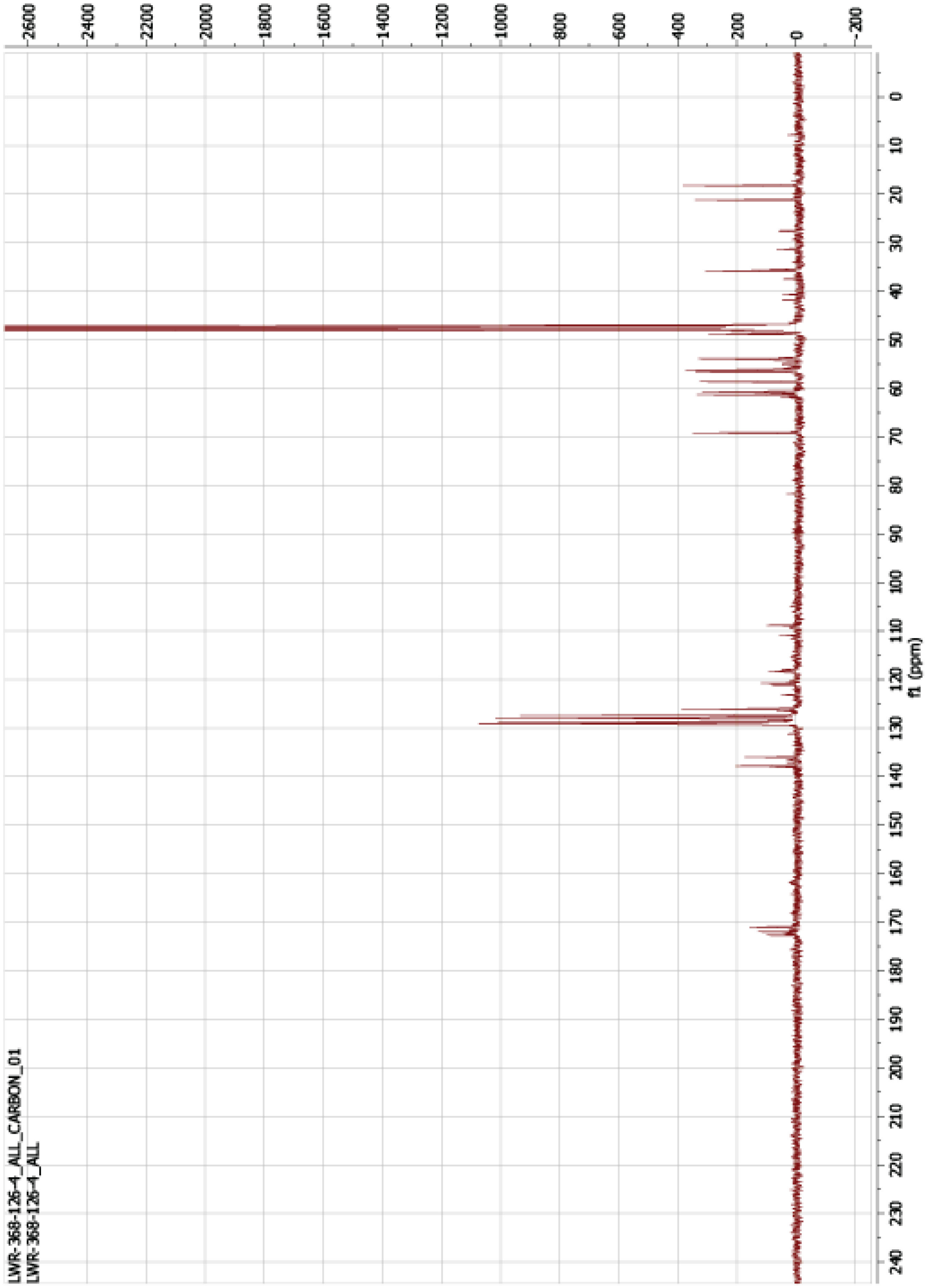

28 H NMR

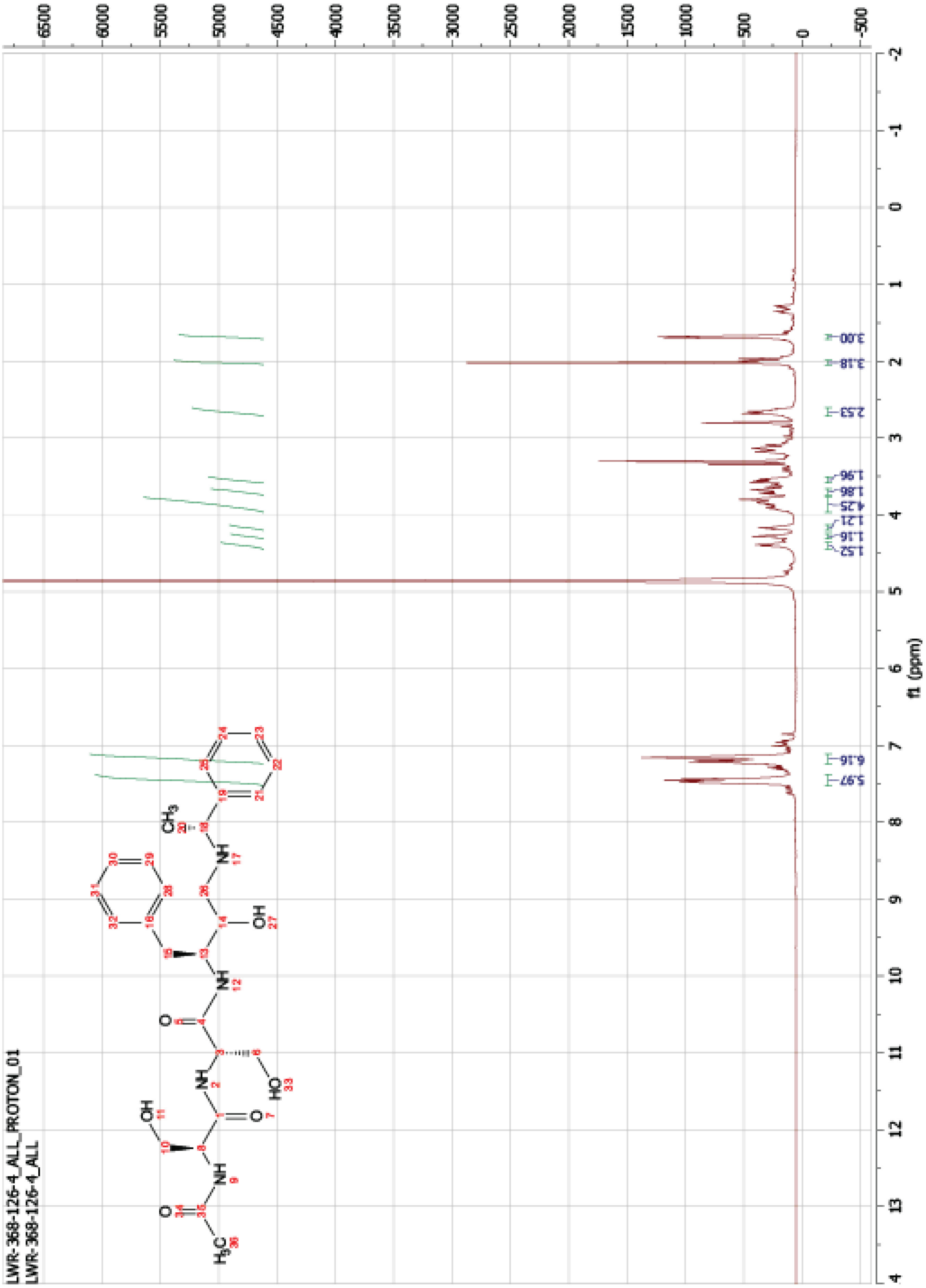
LWR-368-126-4_ALL_PROTON_01
LWR-368-126-4_ALL
f1 (ppm)
3.00
3.18
2.53
1.96
1.86
4.25
1.21
1.16
1.52
6.16
5.97

28 LCMS

Current Chromatogram(s)

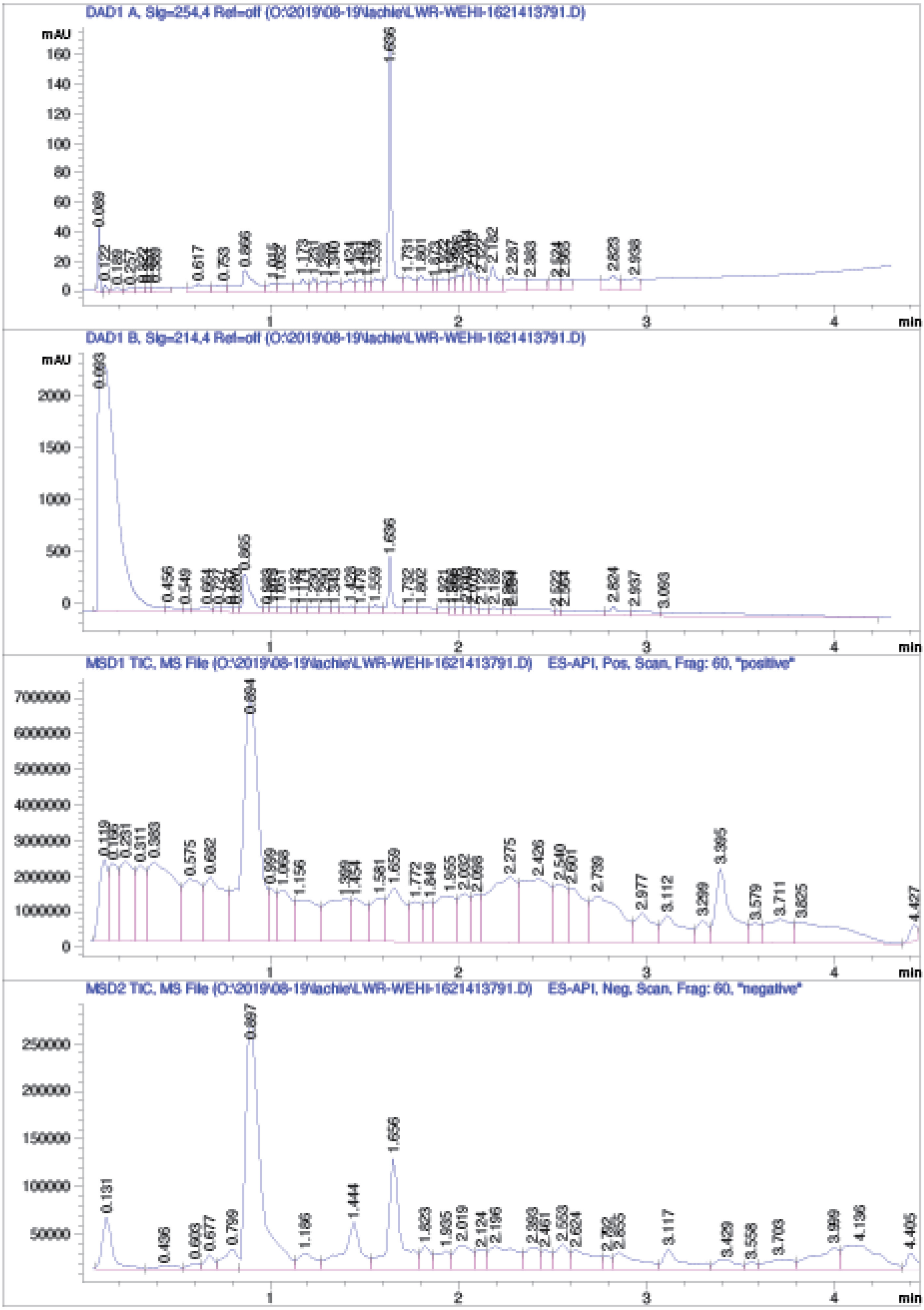

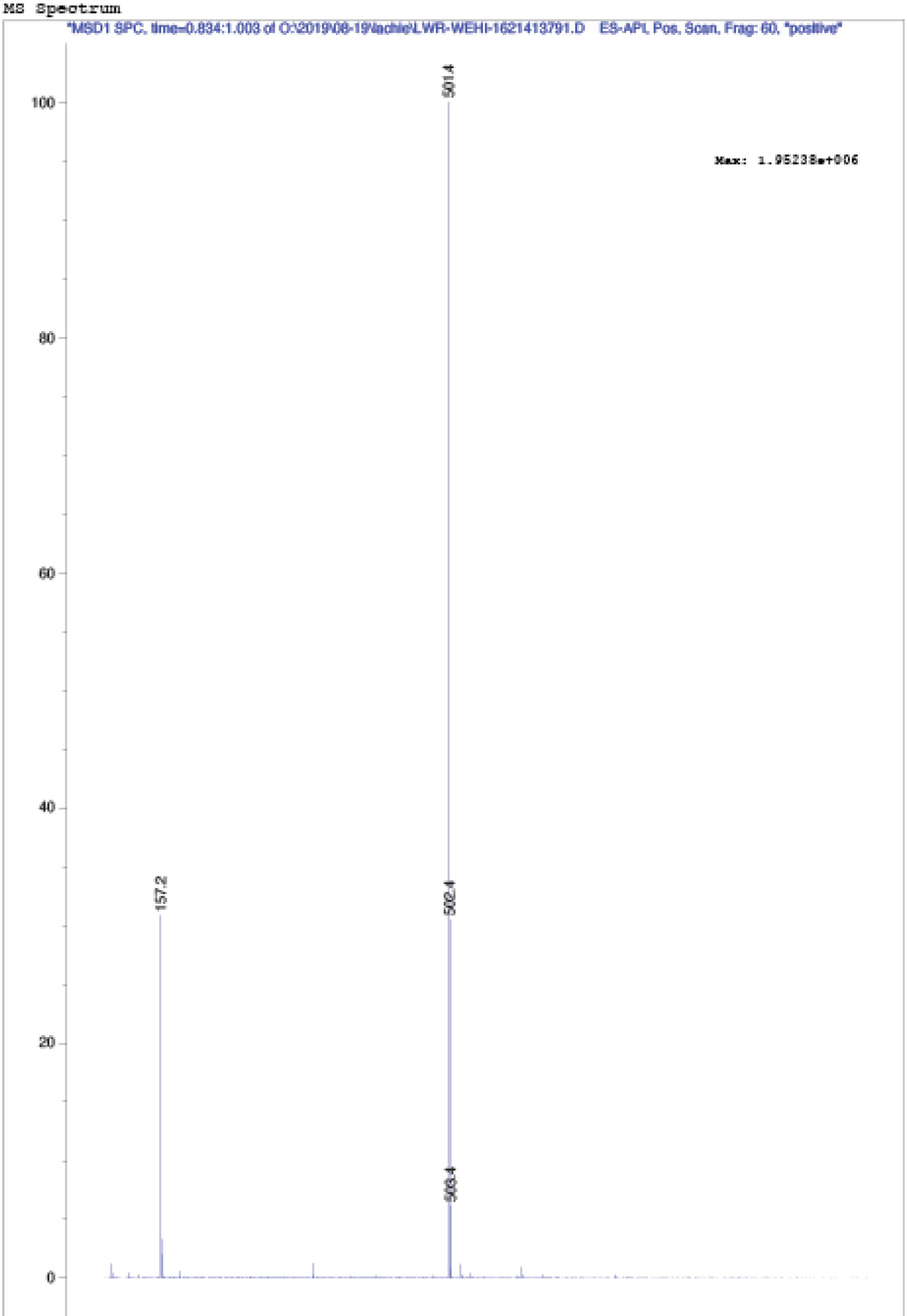
MS Spectrum
*MSD1 SPC, time=0.834:1.003 of O:\2019\08-19\lachie\LWR-WEHI-1621413791.D ES-API, Pos, Scan, Frag: 60, "positive"
Max: 1.95238e+006
501.4
157.2
502.4
503.4
100
80
60
40
20
0
200
400
600
800
m/z

34 C NMR

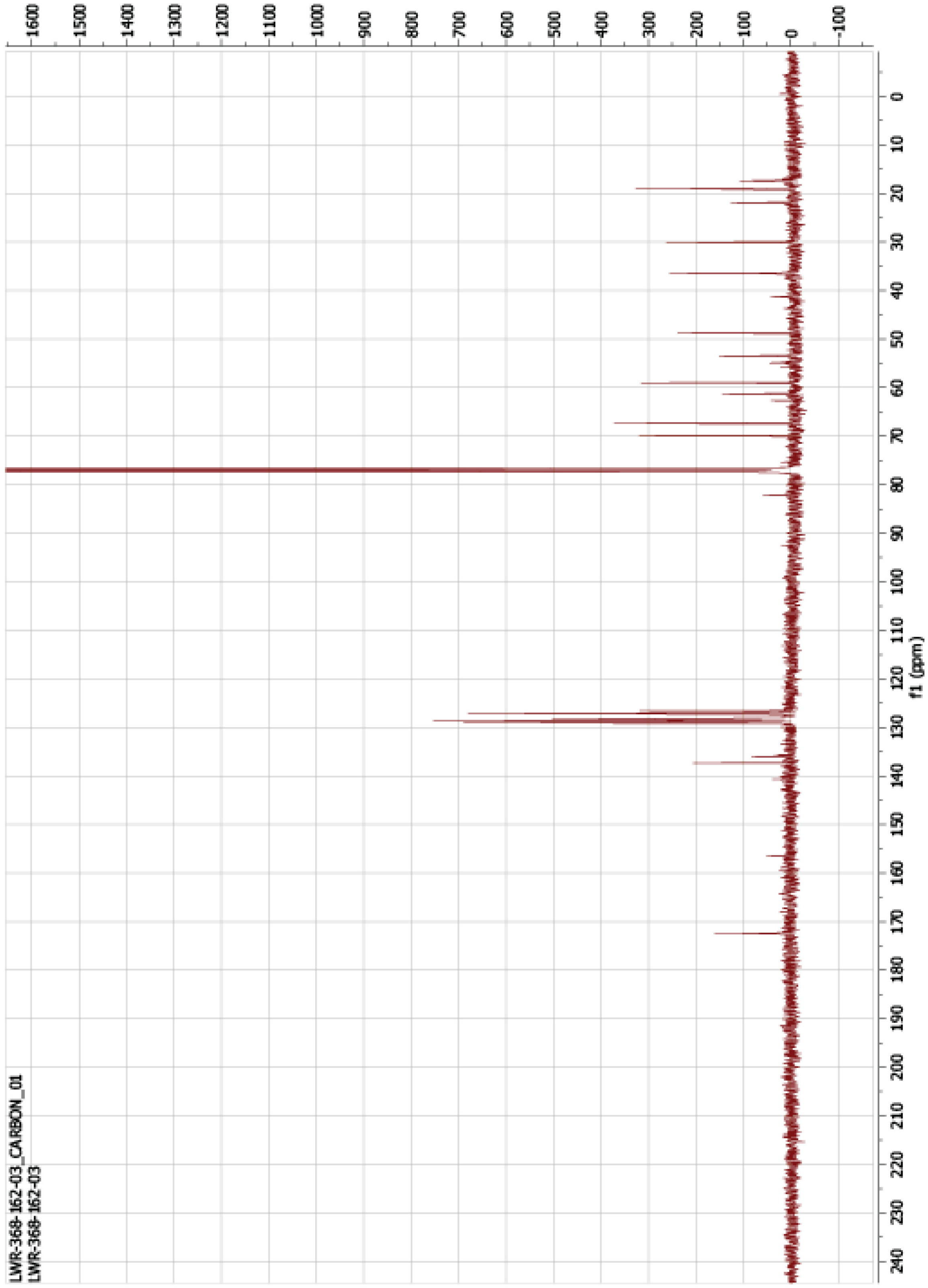

34 H NMR

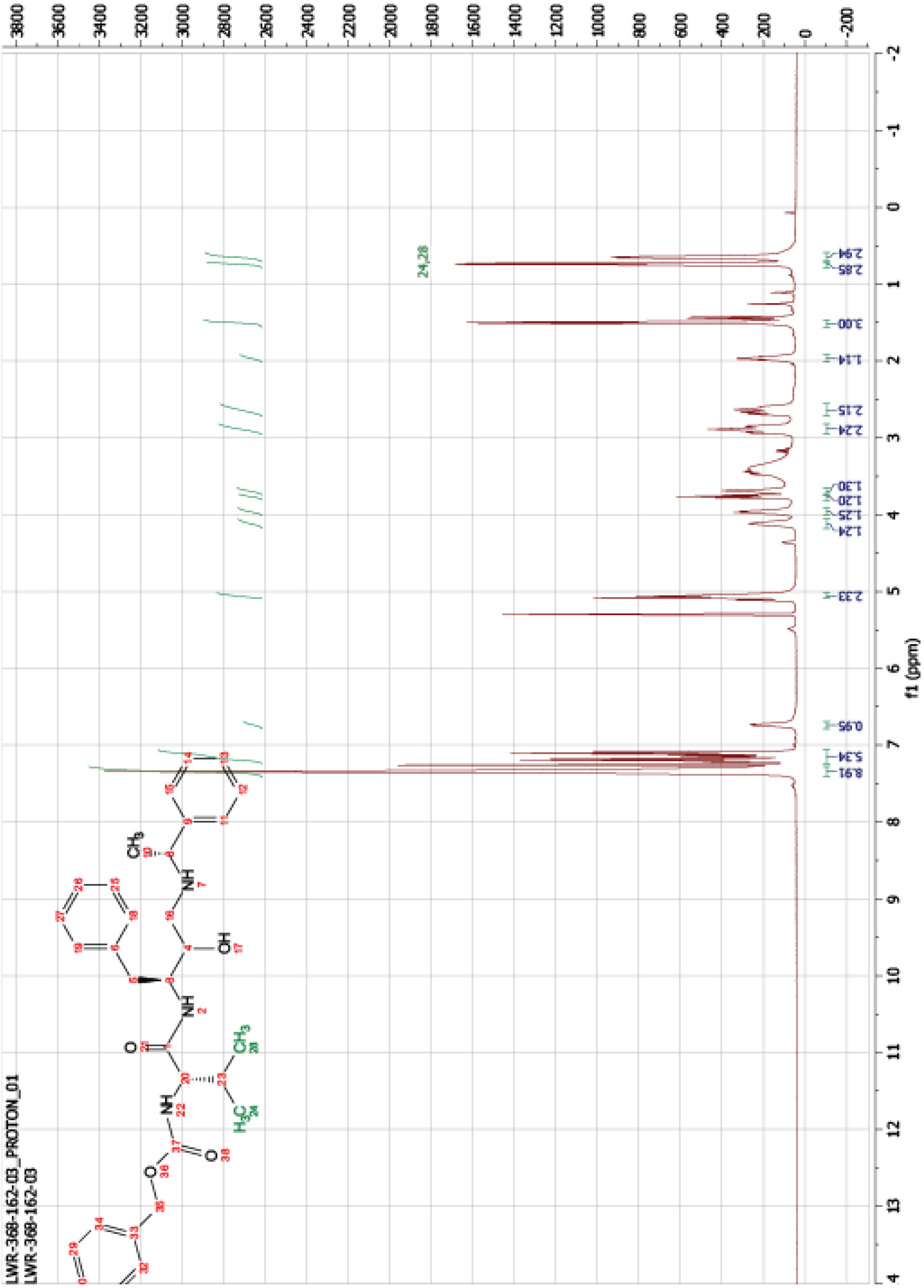

34 LCMS

Current Chromatogram(s)

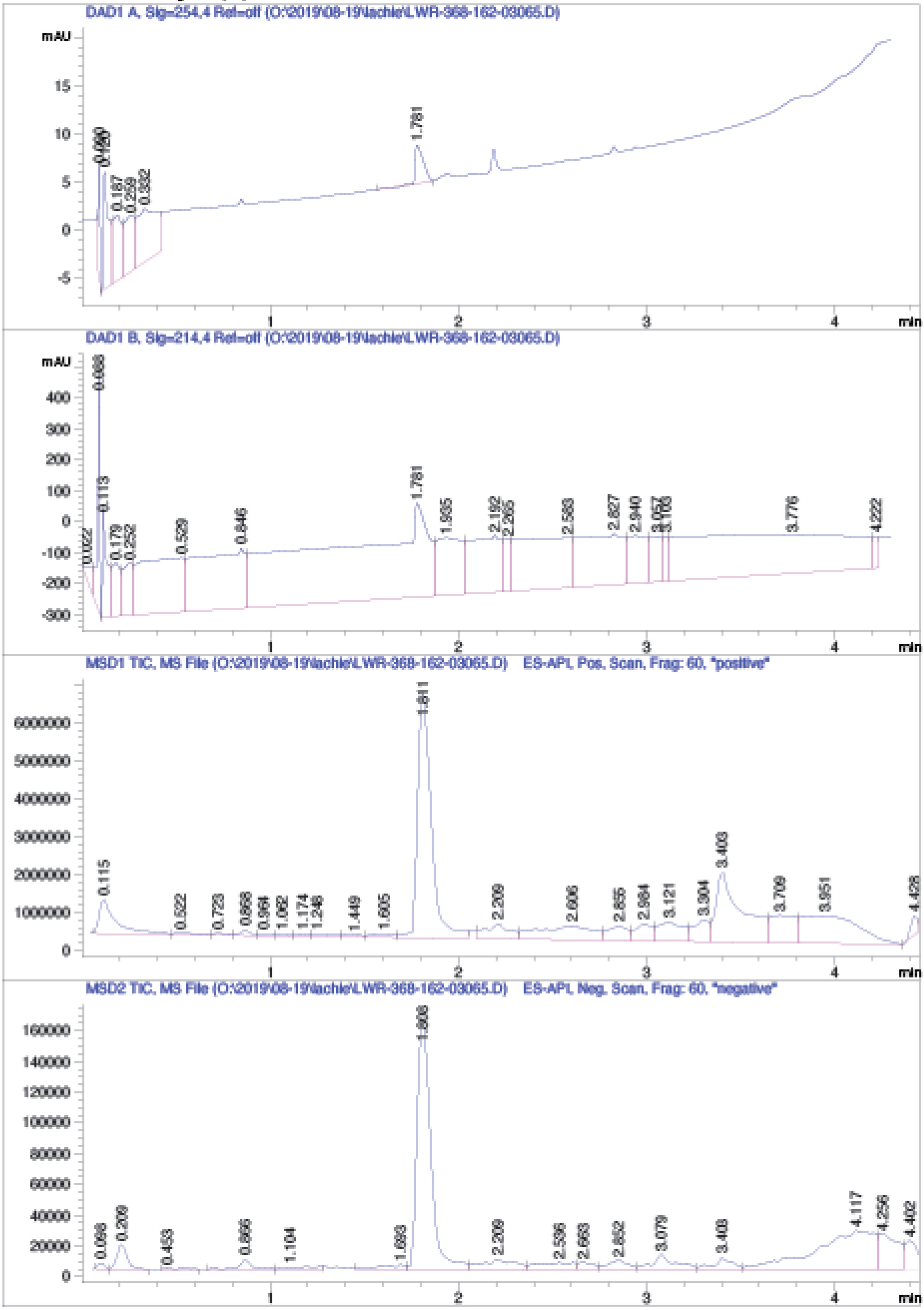

MS Spectrum

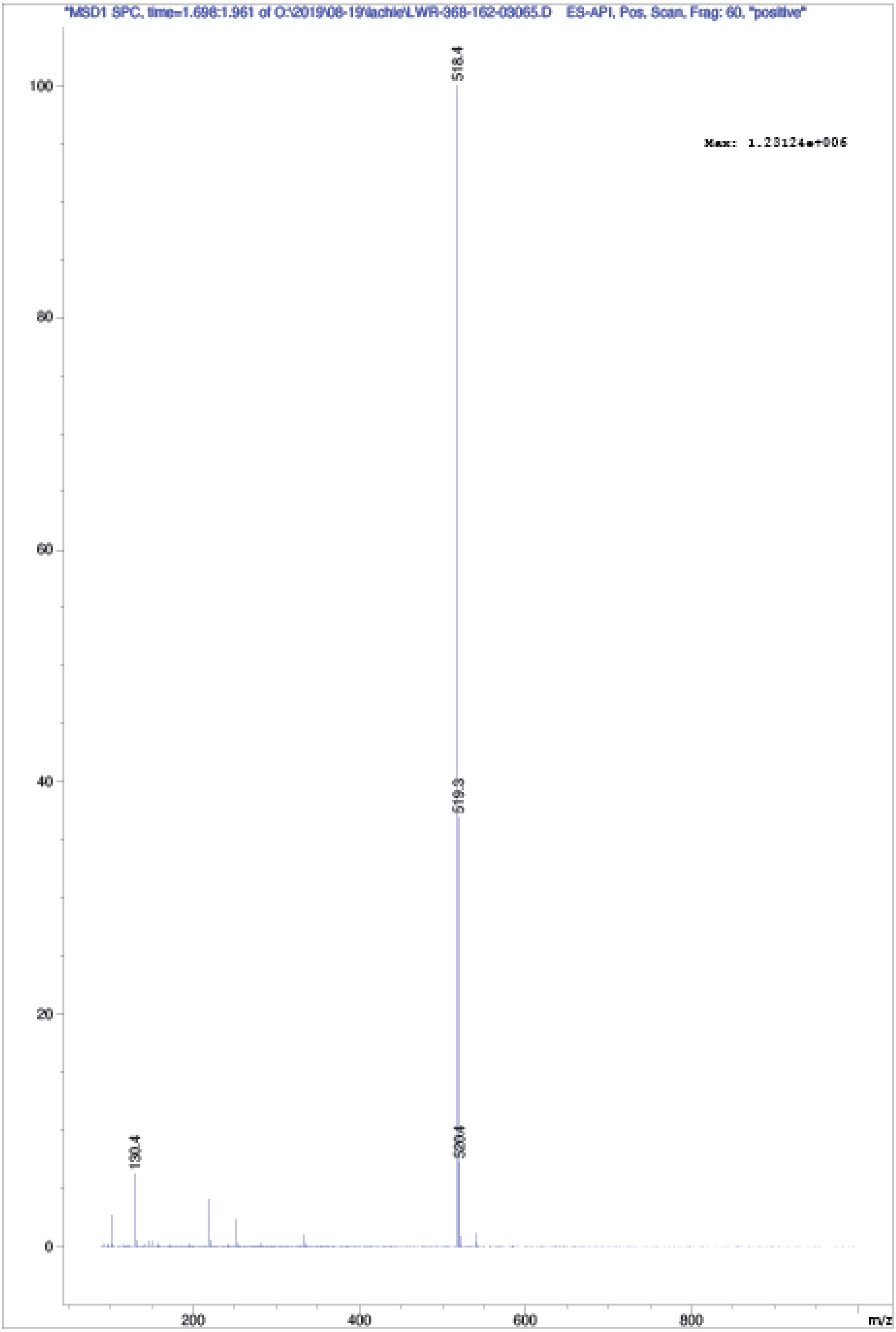

*MSD1 SPC, time=1.698:1.961 of O:\2019\08-19\lachie\LWR-368-162-03065.D ES-API, Pos, Scan, Frag: 60, "positive"
Max: 1.23124e+006
518.4
519.3
520.4
130.4
100
80
60
40
20
0
200
400
600
800
m/z

35 C NMR

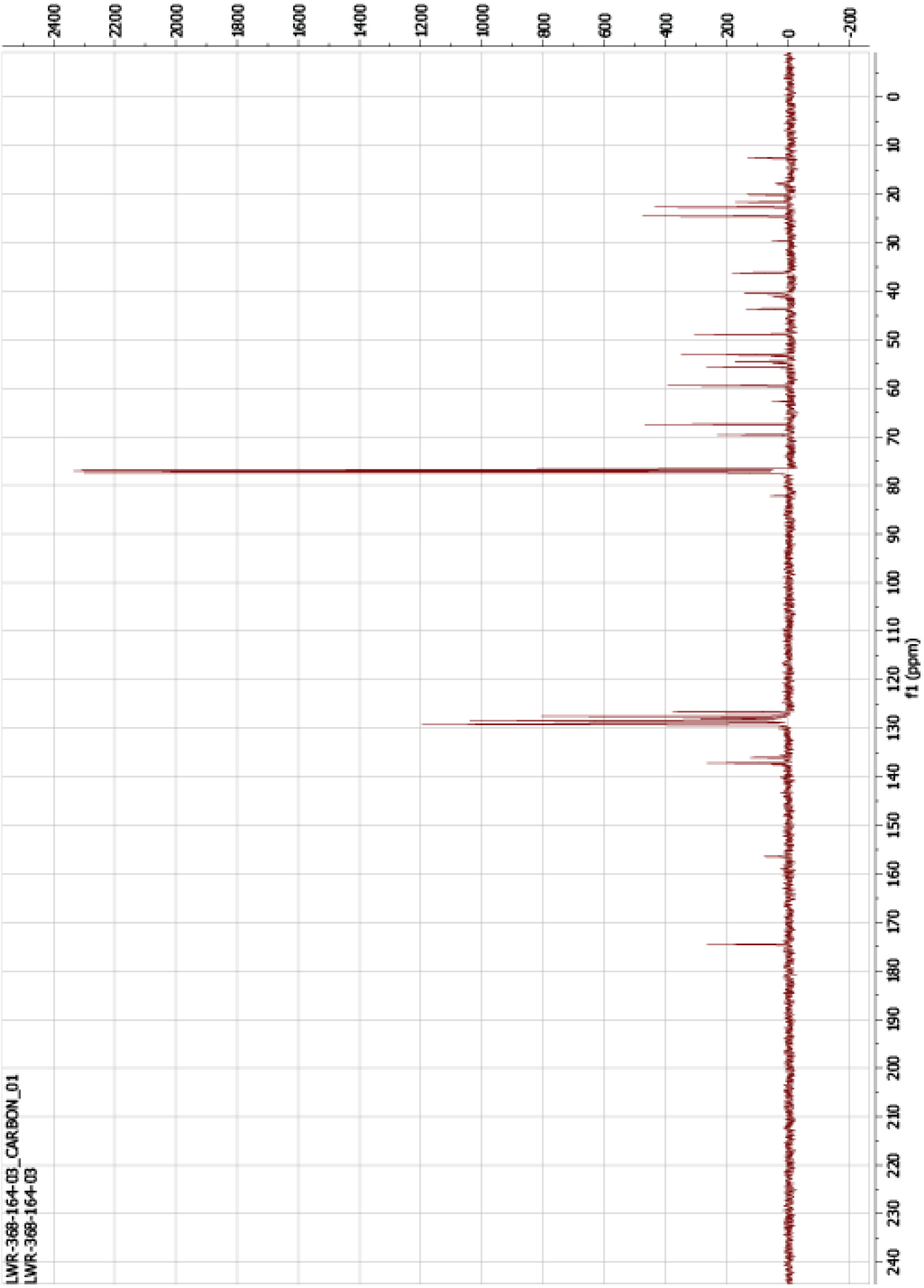

35 H NMR

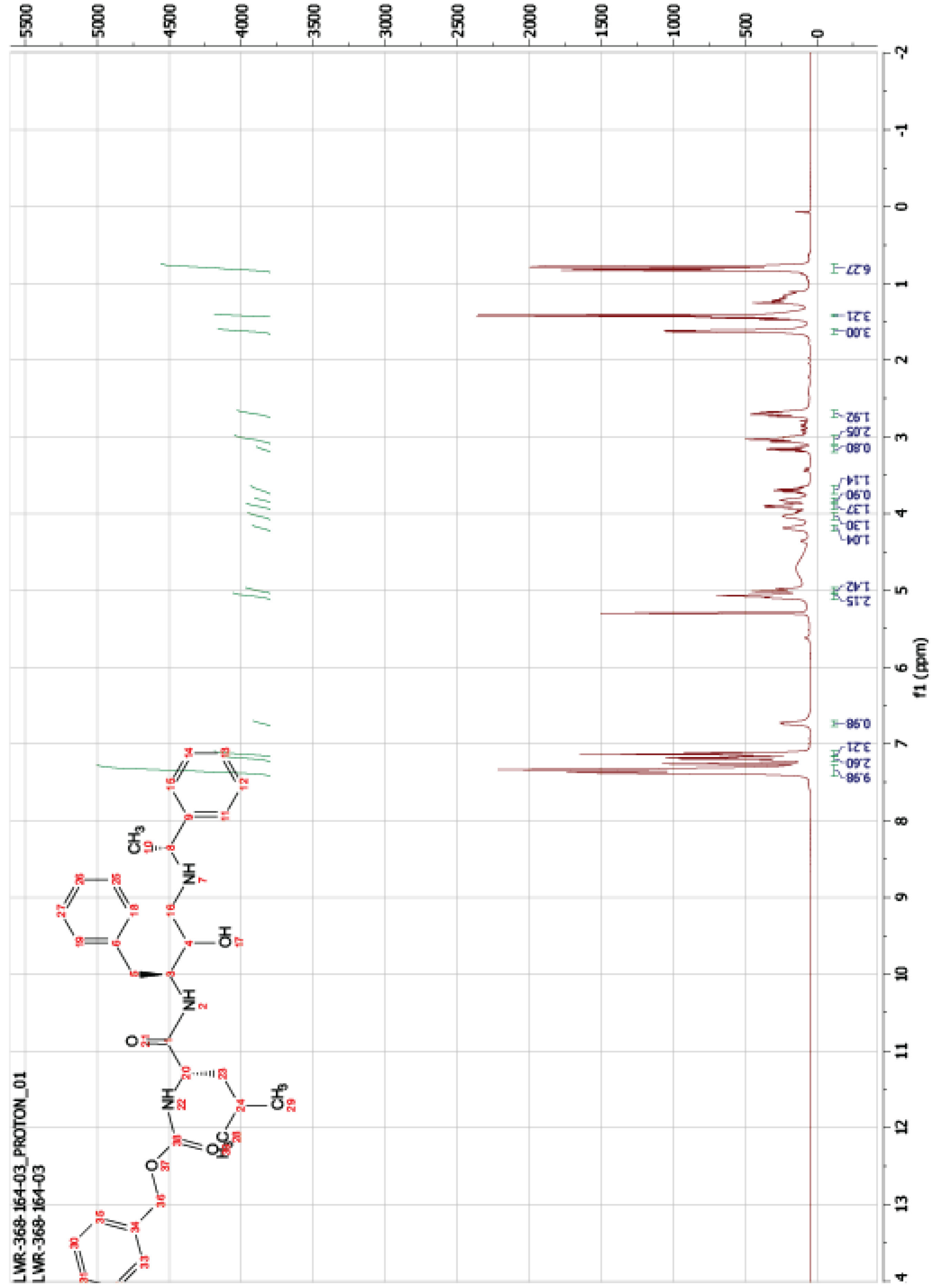

35 LCMS

Current Chromatogram(s)

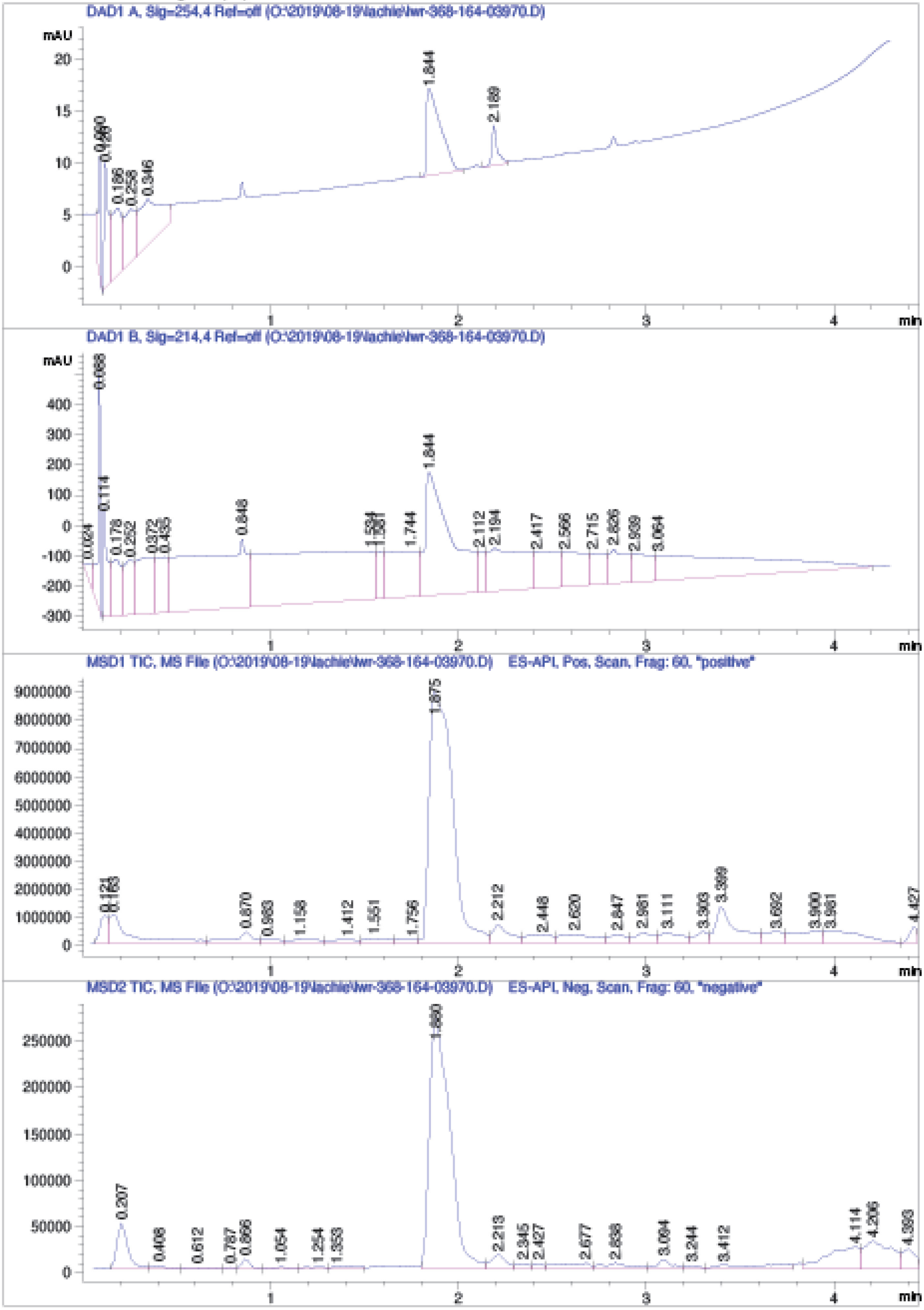

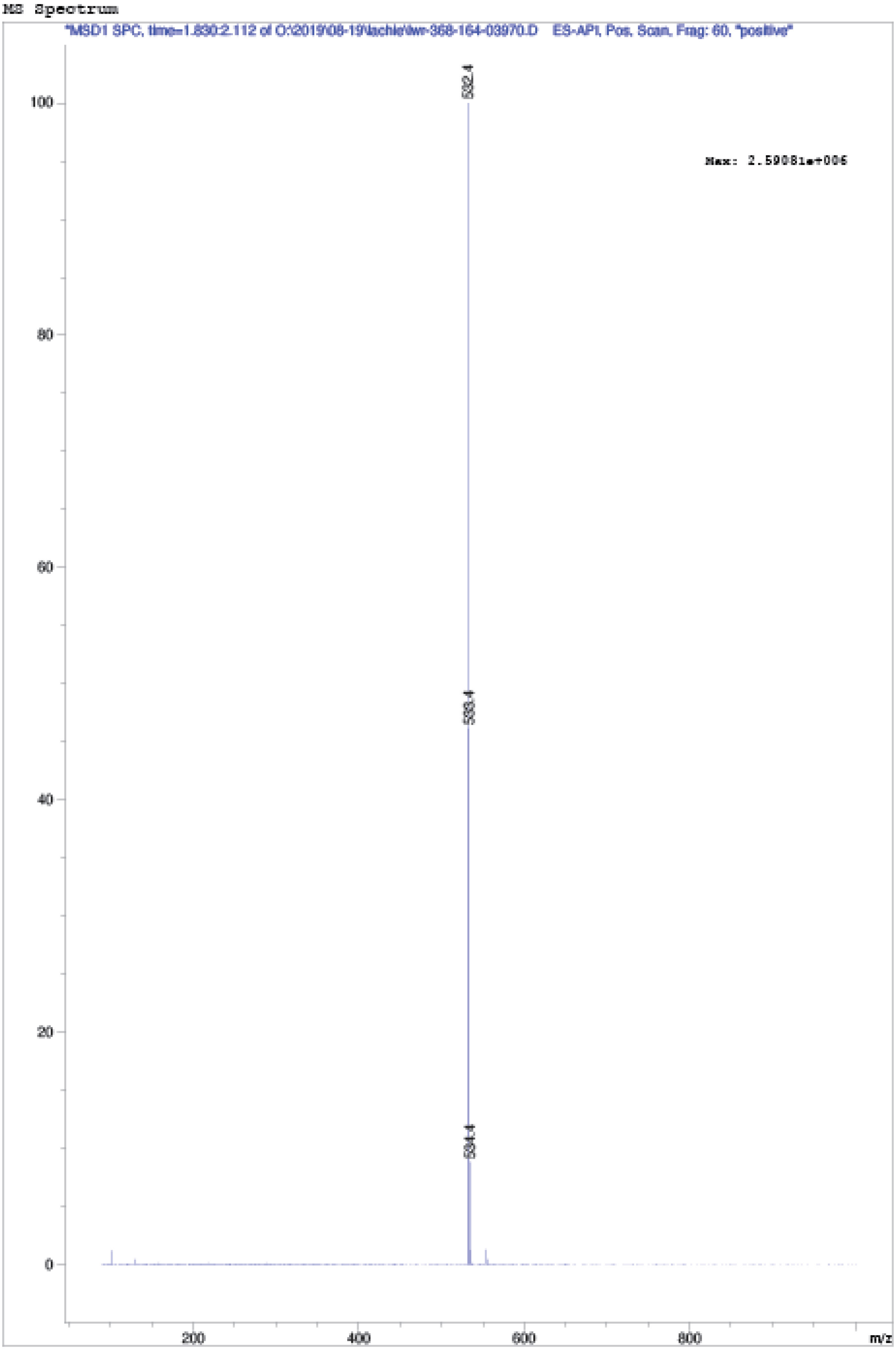
MS Spectrum
*MSD1 SPC, time=1.830:2.112 of O:\2019\08-19\achie\lwr-368-164-03970.D ES-API, Pos, Scan, Frag: 60, "positive"
Max: 2.59081e+006
532.4
533.4
534.4
100
80
60
40
20
0
200
400
600
800
m/z

36 C NMR

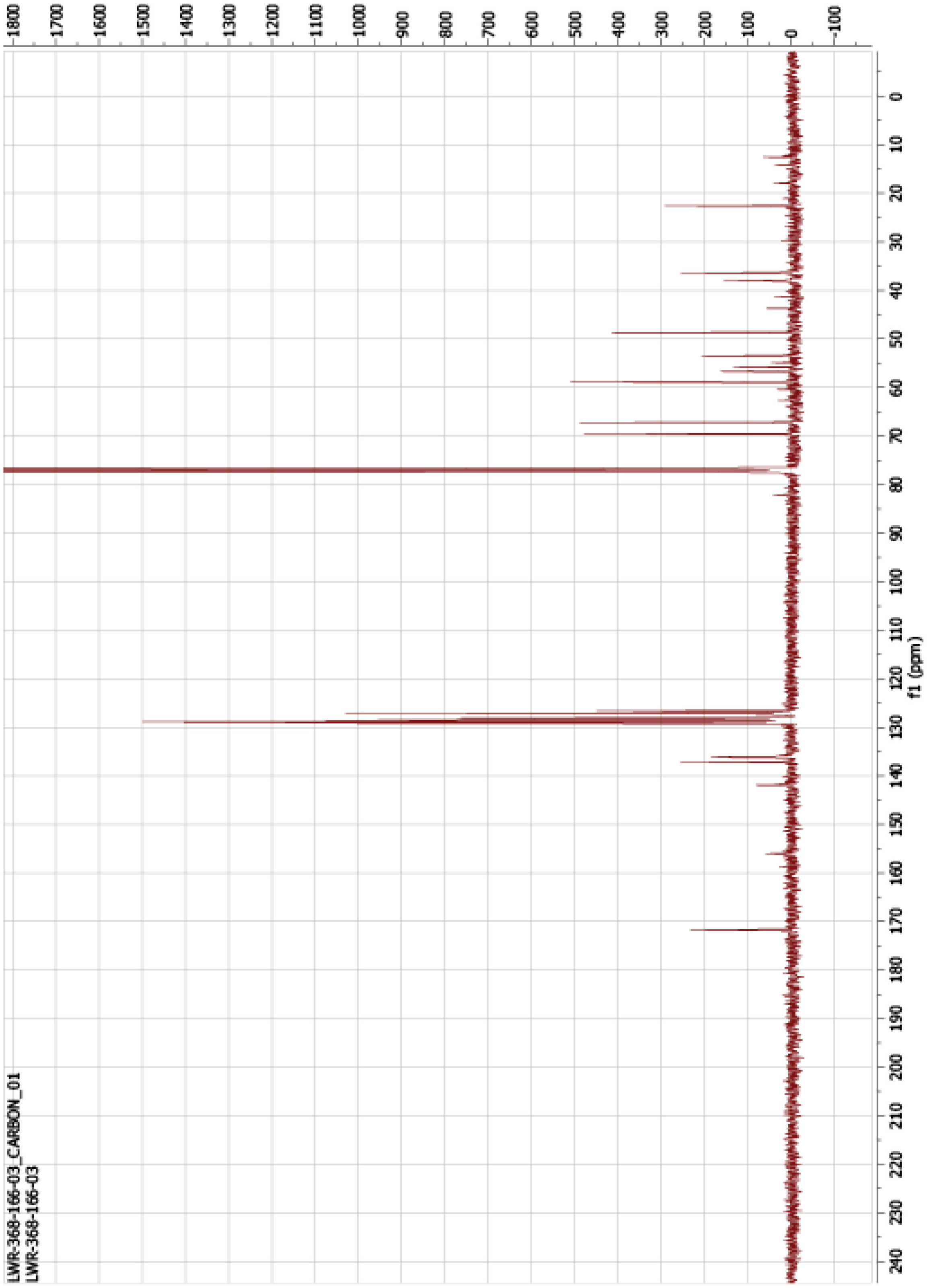

36 H NMR

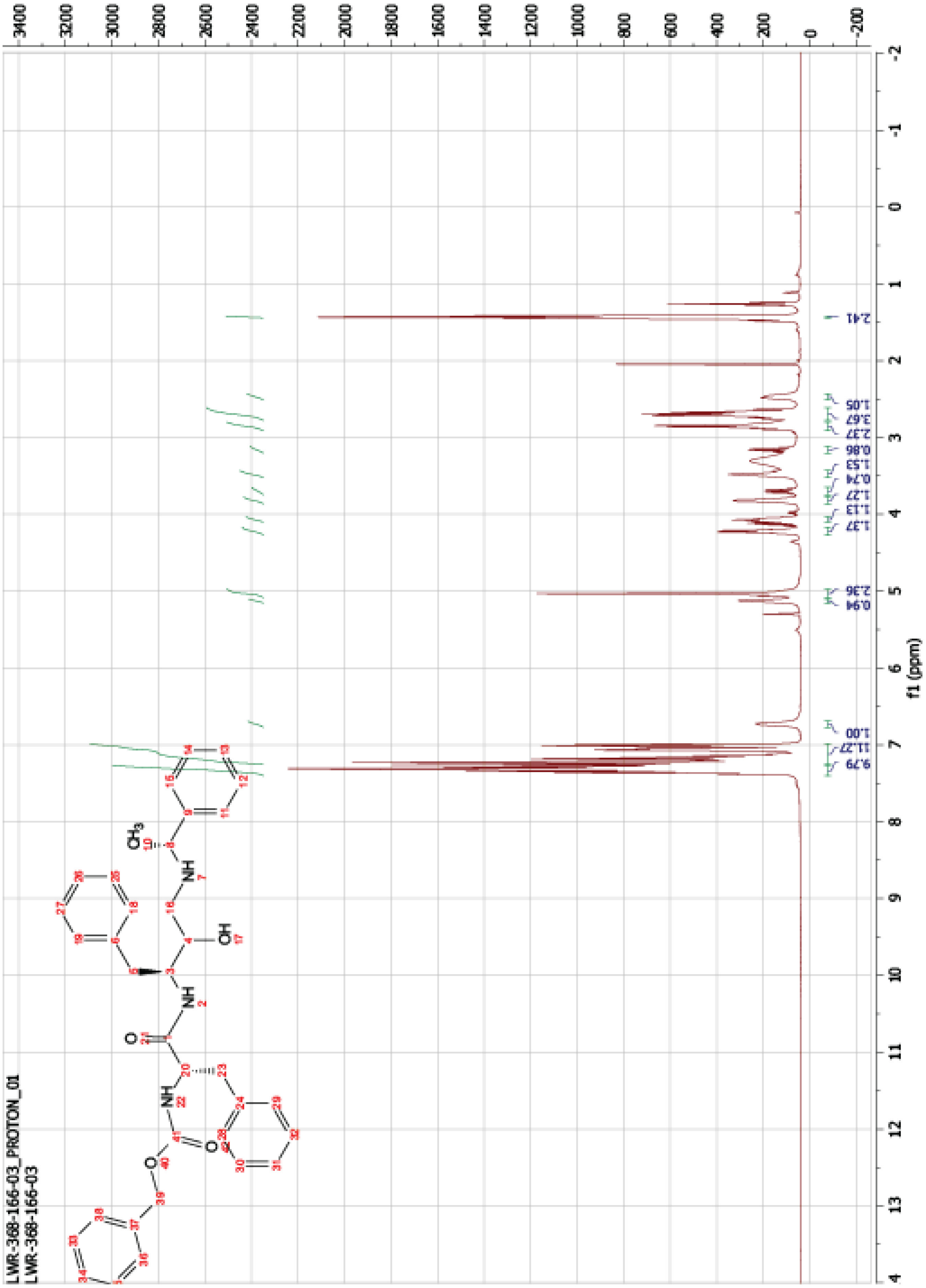

36 LCMS

Current Chromatogram(s)

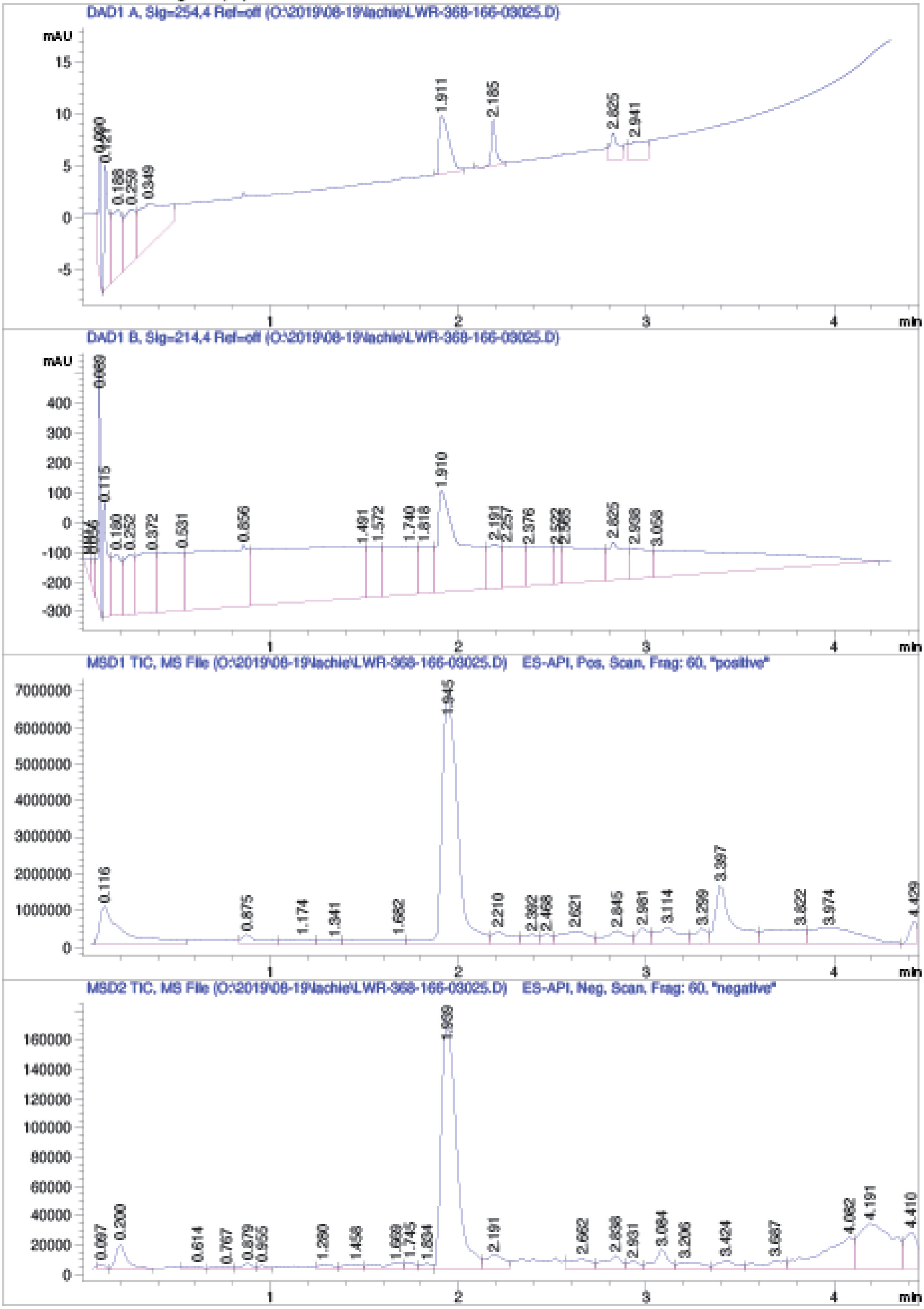

MS Spectrum

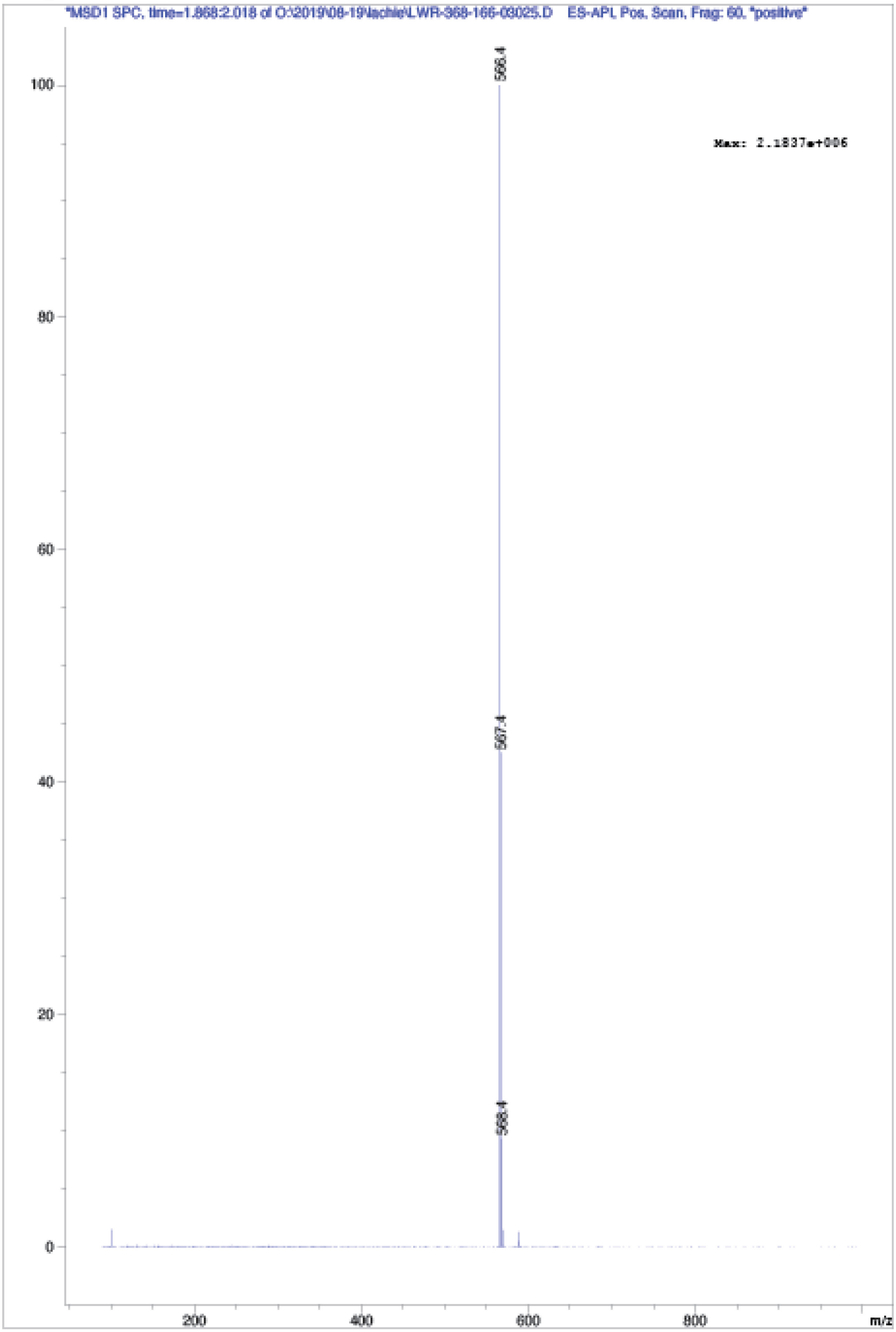

*MSD1 SPC, time=1.868:2.018 of O:\2019\08-19\achie\LWR-368-166-03025.D ES-API, Pos, Scan, Frag: 60, "positive"
566.4
Max: 2.1837e+006
567.4
568.4
100
80
60
40
20
0
200
400
600
800
m/z

37 C NMR

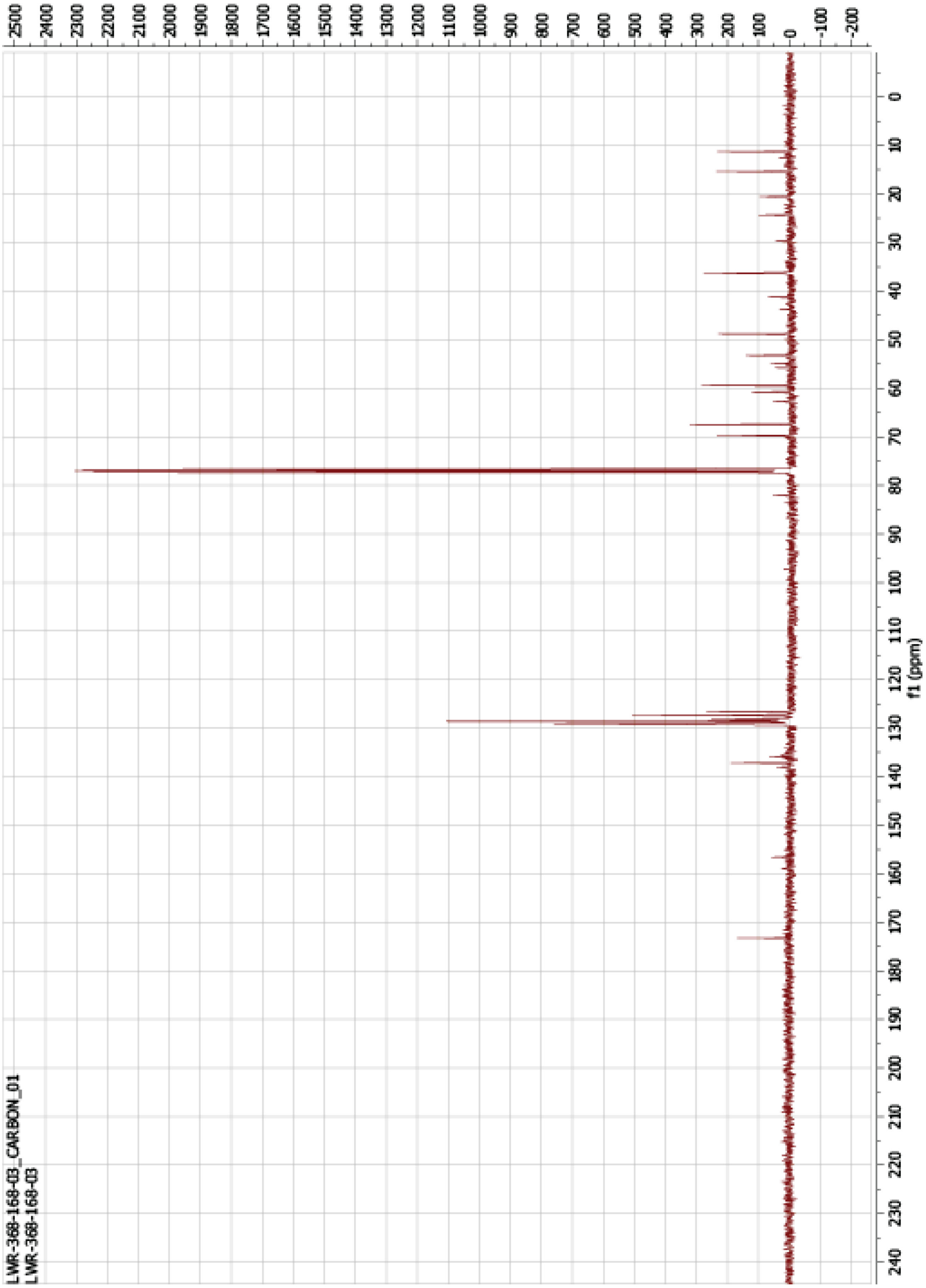

37 H NMR

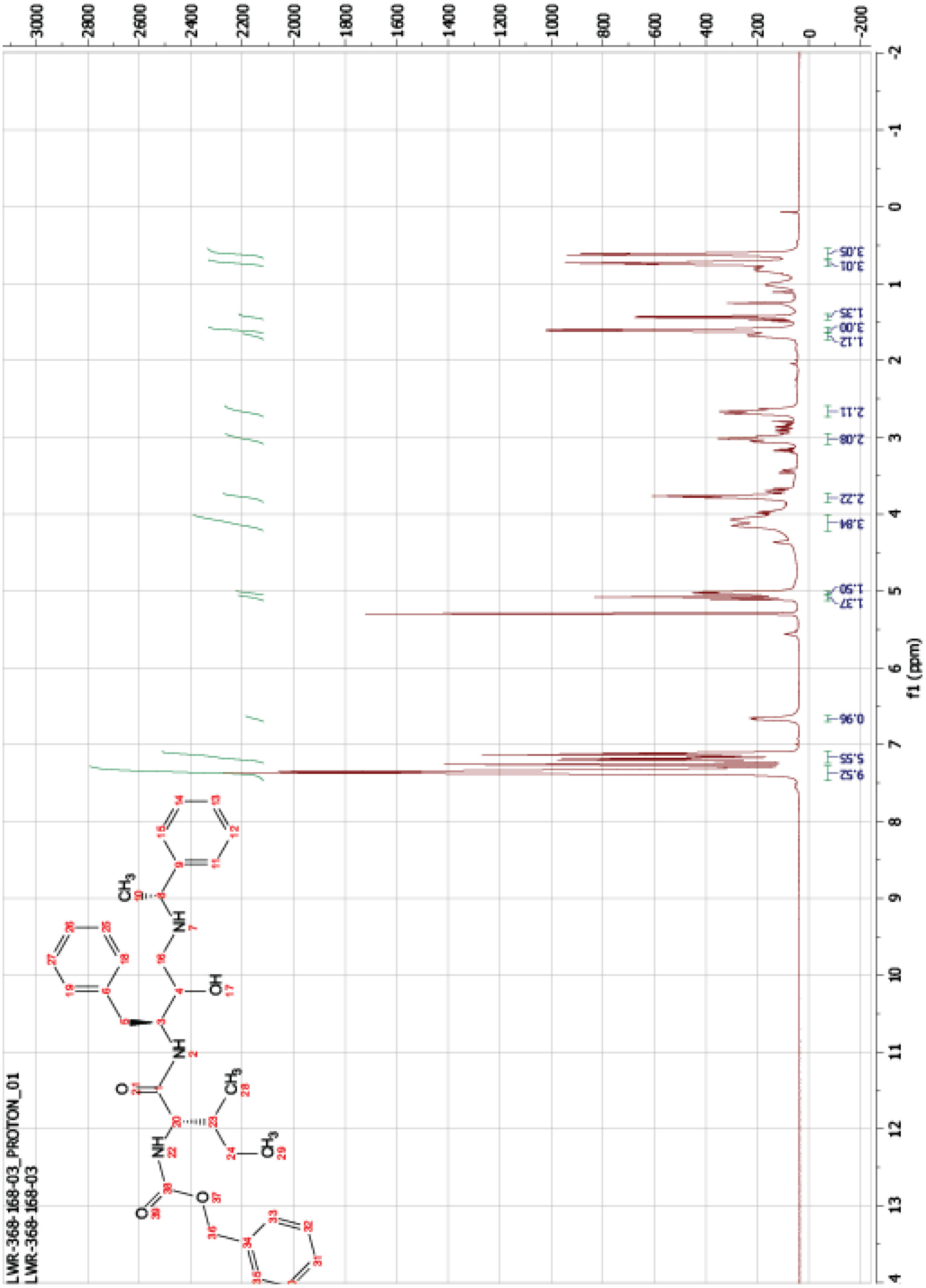

LWR-368-168-03_PROTON_01
LWR-368-168-03
f1 (ppm)
3.05
3.01
1.35
3.00
1.12
2.11
2.08
2.22
3.84
1.50
1.37
0.96
5.55
9.52

37 LCMS

Current Chromatogram(s)

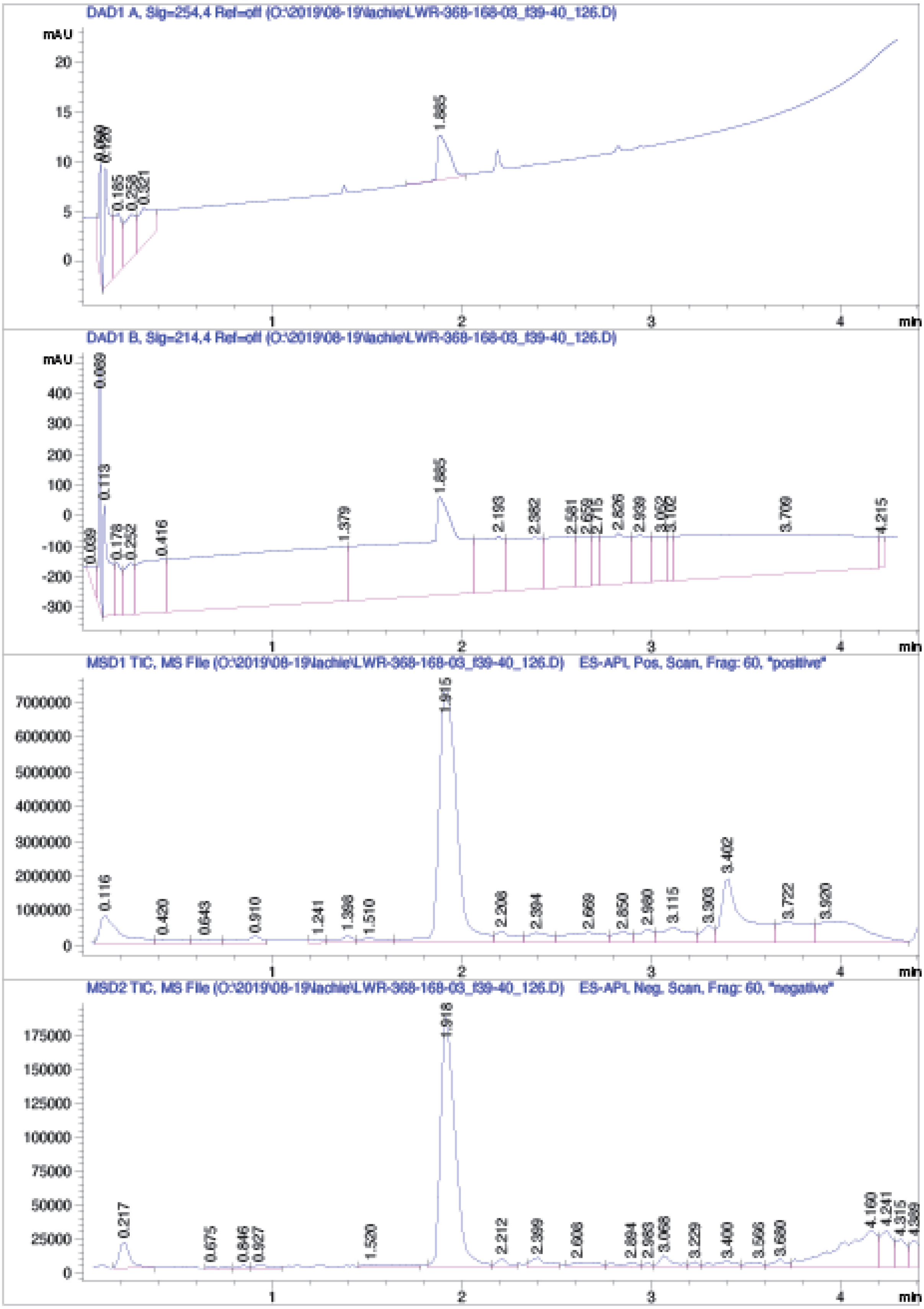

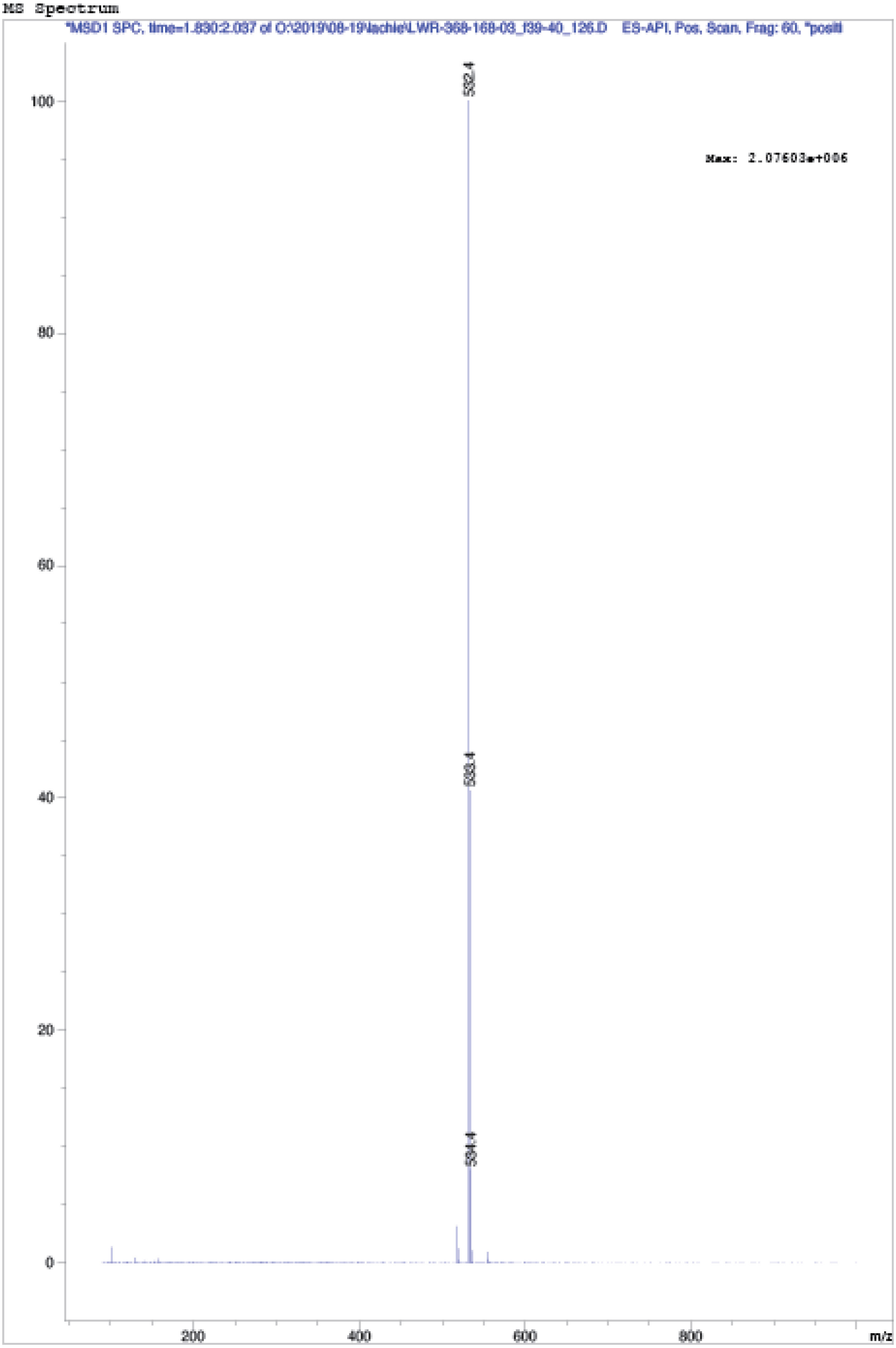
MS Spectrum
*MSD1 SPC, time=1.830:2.037 of O:\2019\08-19\achie\LWR-368-168-03_f39-40_126.D ES-API, Pos, Scan, Frag: 60, *positi
532.4
533.4
534.4
Max: 2.07603e+006
100
80
60
40
20
0
200
400
600
800
m/z

38 C NMR

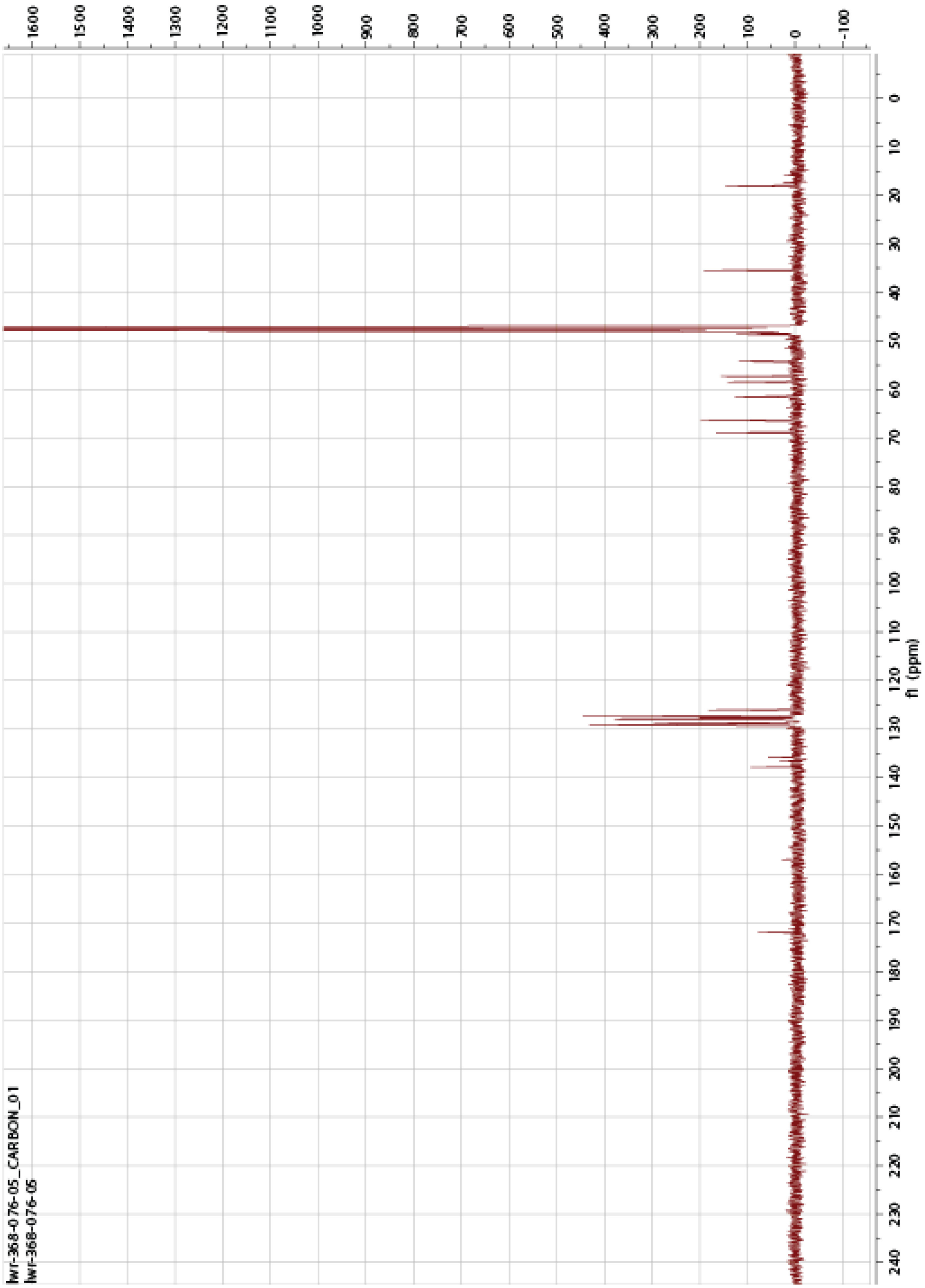

38 H NMR

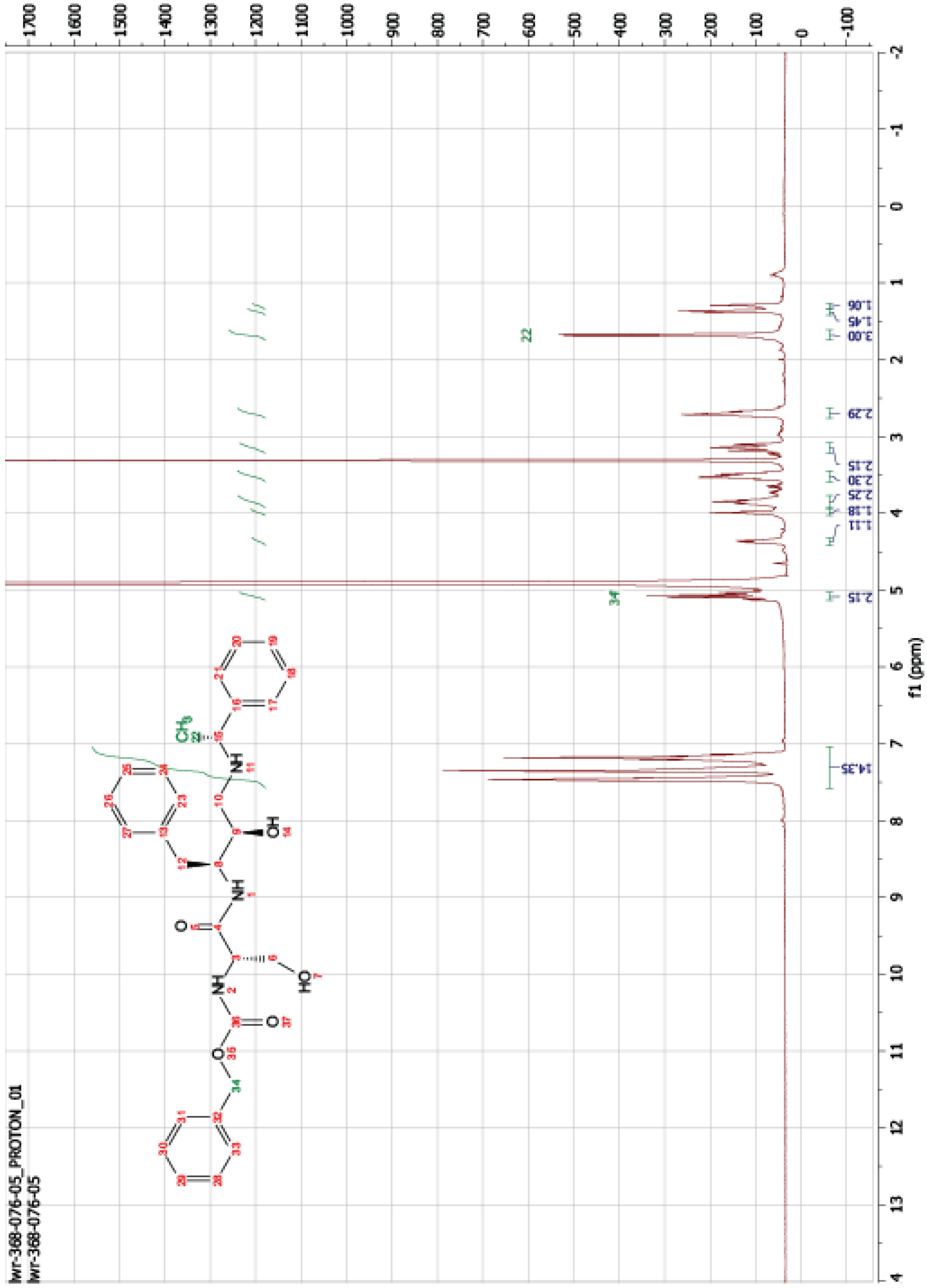

38 LCMS

Current Chromatogram(s)

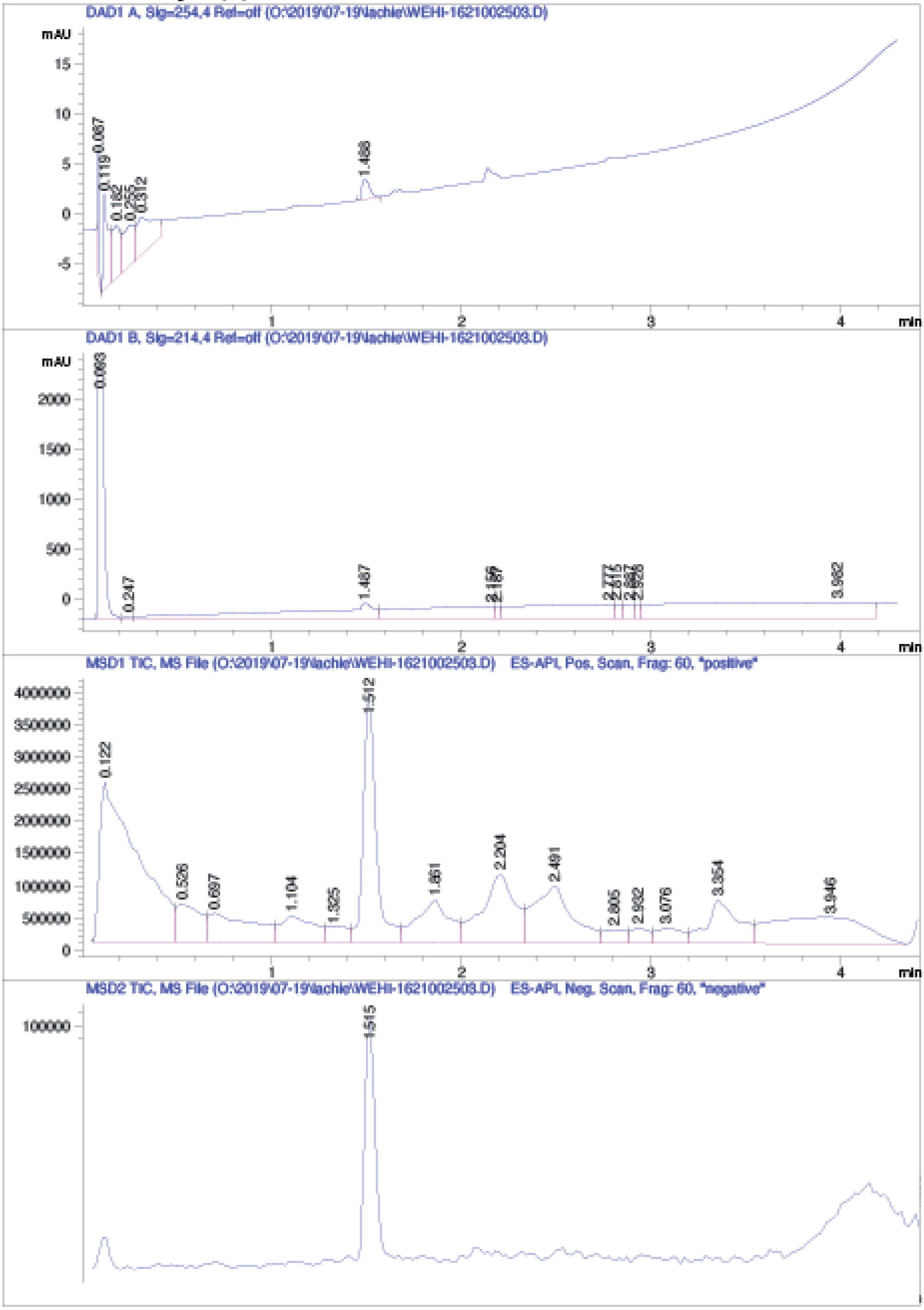

MS Spectrum

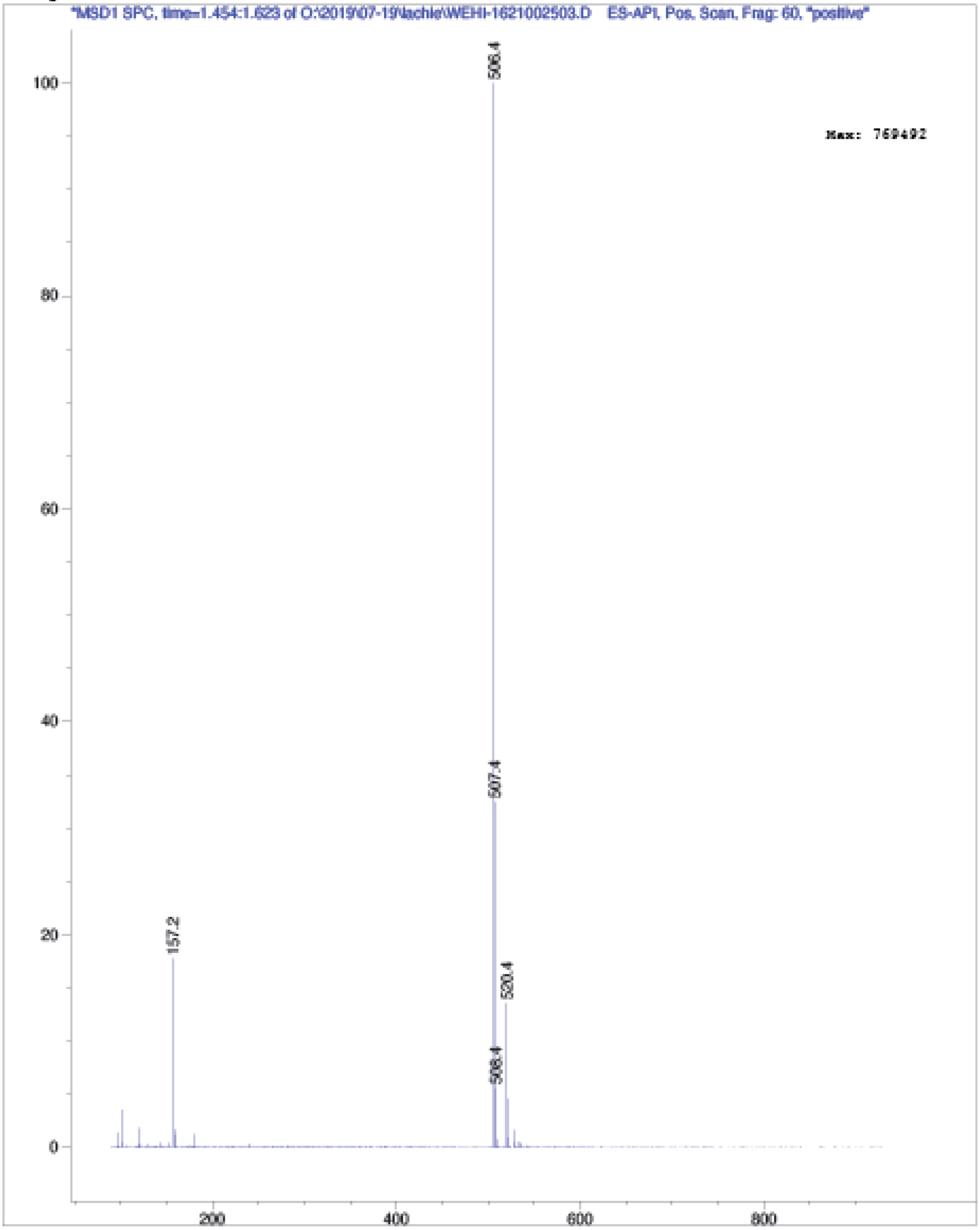

*MSD1 SPC, time=1.454:1.623 of O:\2019\07-19\lachie\WEHI-1621002503.D   ES-API, Pos, Scan, Frag: 60, "positive"
Max: 769492
506.4
507.4
157.2
520.4
508.4
100
80
60
40
20
0
200
400
600
800

39 C NMR

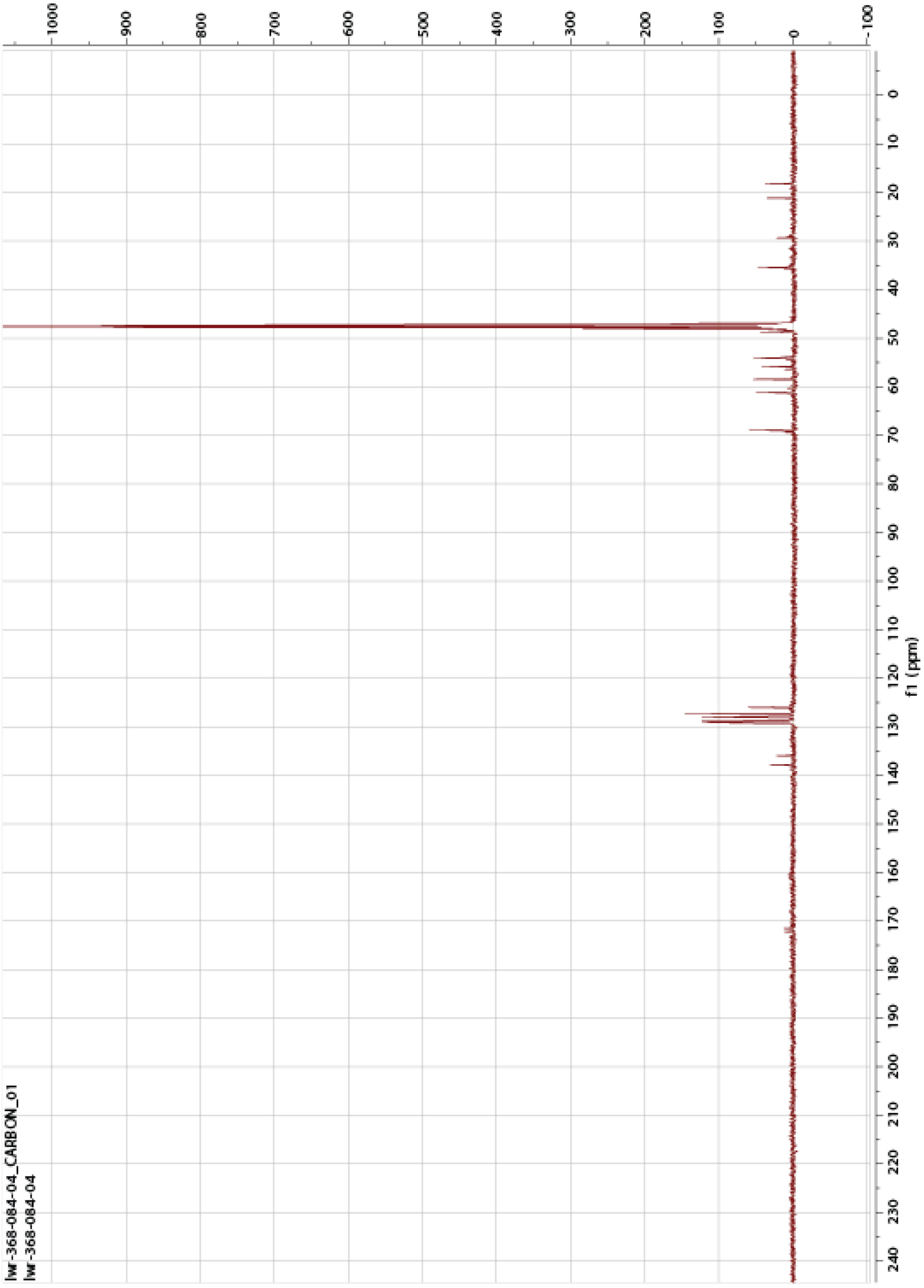

39 H NMR

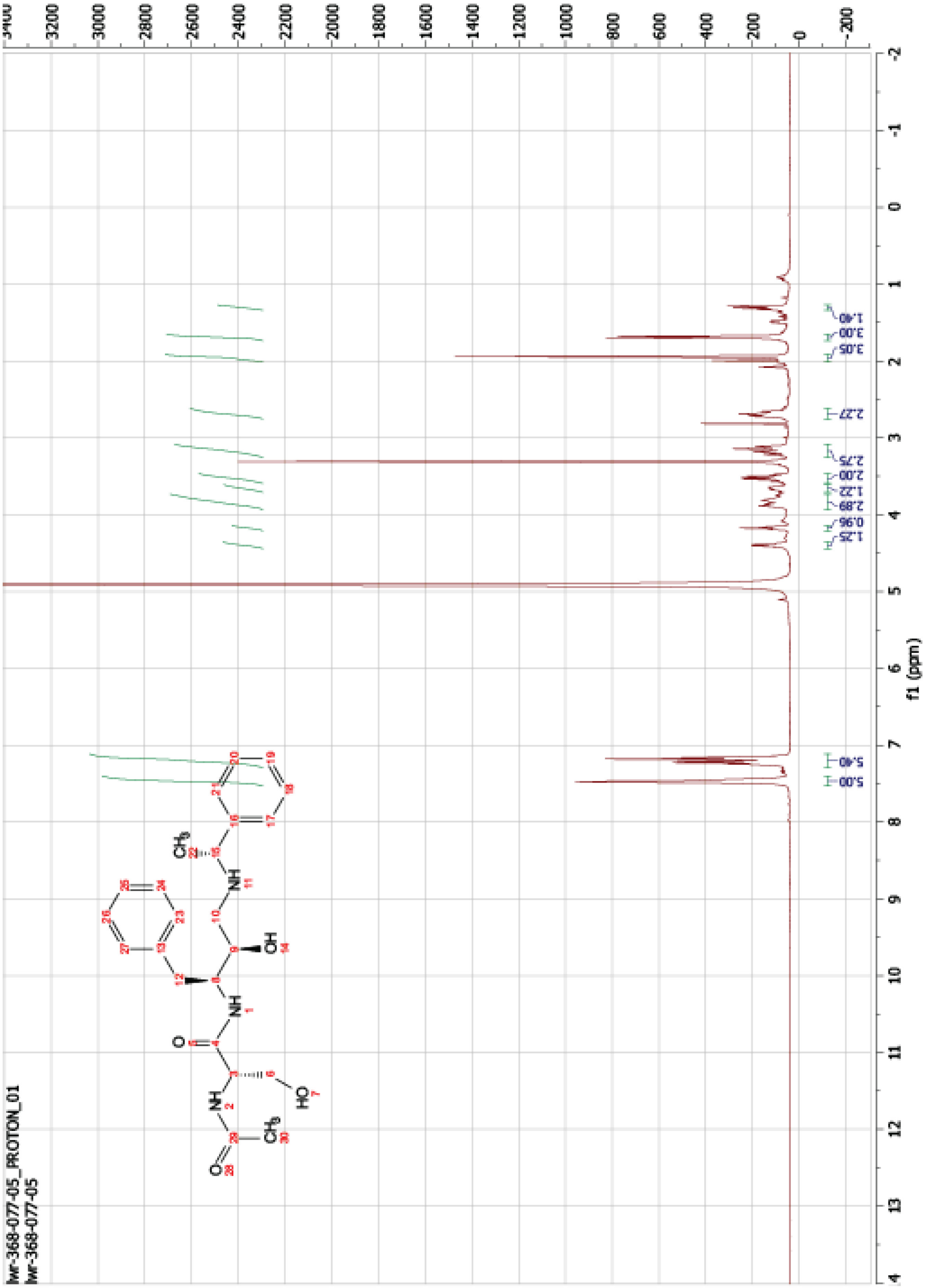

f

39 LCMS

Current Chromatogram(s)

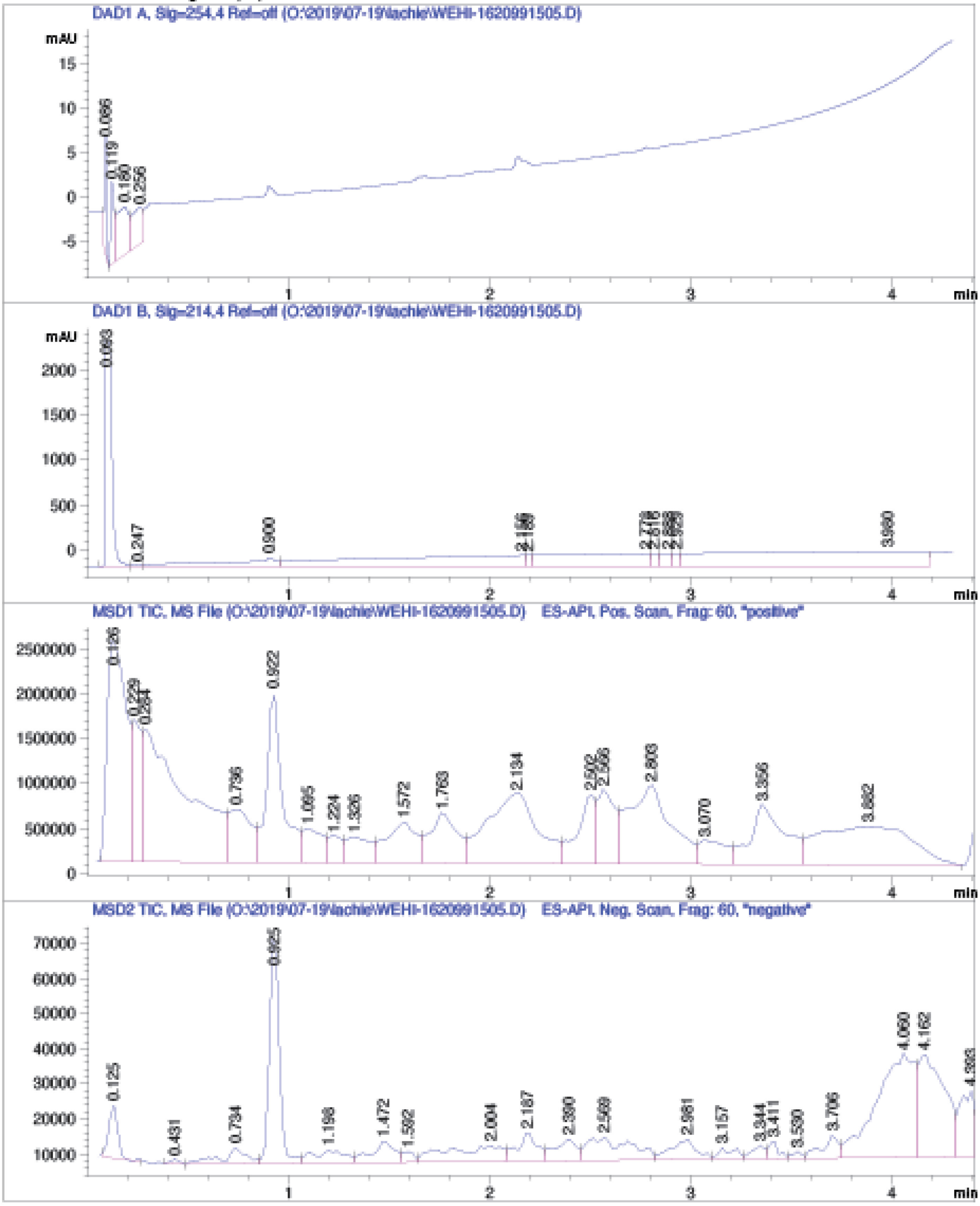

MS Spectrum

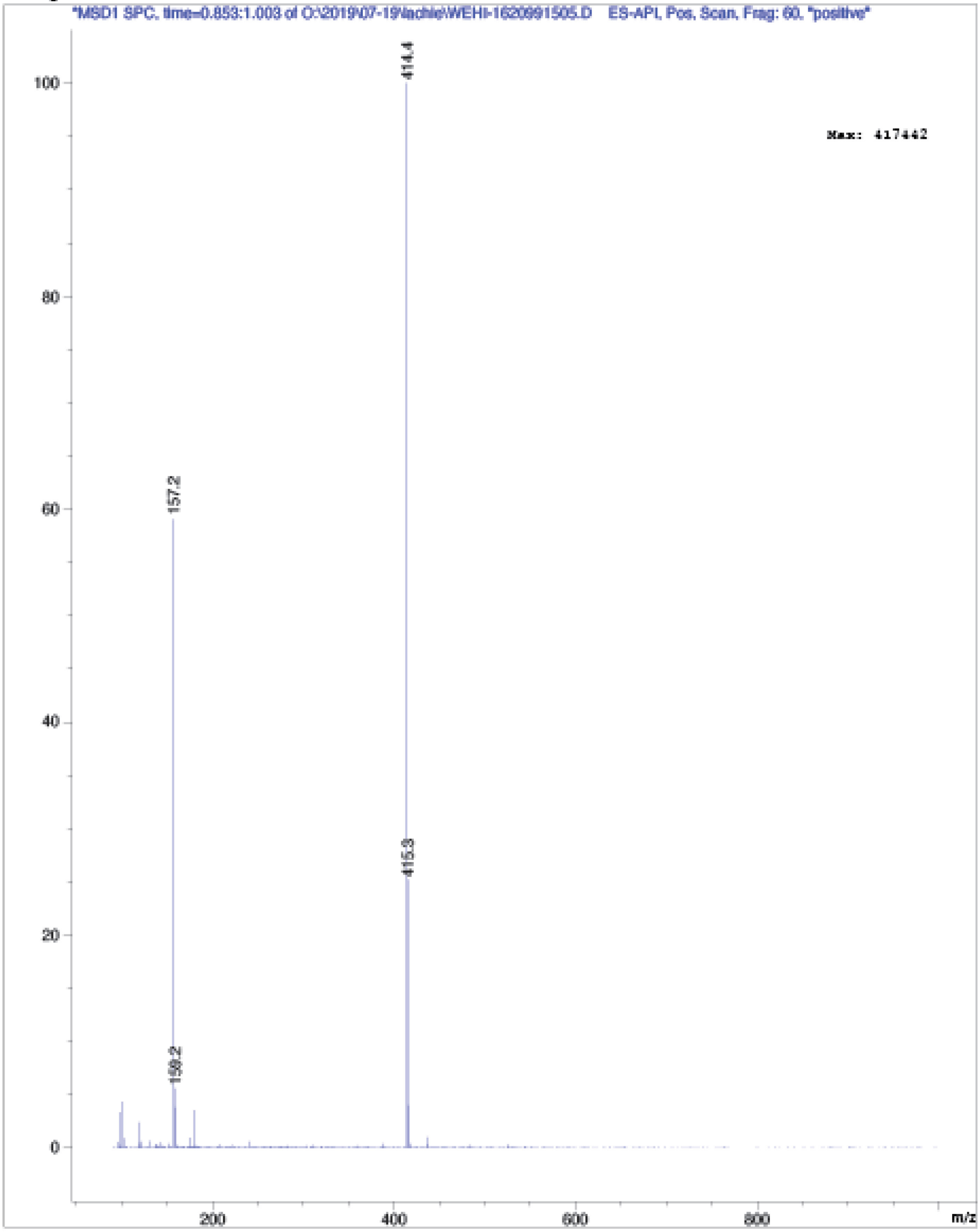

*MSD1 SPC, time=0.853:1.003 of O:\2019\07-19\lachie\WEHI-1620991505.D ES-API, Pos, Scan, Frag: 60, "positive"
Max: 417442
414.4
157.2
415.3
158.2
100
80
60
40
20
0
200
400
600
800
m/z

40 C NMR

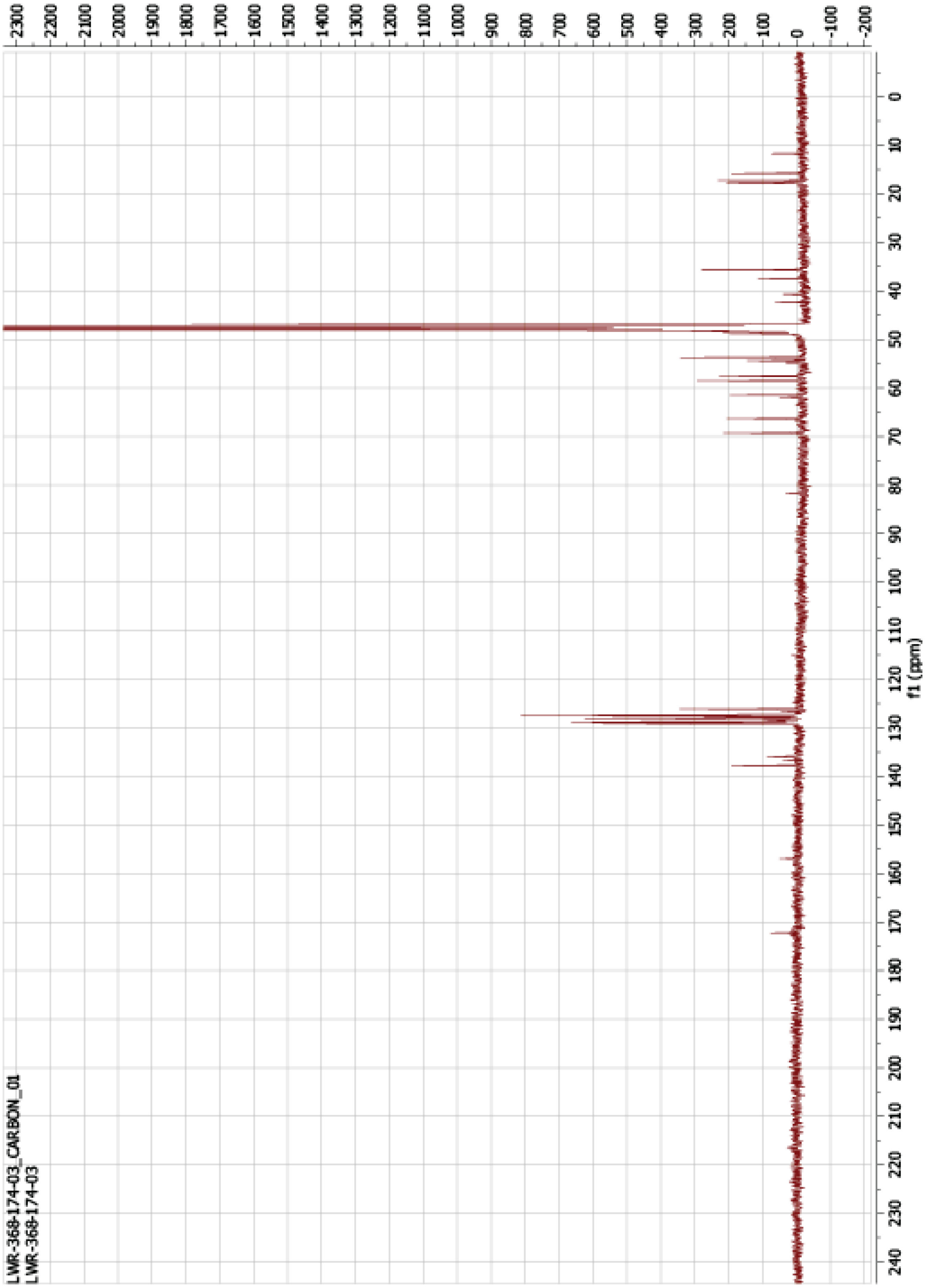

40 H NMR

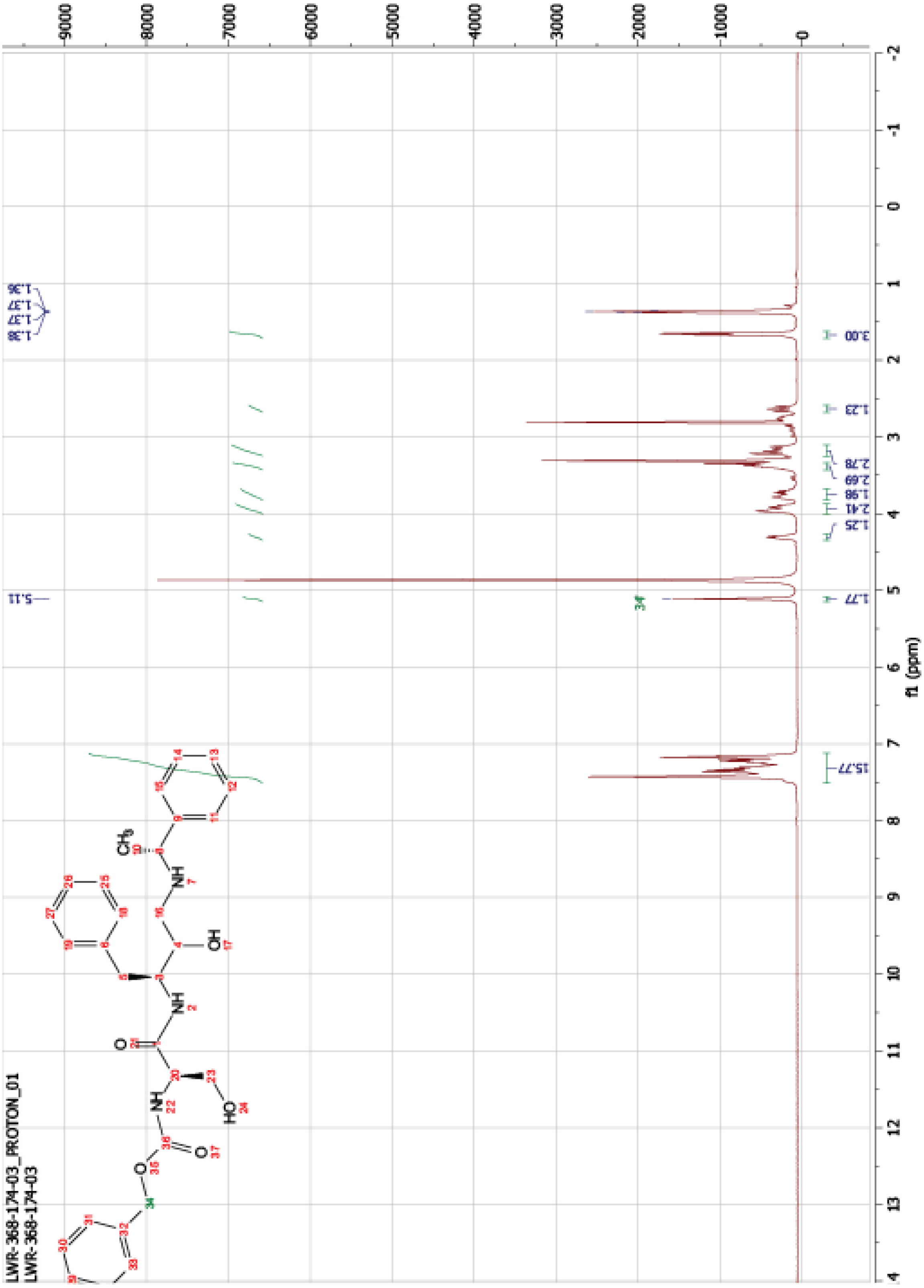

40 LCMS

Current Chromatogram(s)

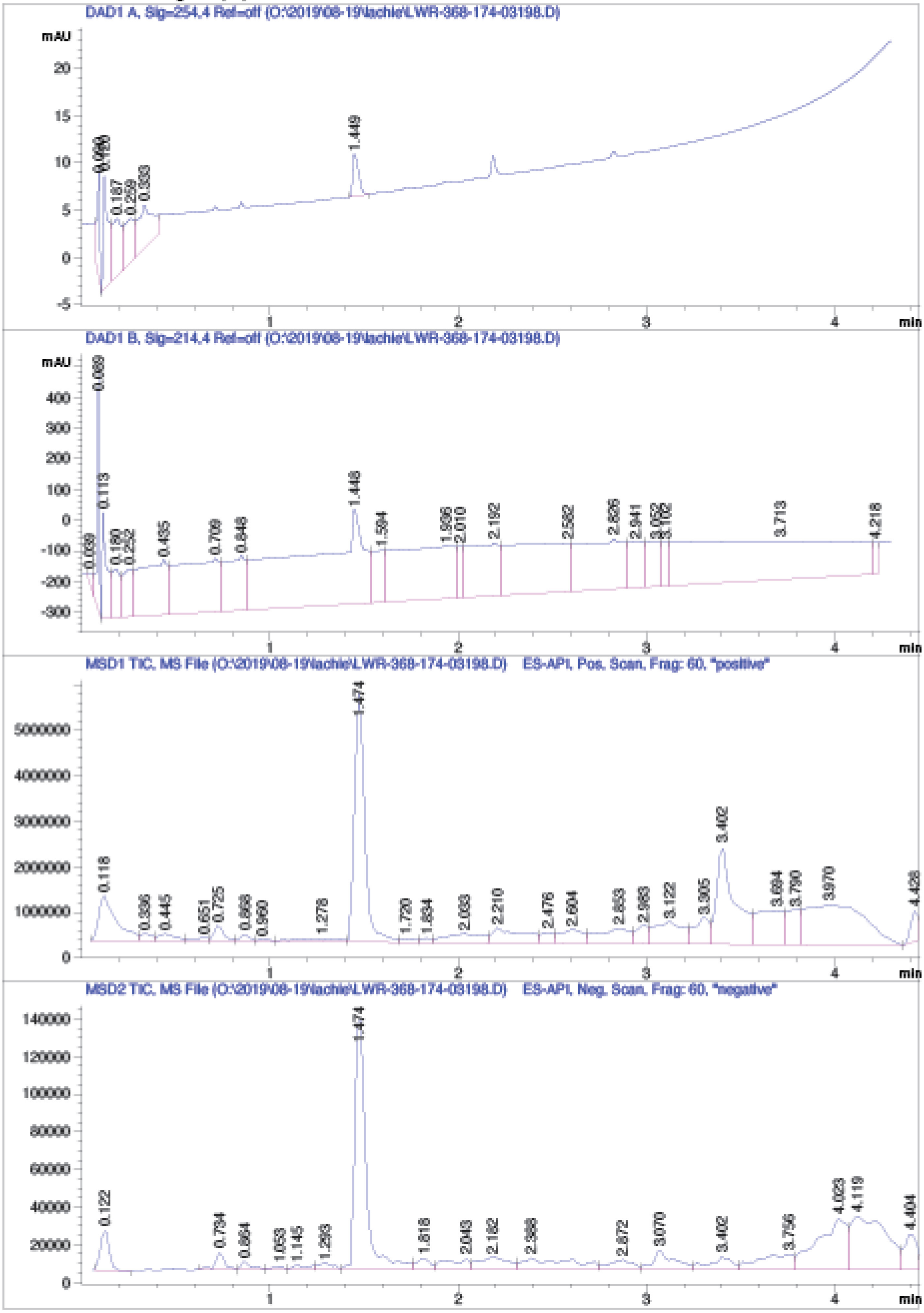

MS Spectrum

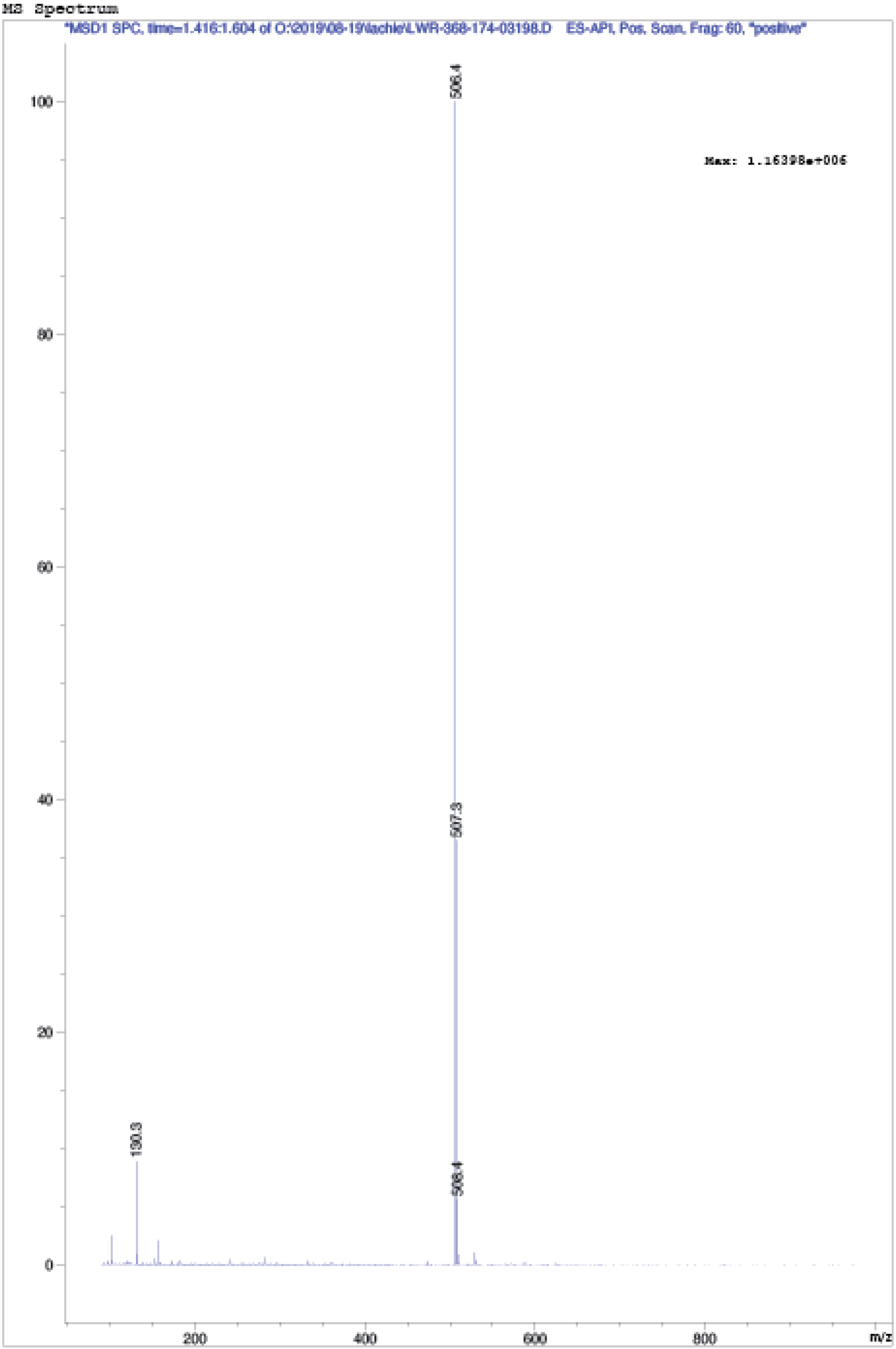
*MSD1 SPC, time=1.416:1.604 of O:\2019\08-19\lachie\LWR-368-174-03198.D ES-API, Pos, Scan, Frag: 60, "positive"
Max: 1.16398e+006
506.4
507.3
508.4
130.3
100
80
60
40
20
0
200
400
600
800
m/z

41 C NMR

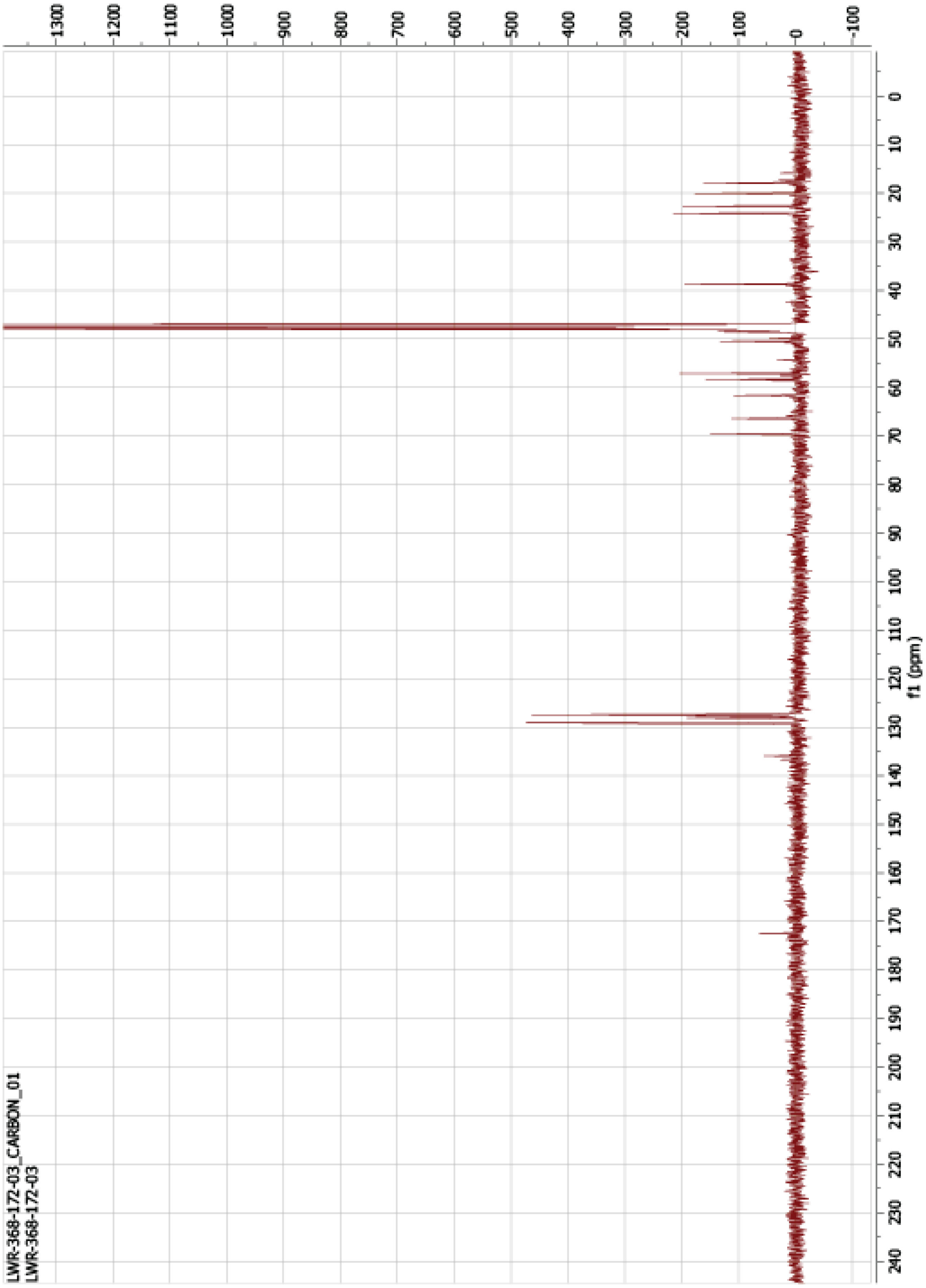

41 H NMR

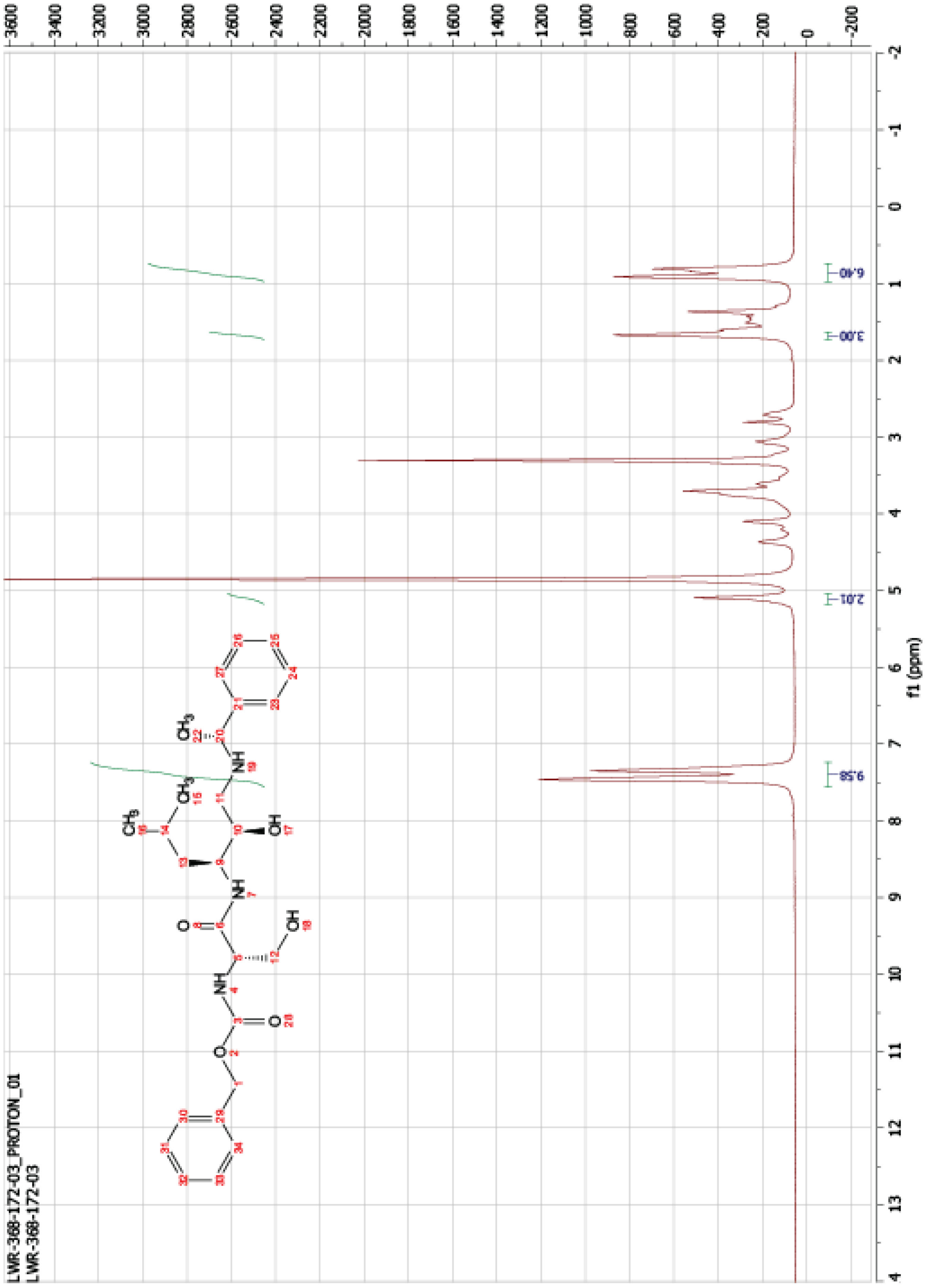

41 LCMS

Current Chromatogram(s)

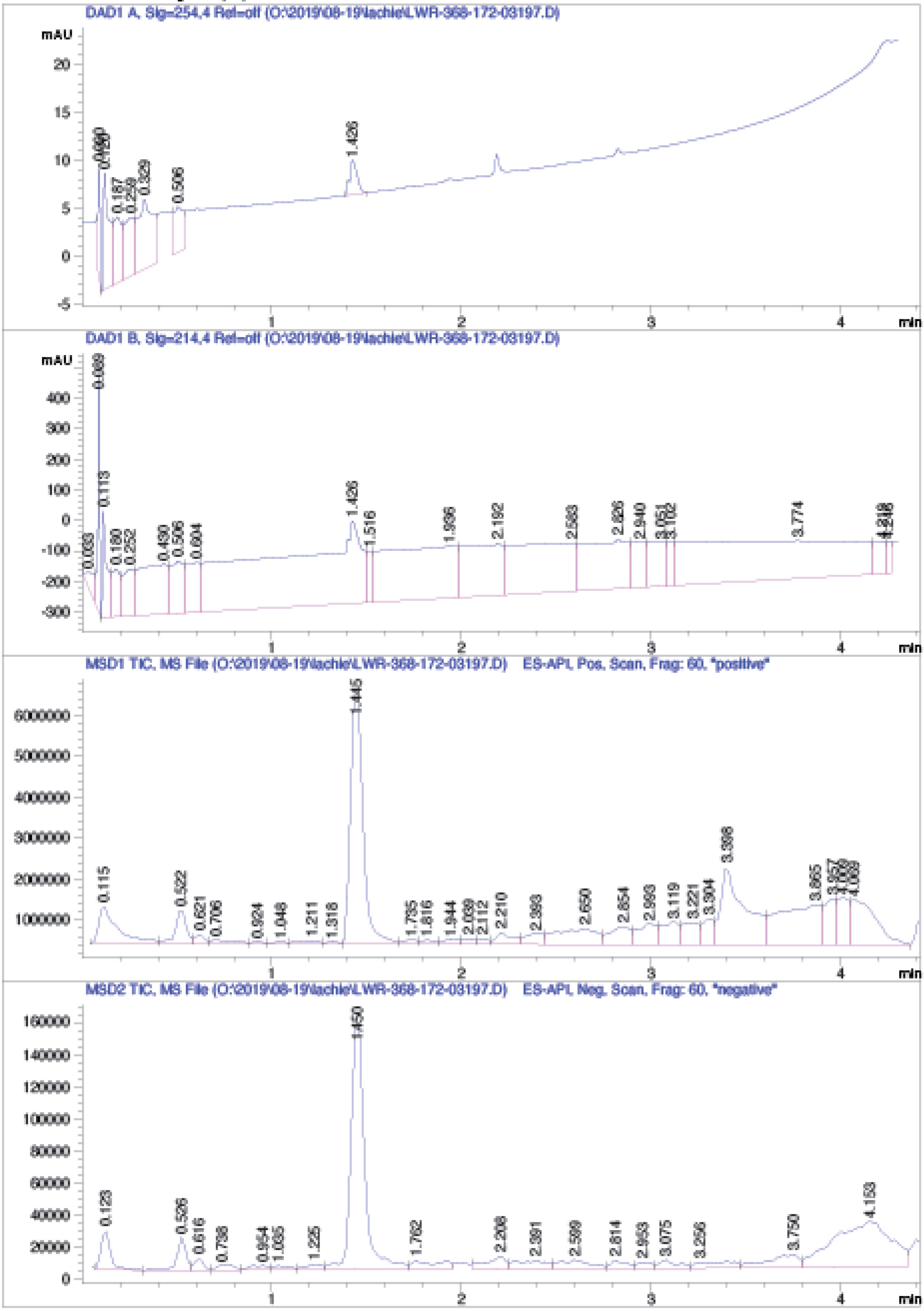

MS Spectrum

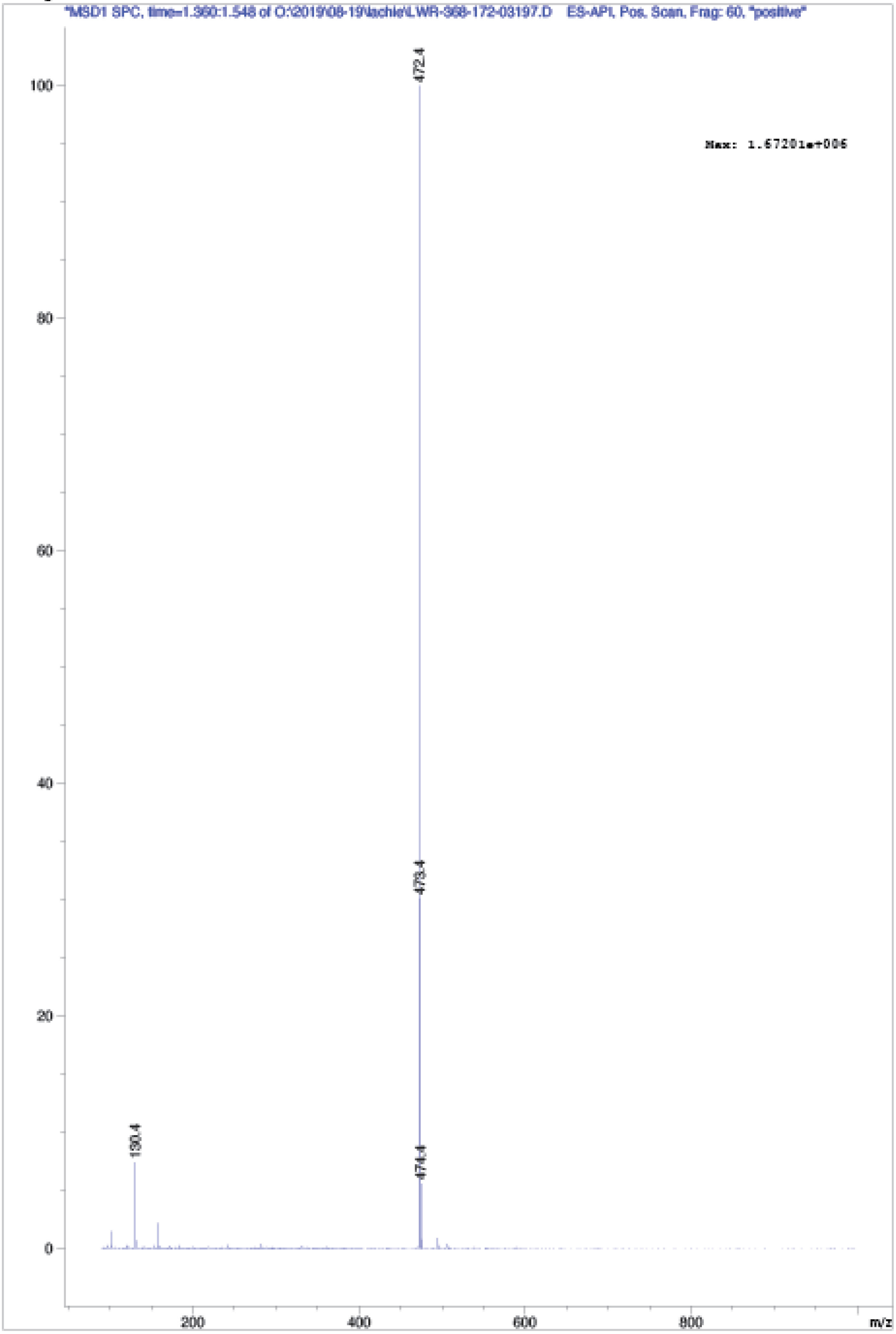

*MSD1 SPC, time=1.360:1.548 of O:\2019\08-19\achie\LWR-368-172-03197.D ES-API, Pos, Scan, Frag: 60, "positive"
Max: 1.67201e+006
472.4
473.4
474.4
130.4
100
80
60
40
20
0
200
400
600
800
m/z

42 C NMR

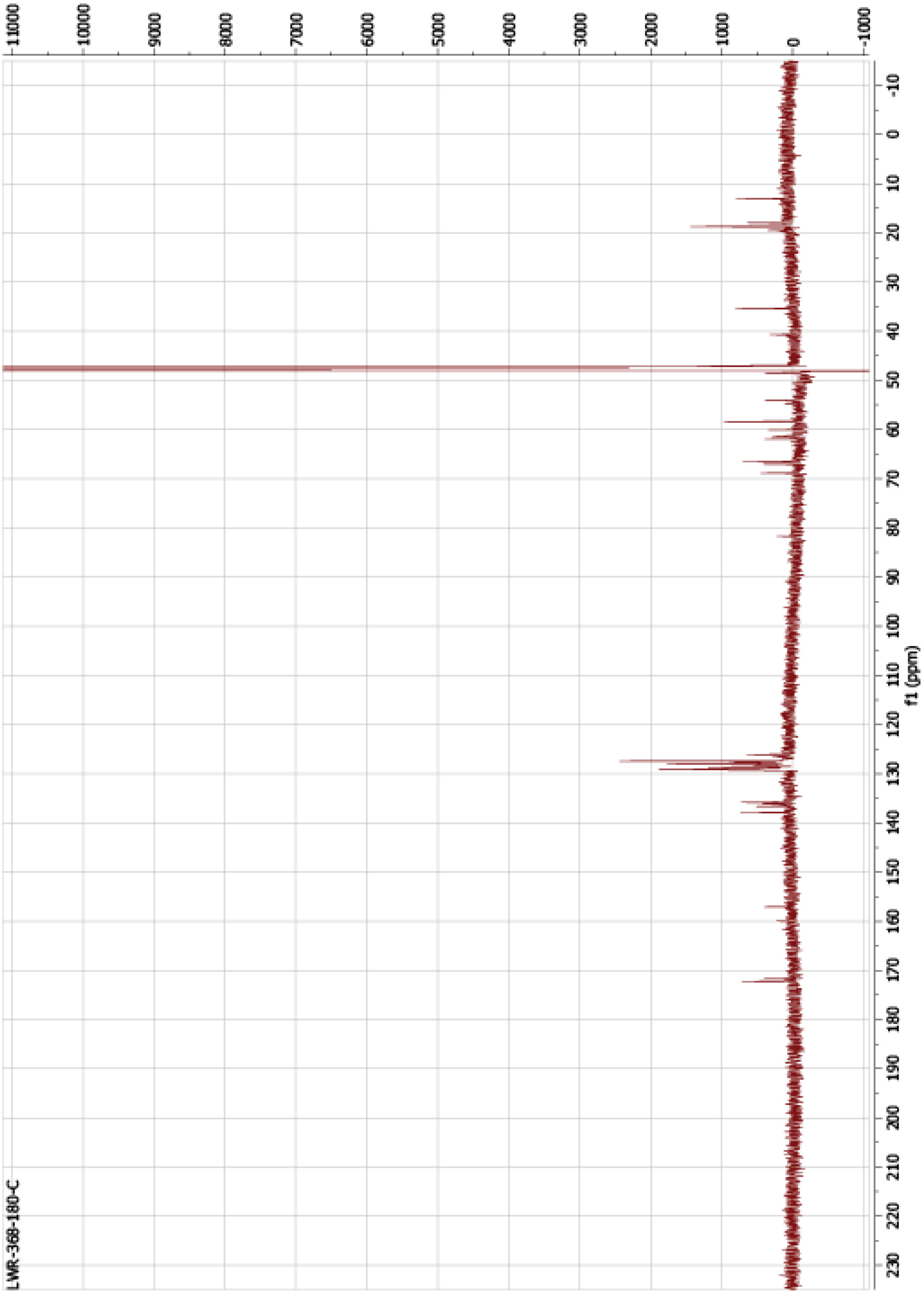

42 H NMR

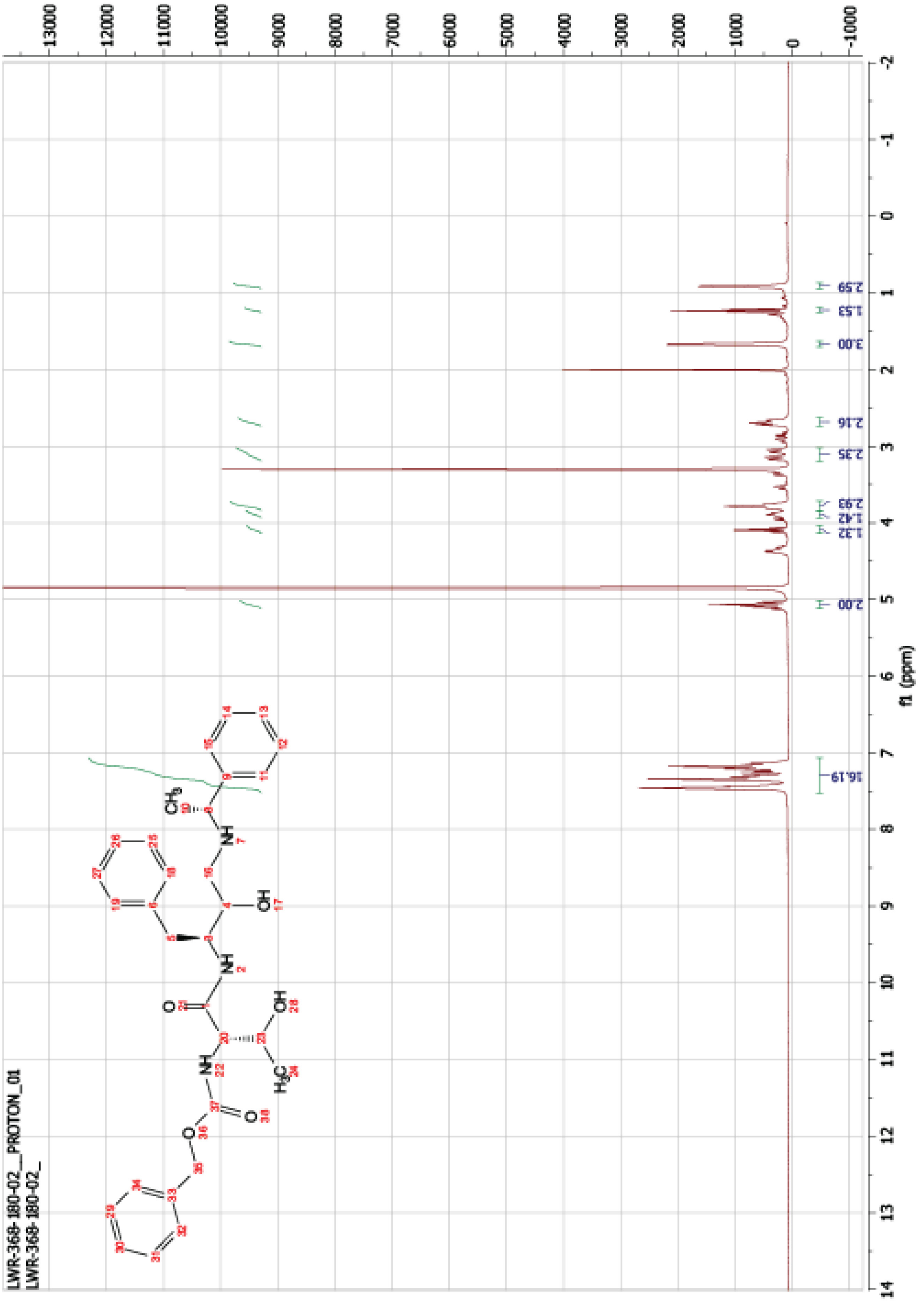

42 LCMS

Current Chromatogram(s)

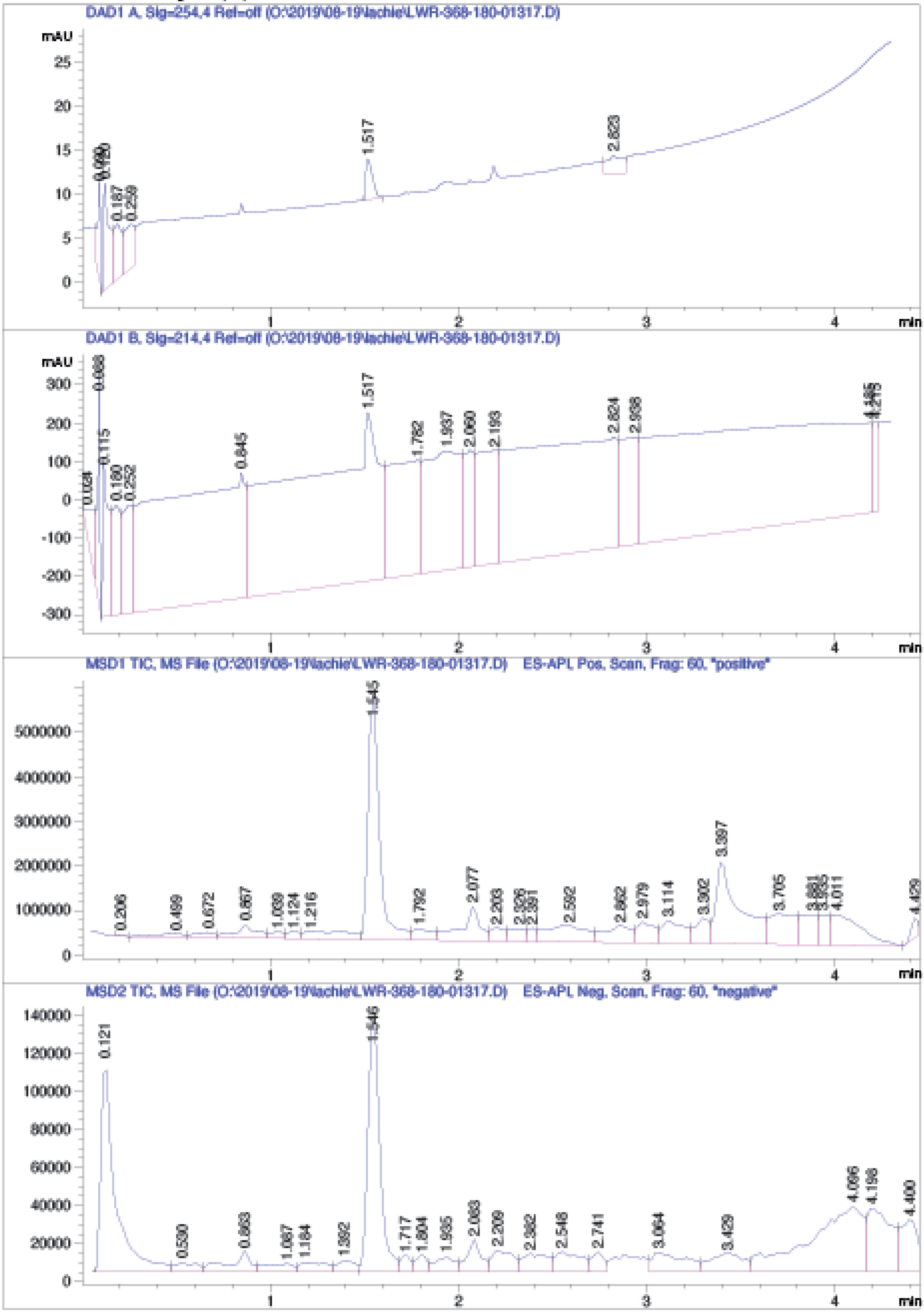

MS Spectrum

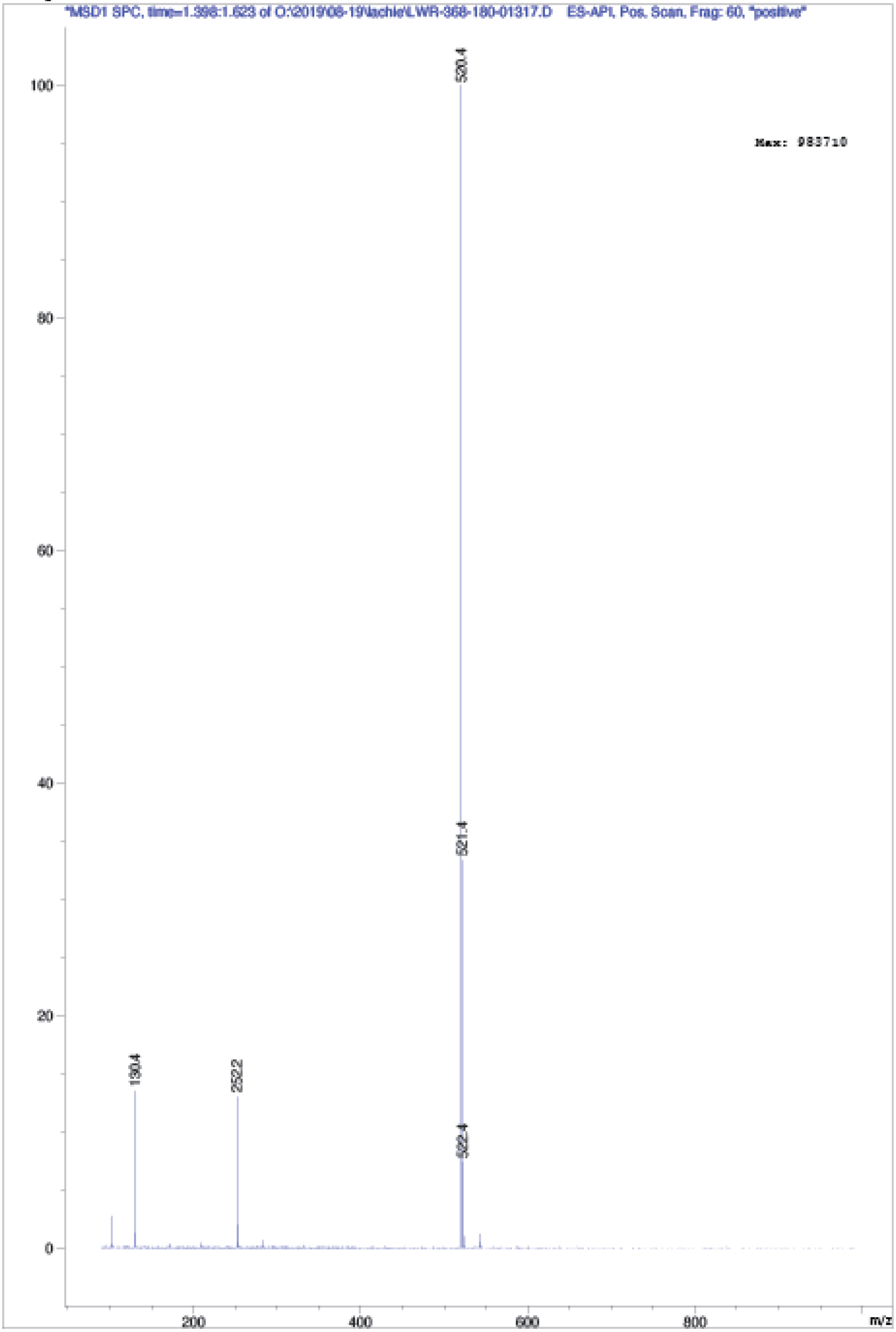
*MSD1 SPC, time=1.398:1.623 of O:\2019\08-19\lachie\LWR-368-180-01317.D ES-API, Pos, Scan, Frag: 60, "positive"
Max: 983710
520.4
521.4
522.4
130.4
252.2
100
80
60
40
20
0
200
400
600
800
m/z

References

[1] T. U. Consortium, *Nucleic Acids Res.* **2021**, *49*, D480-D489.

[2] A. N. Hodder, J. J. Christensen, S. Scally, T. Triglia, A. Ngo, R. W. Birkinshaw, B. Bailey, P. Favuzza, M. H. Dietrich, W. H. Tham, P. E. Czabotar, K. Lowes, K. Guo, N. Murgolo, M. L. Ruiz, J. A. McCauley, B. E. Sleebs, D. B. Olsen, A. F. Cowman, *Structure* **2022**, 30(7), 947-961.

[3] G. E. Crooks, G. Hon, J.-M. Chandonia, S. E. Brenner, *Genome Res.* **2004**, *14*, 1188-1190.

[4] S. W. Kortum, T. E. Benson, M. J. Bienkowski, T. L. Emmons, D. B. Prince, D. J. Paddock, A. G. Tomasselli, J. B. Moon, A. LaBorde, R. E. TenBrink, *Bioorg. Med. Chem. Lett.* **2007**, *17*, 3378-3383.

[5] P. Pino, R. Caldelari, B. Mukherjee, J. Vahokoski, N. Klages, B. Maco, C. R. Collins, M. J. Blackman, I. Kursula, V. Heussler, M. Brochet, D. Soldati-Favre, *Science* **2017**, *358*, 522-528.

[6] A. N. Hodder, B. E. Sleebs, P. E. Czabotar, M. Gazdik, Y. Xu, M. T. O'Neill, S. Lopaticki, T. Nebl, T. Triglia, B. J. Smith, K. Lowes, J. A. Boddey, A. F. Cowman, *Nat. Struct. Mol. Biol.* **2015**, *22*, 590-596.

[7] J. Maibaum, S. Stutz, R. Göschke, P. Rigollier, Y. Yamaguchi, F. Cumin, J. Rahuel, H.-P. Baum, N.-C. Cohen, C. R. Schnell, W. Fuhrer, M. G. Gruetter, W. Schilling, J. M. Wood, *J. Med. Chem.* **2007**, *50*, 4832-4844.

[8] J. D. Scott, S. W. Li, A. P. J. Brunskill, X. Chen, K. Cox, J. N. Cumming, M. Forman, E. J. Gilbert, R. A. Hodgson, L. A. Hyde, Q. Jiang, U. Iserloh, I. Kazakevich, R. Kuvelkar, H. Mei, J. Meredith, J. Misiaszek, P. Orth, L. M. Rossiter, M. Slater, J. Stone, C. O. Strickland, J. H. Voigt, G. Wang, H. Wang, Y. Wu, W. J. Greenlee, E. M. Parker, M. E. Kennedy, A. W. Stamford, *J. Med. Chem.* **2016**, *59*, 10435-10450.
